# Performance of Rapid Diagnostic Tests for Imported Malaria in Clinical Practice: Results of a National Multicenter Study

**DOI:** 10.1371/journal.pone.0075486

**Published:** 2013-09-30

**Authors:** Sandrine Houzé, Isabelle Boutron, Anne Marmorat, Marie Dalichampt, Christophe Choquet, Isabelle Poilane, Nadine Godineau, Anne-Sophie Le Guern, Marc Thellier, Hélène Broutier, Odile Fenneteau, Pascal Millet, Stéphanie Dulucq, Véronique Hubert, Pascal Houzé, Florence Tubach, Jacques Le Bras, Sophie Matheron

**Affiliations:** 1 AP-HP (Assistance Publique des Hôpitaux de Paris), Hôpital Bichat, Laboratoire de Parasitologie-Centre National de Référence du Paludisme, Paris, France; 2 Université Paris Descartes, UMR 216, Paris, France; 3 Institut de Recherche pour le Développement, UMR 216, Paris, France; 4 AP-HP (Assistance Publique des Hôpitaux de Paris), Hôpital Hôtel Dieu, Centre d’Épidémiologie Clinique, Paris, France; 5 Université Paris Descartes, Sorbonne Paris Cité, Faculté de Médecine, Paris, France; 6 AP-HP (Assistance Publique des Hôpitaux de Paris), Hôpital Bichat, Service des Urgences, Paris, France; 7 AP-HP (Assistance Publique des Hôpitaux de Paris), CHU Jean-Verdier, Laboratoire de Microbiologie, Bondy, France; 8 Hôpital Delafontaine, Laboratoire de Parasitologie, St-Denis, France; 9 Institut Pasteur, Laboratoire du Centre Médical, Paris, France; 10 AP-HP (Assistance Publique des Hôpitaux de Paris), Hôpital de la Pitié-Salpêtrière, Laboratoire de Parasitologie, Paris, France; 11 Hôpital Robert-Ballenger, Laboratoire polyvalent, Aulnay-sous-Bois, France; 12 AP-HP (Assistance Publique des Hôpitaux de Paris), Hôpital Robert Debré, Laboratoire d’Hématologie, Paris, France; 13 Hôpital Saint André, Laboratoire de Parasitologie - Mycologie, Bordeaux, France; 14 Université Bordeaux Segalen, EA 4575, Bordeaux, France; 15 Hôpital Pellegrin, Laboratoire de Biologie, Bordeaux, France; 16 AP-HP (Assistance Publique des Hôpitaux de Paris), Hôpital St-Louis, Laboratoire de Biochimie, Paris, France; 17 AP-HP (Assistance Publique des Hôpitaux de Paris), Hôpital Bichat, Département d’Epidémiologie, Biostatistique et Recherche Clinique, Paris, France; 18 Université Paris 7 Denis Diderot, Paris, France; 19 INSERM 801, Paris, France; 20 AP-HP (Assistance Publique des Hôpitaux de Paris), Hôpital Bichat, Service des Maladies Infectieuses et Tropicales, Paris, France; Royal Tropical Institute, Netherlands

## Abstract

We compared the performance of four rapid diagnostic tests (RDTs) for imported malaria, and particularly *Plasmodium falciparum* infection, using thick and thin blood smears as the gold standard. All the tests are designed to detect at least one protein specific to *P. falciparum* ( *Plasmodium* histidine-rich protein 2 (PfHRP2) or *Plasmodium* LDH (PfLDH)) and one pan-*Plasmodium* protein (aldolase or Plasmodium LDH (pLDH)). 1,311 consecutive patients presenting to 9 French hospitals with suspected malaria were included in this prospective study between April 2006 and September 2008. Blood smears revealed malaria parasites in 374 cases (29%). For the diagnosis of *P. falciparum* infection, the three tests detecting PfHRP2 showed high and similar sensitivity (96%), positive predictive value (PPV) (90%) and negative predictive value (NPV) (98%). The PfLDH test showed lower sensitivity (83%) and NPV (80%), despite good PPV (98%). For the diagnosis of non-*falciparum* species, the PPV and NPV of tests targeting pLDH or aldolase were 94–99% and 52–64%, respectively. PfHRP2-based RDTs are thus an acceptable alternative to routine microscopy for diagnosing *P. falciparum* malaria. However, as malaria may be misdiagnosed with RDTs, all negative results must be confirmed by the reference diagnostic method when clinical, biological or other factors are highly suggestive of malaria.

## Introduction

Malaria is endemic in 99 countries worldwide, and several million people travel from non-endemic countries to malaria-endemic regions each year [1]. Although more than 7000 cases of imported malaria are reported annually both in European countries and in the United States, malaria remains unusual in non-endemic countries and its clinical presentation is often nonspecific [2,3]. Misdiagnosis which remains too frequent, resulting in delays in antimalarial treatment [4]. A careful physical examination and interview, including any recent travel to a malaria-endemic region, are crucial for diagnostic evaluation. More than 90% of cases of imported malaria are due to *Plasmodium falciparum*, the species associated with severe morbidity and mortality. The remaining cases are due to *P. ovale*, *P. vivax*, *P. malariae* or, rarely, *P* *. knolewsi* , alone or in association with *P. falciparum* [4].

Malaria is a serious disease that must be diagnosed urgently. The reference routine test is microscopic examination of Giemsa-stained blood smears [5]. However, many centers cannot provide reliable round-the-clock smear-based diagnosis. Thus, a simple, sensitive test capable of reliably confirming or ruling out malaria would be a welcome addition to the diagnostic arsenal.

Rapid diagnostic tests (RDTs) that detect malaria parasite proteins by immunochromatography were first developed 20 years ago as a complement to microscopic diagnosis [6]. RDTs detect a variety of proteins, including *P. falciparum* histidine-rich protein 2 (PfHRP2) and *P. falciparum* lactate dehydrogenase (PfLDH), both specific to *P. falciparum*; and also Plasmodium LDH (pLDH) and aldolase, enzymes shared by the 5 human pathogenic *Plasmodium* species [6]. Such tests are now increasingly used outside of malaria-endemic areas [7–9]. Numerous studies including a Cochrane meta-analysis assessed the accuracy of RDTs for diagnosis of malaria in endemic settings [10–14]. Studies were conducted in Europe among travelers returning from endemic areas [15,16] but results are limited to *P. ovale, P. malariae* and *P. vivax*, and shown large differences in performance [9]. Tests available in non-endemic countries bear the CE (European Conformity) label but their performance is extremely variable; in addition, they have mainly been tested in endemic areas and have never been compared in the same study [17].

In 2008, WHO launched a comparative study of some RDTs on selected samples with *P. falciparum* and *P. vivax* [18] but studies using clinical samples are the most informative ones regarding test performance in routine use. Previous studies of patients presenting to emergency rooms in Europe with suspected imported malaria were all retrospective and compared only 1 or 2 RDTs with standard microscopic methods [15,16,19–21]. We report the results of a multicenter study of the diagnostic performance of four RDTs selected among those most widely used in Europe to diagnose imported malaria, performed in usual care settings in non-endemic areas. Thick and thin blood smears were considered as the gold standard and discordant results were explored in an attempt to obtain an analytical explanation.

## Methods

### Study design

This prospective study included patients with fever or history of fever and a history of travel to a malaria-endemic area and who underwent diagnostic tests in one of 9 French hospitals (from April 2006 to September 2008 in 6 centers and from September 2007 to September 2008 in 3 centers) (Table 1) (Protocol summary S1, Study protocol S1). The study was designed, conducted and reported in compliance with the Standards for the Reporting of Diagnostic Accuracy (STARD) guidelines [22] (STARD checklist S1).

**Table 1:** Characteristics of the participating centers.

**Participating center**	**Category of hospital**	**Patients**	**Number of suspected cases of malaria per year**	**Number of malaria diagnoses per year**	**Number of patients included in the study (%) (n=1311)**
Bichat hospital	University Hospital	Adults	752	126 (16.8%)	571 (44.3%)
Jean-Verdier hospital	University hospital	Children + adults	200	40 (20%)	195 (15.1%)
Delafontaine hospital	General hospital	Children + adults	719	95 (13.2%)	145 (11.3%)
Institut Pasteur	Travel clinic	Adults	650	25 (3.8%)	97 (7.5%)
Pitié-Salpêtrière hospital	University Hospital	Adults	1021	90 (8.8%)	69 (5.4%)*
Robert-Ballanger hospital	General hospital	Children + adults	345	57 (16.5%)	68 (5.3%)
Robert-Debré hospital	University Hospital	Children	670	65 (9.7%)	63 (4.9%)
Pellegrin + Saint-André hospitals	University hospital	Children + adults	600	80 (13.3%)	80 (6.1%)*

* These sites participated in the study for 12 months only

### Ethics Statement

This research was non interventional and, in accordance with French legislation (article R1121-2 of the French public health code), was authorized by two French authorities (CCTIRS and CNIL) and registered with the Ile-de-France XI ethics committee under identification number 06080 (Report of ethic committee S1, ANRS recommendation research study S1). No authorization was required from the latter body, in accordance with French legislation on non-interventional research. Patients (or the parents of minors) were individually informed, through a written document (approved by both CCTIRS and CNIL), and their non-objection to participation was systematically collected in their medical files. The study is registered with Clinical Trials.gov (identifier NCT00451269) (http://www.clinicaltrials.gov/).

### Patients

Patients attending a participating center with fever or history of fever and with a history of travel to malaria-endemic areas leading to prescription of laboratory tests for malaria were prospectively considered for enrollment. After reading a dedicated information sheet, patients were excluded if they (or the parents of minors) declined to participate. The non-objection to participation was systematically collected by investigators and recorded in the medical files of all participants. Patients were recruited in 8 hospitals (five university hospitals, three general hospitals) with emergency rooms or infectious and tropical diseases departments, and in one private hospital. These centers managed between 25 and 126 cases of malaria each year, in children and/or adults (Table 1). Patient participation in the study was limited to a single blood sample used both for the diagnosis of acute malaria and for other relevant tests. A physician collected the patient’s age, gender, birth country, country of residence, country of travel, date of return, malaria chemoprophylaxis, and any antimalarial treatment taken before consulting.

### Preparation and reading of smears

The same venous blood sample taken in an anticoagulant (EDTA)-containing tube was used for blood smears and the four RDTs. Patient care decisions were based on the results of routine methods performed in each center, using the same venous blood sample. The 4 RDTs were performed simultaneously by technicians in each participating center. A sample of the same blood was immediately sent to the French National Malaria Reference Center (FNMRC), where reference thin and thick blood smears were prepared and stained upon receipt. Each slide was read by one of the three expert microscopists involved in this study, who were blinded to the patients’ characteristics and symptoms and to the results of the RDTs [23]. The results of the expert readings were not compared with those of the non-centralized readings. Thin smears prepared at the FNMRC were considered positive for malaria if one or more malaria parasites were visualized, and negative if no asexual form of *Plasmodium* was observed in 200 high-power fields (about 40 000 erythrocytes). Parasite density was expressed as the percentage of infected red cells. Thick blood smears prepared at FNMRC were considered positive if one or more malaria parasites were visualized and negative if no parasites were detected after examining 1000 white blood cells. The parasite species was determined. The isolated presence of *P. falciparum* gametocytes was noted but was not considered indicative of acute malaria.

To assess the reproducibility of the centralized microscopic examination, smears of 30 blood samples were read independently and blindly by a biologist who had no other role in the study.

### Rapid diagnostic tests

All 4 tests were capable of detecting at least one *P. falciparum*-specific protein and one pan-*Plasmodium* protein. The Now ICT Malaria test (PfHRP2-test_1_, pan-aldolase test) (manufactured by Binax, distributed by Inverness, France) detects *P. falciparum* PfHPR2 and the aldolase of the 5 human *Plasmodium* species. The Core Malaria Pan/Pv/Pf test (PfHRP2-test_2_, pLDH-test_2_) (Ivagen, France) and the Palutop+4 test (PfHRP2-test_3,_ pLDH-test_3_) (All Diag, France) detect PfHRP2, *P. vivax* PvLDH, and pLDH of the 5 human *Plasmodium* species. The Optimal-IT test (PfLDH-test, pLDH-test_1_) (Diamed, France) detects *P. falciparum* PfLDH and pLDH. The tests were based on lateral flow immunochromatography, in either cassette format (PfLDH-test, PfHRP2-test_2_, and PfHRP2-test_3_) or card format (PfHRP2-test_1_) (Table 2). PfHRP2-test_1_ and PfLDH-test are three-band tests, while PfHRP2-test_2_ and PfHRP2-test_3_ are four-band tests. All 4 tests include a control line that must be present for the test to be valid. The kits were stored in a dry environment between 18°C and 25°C.

**Table 2:** Names and targets of the rapid diagnostic tests for malaria.

**Test**	**Name**	**Distributor in France**	**Target**	**Species**
PfHRP2-test_1_	Now ICT Malaria	Inverness	PfHRP2	*Plasmodium falciparum*
pan-aldolase test			Aldolase	All species
PfHRP2-test_2_	Core Malaria	Ivagen	PfHRP2	*P. falciparum*
pLDH-test_2_	Pan/Pv/Pf		pLDH	All species
			PvLDH	*Plasmodium vivax*
PfHRP2-test_3_	Palutop +4	All Diag	PfHRP2	*P. falciparum*
pLDH-test_3_			pLDH	All species
			PvLDH	*P. vivax*
PfLDH test	Optimal IT	Diamed	PfLDH	*P. falciparum*
pLDH-test_1_			pLDH	All species

The tests were performed according to the manufacturers’ instructions. Blood samples (5, 10 or 15 µl, depending on the test) were loaded into a transfer pipette. Samples and diluents were applied and reading was performed 10 to 20 minutes later, depending on the test. All the results were read by local trained technicians blinded to the results of the standard test. If the control line did not appear, the test was considered invalid and was repeated. Results were scored as negative (no test line visible) or positive (at least one test line visible). If the test line was barely visible, the result was scored as doubtful but was considered positive in subsequent analyses.

### Inconsistencies

If the *Plasmodium* species could not be reliably identified on the FNMRC centralized thin blood smear, species PCR was performed by FNRMRC, as previously described, on DNA extracted from the EDTA sample, with primers specific for each of the five species of plasmodium [24,25]. In case of discrepancies between a positive RDT and negative blood smears, PCR was used to detect plasmodial DNA, as evidence of previous *Plasmodium* infection or submicroscopic parasitemia.

As parasite viability may influence the performance of pLDH-based tests [26], antimalarial drugs (chloroquine and its metabolite, amodiaquine and its metabolite, quinine, proguanil and its metabolite, mefloquine and doxycycline and its metabolite) were assayed by high-performance liquid chromatography on EDTA plasma to detect prior treatment when Pf-LDH- or pLDH-based RDTs were negative but blood smears were positive.

**Table 3:** Performance of the four rapid diagnostic tests according to their target antigens and *Plasmodium* species.

**Species**	**Target**		**PfHRP2-test_1_**	**PfHRP2-test_2_**	**PfHRP2-test_3_**	**PfLDH**
**N = 1237**			**(Now ICT Malaria)**	**(Core Malaria)**	**(Palutop +4)**	**(Optimal-IT)**
**All positive**		Sensitivity	0.93	0.94	0.94	0.83
**samples**			[0.90-0.96]	[0.91-0.96]	[0.92-0.97]	[0.79-0.86]
		Specificity	0.97	0.96	0.97	0.99
			[0.96-0.98]	[0.95-0.97]	[0.96-0.98]	[0.99-1.00]
**Species**	**Target**		**PfHRP2-test_1_**	**PfHRP2-test_2_**	**PfHRP2-test_3_**	**PfLDH**
**N = 1237**			**(Now ICT Malaria)**	**(Core Malaria)**	**(Palutop +4)**	**(Optimal-IT)**
***P. falciparum***	***Pf*HRP2**	Sensitivity	0.96	0.96	0.96	0.83
***(alone or***	**or**		[0.94-0.98]	[0.94-0.98]	[0.94-0.98]	[0.79-0.87]
***mixed )***	**P*f*LDH**	Specificity	0.97	0.97	0.97	1
***infection***			[0.96-0.98]	[0.96-0.98]	[0.96-0.98]	[0.99-1]
		PPV	0.9	0.9	0.9	0.98
			[0.86-0.93]	[0.87-0.93]	[0.86-0.93]	[0.96-1]
		NPV	0.98	0.98	0.98	0.8
			[0.97-0.99]	[0.97-0.99]	[0.97-0.99]	[0.78-0.83]
		LR+	34.8	36.2	34.5	188
			[23.6-51.2]	[24.4-53.7]	[23.5-50.9]	[70.6-499]
		LR-	0.04	0.04	0.04	0.17
			[0.02-0.06]	[0.02-0.06]	[0.03-0.07]	[0.13-0.21]
**Species**	**Target**			***Pv*LDH**	***Pv*LDH**	
**N=1256**				**(CoreMalaria)**	**(Palutop +4)**	
***P. vivax***	***Pv*LDH**	Sensitivity		0.82	0.91	
				[0.59-1]	[0.74-1]	
		Specificity		0.99	0.99	
				[0.99-1]	[0.99-1]	
		PPV		0.9 7	0.98	
				[0.89-1]	[0.9-1]	
		NPV		0.79	0.89	
				[0.76-0.81]	[0.88-0.91]	
		LR+		145.5	162	
				[69.4-305.1]	[77.1-339]	
		LR-		0.18	0.09	
				[0.05-0.64]	[0.01-0.59]	

PPV: positive predictive value, NPV: negative predictive value, LR: *likelihood* *ratio*.

### Sample size calculation

To achieve an estimated RDT sensitivity of 95% with an accuracy of 0.03 (half the 95% confidence interval [95% CI]), we needed a test sample population of 245 patients with confirmed malaria. As the rate of confirmed malaria among patients with clinical signs was approximately 20% during the year preceding the study in all the participating centers, we targeted an enrollment of approximately 1225 patients.

### Statistical analysis

A patient was considered to have confirmed acute malaria in the presence of fever or a history of fever, and of asexual *Plasmodium* forms on a blood film. The isolated presence of *P. falciparum* gametocytes without asexual forms did not define a case of acute malaria [27]. The reproducibility of thin and thick blood smear reading was validated by using a Kappa concordance test. The performance of the four RDTs was compared to that of the routine “gold standard” test, i.e. centralized thin or thick blood smear, in terms of their sensitivity, specificity, positive and negative predictive values (PPV and NPV), and their 95% CIs for binomial proportions. Likelihood ratios were provided with 95% CIs calculated as risk ratios [28]. Cochran’s Q test was used to compare the sensitivity and specificity of the four RDTs. P values below 0.05 (two-tailed) were considered statistically significant. SAS v9-1 software (SAS Inst., Cary, NC, USA) was used for statistical analyses.

**Table 4:** Performance of the four rapid diagnostic tests for the detection of pan-antigen, pLDH and aldolase, according to the *Plasmodium* species and test.

**Species**	**Target**				**Aldolase**		**pLDH-test_1_**		**pLDH-test_2_**	**pLDH-test_3_**
**N=1237**					**(Now ICT Malaria)**		**(Optimal-IT)**		**(CoreMalaria)**	**(Palutop +4)**
***All species except***	**Pan-antigen:**	Sensitivity			0.57 [0.41-0.74]		0.69 [0.53-0.84]		0.6 [0.44-0.76]	0.63 [0.47-0.79]
***P. falciparum***	***p*LDH**		Specificity		1 [1–1]		1 [0.99-1]		0.99 [0.98-1]	0.99 [0.99-1]
***(P. ovale***,	**or**		PPV		0.99 [0.94-1.0]		0.98 [0.92-1.0]		0.94 [0.86-1.0]	0.96 [0.89-1.0]
***P. malariae,***	**Aldolase**		NPV		0.52 [0.49-0.55]		0.64 [0.61-0.67]		0.55 [0.52-0.58]	0.58 [0.55-0.61]
***or P. vivax)***			LR+		343.4		164.9		60.1 [34.2-106]	94.4 [47.3-189]
					[86.0-1371]		[68.74-395.4]			
			LR-		0.43 [0.29-0.63]		0.32 [0.19-0.51]		0.4 [0.27-0.61]	0.37 [0.24-0.58]
***Plasmodium***	Aldolase*			pLDH-test_1_*		pLDH-test_2_*		pLDH-test_3_*		**Cochran Q test**
***species***	**(Now ICT Malaria)**			**(Optimal-IT)**		**(CoreMalaria)**		**(Palutop +4)**		**P value**
*P. falciparum*	246/334 (73.6%)			271/334 (81.1%)		232/334 (69.5%)		264/334 (78.4%)		<0.0001
*P. ovale*	7/18 (38.9%)			10/18 (55.6%)		8/18 (44.4%)		8/18 (44.4%)		0.10
*P. vivax*	10/11 (90.9%)			10/11 (90.9%)		8/11 (72.8%)		10/11 (90.9%)		0.31
*P. malariae*	5/9 (55.6%)			6/9 (66.7%)		5/9 (55.6%)		6/9 (66.7%)		0.39

* ratio of samples positive in the test to the number of samples positive in the reference method (sensitivity)

## Results

A total of 1311 patients were included in the study (Table 1). Their mean age was 32.7 years (SD: 17.1) and 54% of patients were male. Although 90.2% of patients were living in France, 51.7% originated from malaria-endemic areas. Most patients had returned from Africa (85.0%, n=1060).

**Figure 1:**
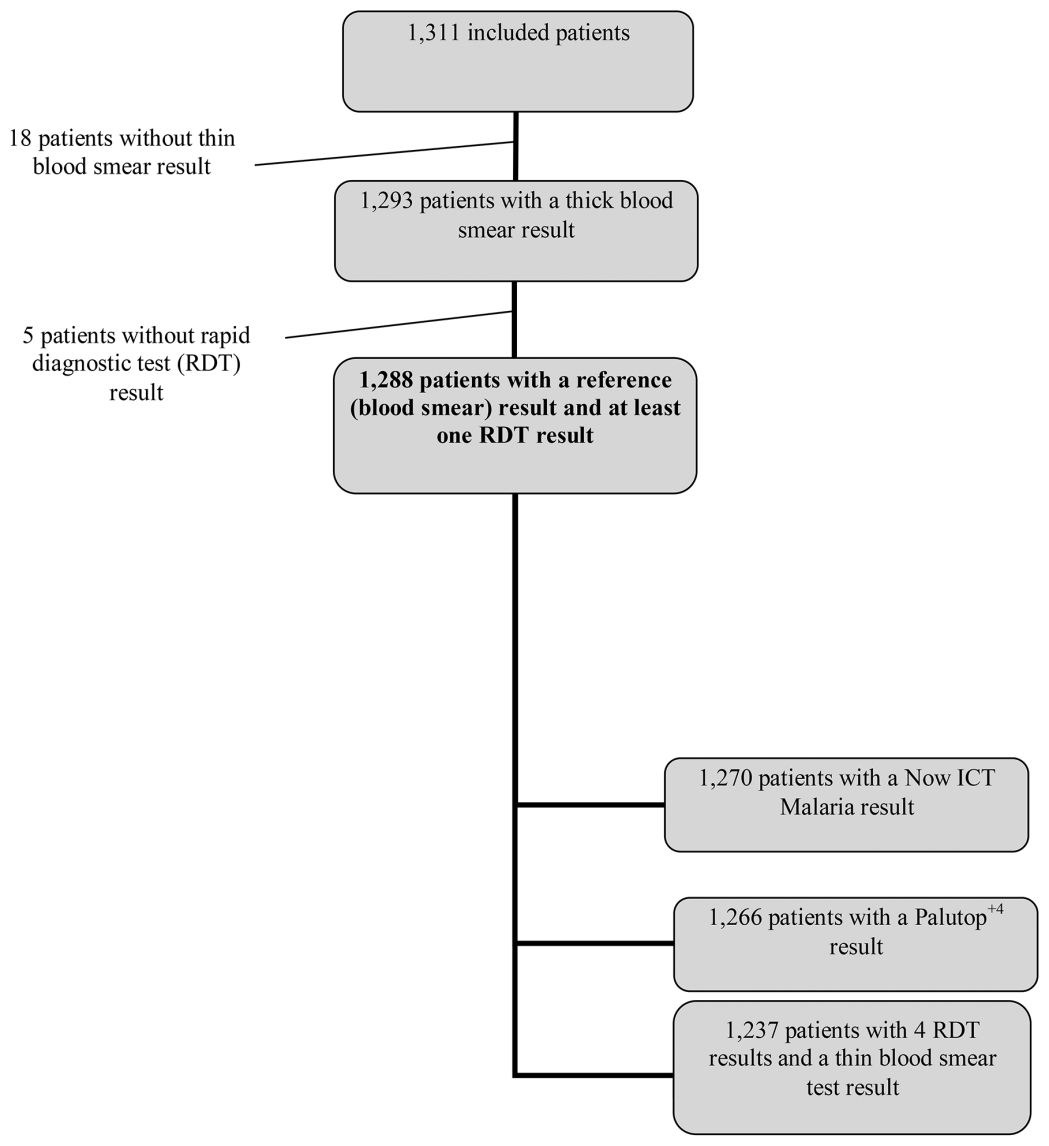
Flow diagram.

Results of the reference thin blood smear test were missing in 18 cases, and RDT results were missing in five cases (Figure 1); thus, 1288 patients were included in the analysis. Malaria was ruled out in 914 cases (71%) including 4 cases in which only *P. falciparum* gametocytes were detected. The reference tests confirmed malaria in 378 cases (28.8%). The species distribution was as follows: *P. falciparum* in 340 cases (89.9%), *P. ovale* in 17 cases (4.5%), *P. vivax* in 11 cases (2.9%), *P. malariae* in 8 cases (2.1%), *P. falciparum* associated with *P. ovale* in 1 case (0.3%), and *P. falciparum* associated with *P. malariae* in 1 case (0.3%). Median parasite density was 11 250/microliter (p/µl; range 8 to 9 000 000 p/µl). Parasite density was above 2000 p/µl in 59% of positive samples (n=221).

**Table 5:** False-positive results obtained with the four RDTs according to the detected antigen and the PCR results.

**Name of the test**		**PfHRP2-test_1_**	**PfHRP2-test_2_**	**PfHRP2-test_3_**	**PfLDH test**		
		**pan-aldolase test**	**pLDH-test_2_**	**pLDH-test_3_**	**pLDH-test_1_**		
		**(Now ICT Malaria)**	**(Core Malaria)**	**(Palutop +4)**	**(Optimal-IT)**		
**False positive RDT results**		**26**	**34**	**29**	**6**		
**Detected antigen**	**PfHRP2 or PfLDH**	26	23	24		4
	**pLDH**		6	2		2
	**PvLDH**		4	1		
	**PvLDH + PfHRP2**		1	1		
	Missing data			1		
**Gametocytes of *P. falciparum***	**Present**	1	2	3		1
	**Absent**	24	30	26		5
	Missing data	1	2			
**PCR result**	***P.falciparum* positive**	12	12	13		4
	**Négative for all species**	13	21	15		2
	Missing data	1	1	1		

The kappa value for thin smear readings was 0.92 (0.76–1).

All the RDT results met the validation criteria. Figure 1 shows the number of results available for each of the four tests.

Considering *P. falciparum*, the sensitivity of the three RDTs targeting PfHRP2 was 96% (Table 3). *P. falciparum* malaria was missed in 8 of 337 cases with PfHRP2-test_3_, 9 of 336 cases with PfHRP2-test_2_, and 10 of 336 cases with PfHRP2-test_1_. However, the specificity of PfHRP2 detection was 97% whatever the RDT. The PPV and NPV of these 3 tests (PfHRP2-test_1_, PfHRP2-test_2_, and PfHRP2-test_3_) were 90% and 98% respectively. The sensitivity of PfHRP2 detection was related to parasite density and depended on the RDT used. Post-hoc analyses showed that sensitivity at parasite densities lower than 200 p/µl was 85% (28/33) with PfHRP2-test_1_, 88% (29/33) with PfHRP_2_-test_2_ and 94% (31/33) with PfHRP_2_-test_3_. Sensitivity at parasite densities between 200 and 2000 p/µl was 91% (48/53) with PfHRP2-test_1_ and PfHRP_2_-test_2_, and 92% (49/53) with PfHRP_2_-test_3_. Sensitivity was 100% with all 3 tests when parasite density was above 2000/µl. Because of the small test-group size, no statistical analysis was possible.

Sensitivity for PfLDH detection was 83%, missing 53 of the 337 cases of *P. falciparum* malaria. Specificity was 100%. The PPV and NPV of this test were 98% and 80% respectively (Table 3). Post-hoc analyses showed that sensitivity was low (41/85, 48.2%) when parasite density was below 2000 p/µl, but increased to 96.7% (238/246) when parasite density exceeded 2000 p/µl.

The RDTs targeting *P. vivax* LDH (PvLDH) had a specificity of 99% and a sensitivity of 82% or 91%, depending on the test (Table 3).

The sensitivity of *P. falciparum* pan-antigen detection was 73.6% for aldolase, and ranged from 69.5% to 81.3% for pLDH, depending on the test (p<0.001) (Table 4).

Regarding non-*falciparum* species, aldolase was detected as a pan-antigen in 57% of microscopically positive samples, whereas the sensitivity of pLDH detection ranged from 60% to 69%, depending on the test (Table 4). Specificity ranged from 99% to 100%, depending on the test (Table 4).

False-positive results are shown in Table 5. Most occurred with PfHRP2, and PCR was usually negative.

False-negative RDT results (relative to microscopy and PCR) are shown in Table 6. Parasitaemia was between 8 p/µl and 117 000p/µl. Except for the PfLDH test/pLDH-test1 (Optimal-IT), most false-negative results involved *P. ovale* or *P. malariae*. Antimalarial drugs were detected in 21.7% to 33.9% of the false-negative samples, with no predominance of a particular drug.

**Table 6:** False-negative RDT results according to the *Plasmodium* species and antimalarial drug assay results.

**Name of the test**		**PfHRP2-test_1_**	**PfHRP2-test_2_**	**PfHRP2-test_3_**	**PfLDH test**
		**pan-aldolase test**	**pLDH-test_2_**	**pLDH-test**	**pLDH-test_1_**
		**(Now ICT Malaria)**	**(CoreMalaria)**	**(Palutop +4)**	**(Optimal-IT)**
**False negative RDT results**		26	23	21	65
**Parasitaemia**	Minimum/Maximum	16/8100	16/8100	16/8100	8/117000
**(p/µl)**	Median [Q1 - Q3]	232.0 [72.0-496.0]	272.0 [72.0-1184.0]	312.0 [120.0-1288.0]	288.0 [68.0-756.0]
**Species**	*P. falciparum*	10 (38.5%)	9 (39.1%)	8 (38.1%)	53 (81.5%)
	*P. ovale*	11 (42.3%)	8 (34.8%)	9 (42.9%)	8 (12.3%)
	*P. vivax*	1 (3.8%)	2 (8.7%)	1 (4.8%)	1 (1.5%)
	*P. malariae*	4 (15.4%)	4 (17.4%)	3 (14.3%)	3 (4.6%)
**Plasmatic**	Négatif	18	16	14	39
**antimalarial**	Positif	5	5	5	20
**detection**	Not done	3	2	2	6

## Discussion

We prospectively evaluated the performance of four malaria RDTs on a large panel of samples (n=1288) obtained from travelers returning from malaria-endemic areas to France.

The overall sensitivity of the RDTs for *Plasmodium* infection (at least one target detected) was higher than 93%, possibly owing to the preponderance of *P. falciparum* in the samples studied, and to the good sensitivity of PfHRP2 detection for *falciparum* malaria, as reported by Abba et al. [10].

The PfHRP2-based tests emerged as a reliable alternative to routine microscopy for the diagnosis of *P. falciparum* malaria, and were more sensitive than the PfLDH-based tests. Taking microscopy as the standard, the sensitivity of the PfHRP2-based tests was 96% (95% CI, 94%-98%), which is higher than the threshold of 95% recommended by the World Health Organization (WHO) [29].

More than 60 RDT brands and 200 different products have already been developed. WHO and the Foundation for Innovative New Diagnostics (FIND) evaluated the sensitivity of 168 RDTs for the diagnosis of *P. falciparum* and *P. vivax* malaria [18]. This four-round evaluation showed that the tests submitted to Round 4 performed better, possibly reflecting improvements in test manufacture. The proportion of tests achieving a panel detection rate higher than 75% for a parasite density of >200/µl was higher in the latter study than previously reported. Performance varied widely among the tests at low parasite densities (below 200/µl), but the majority of tests gave high detection rates at densities of 2000 or 5000 parasites/μl [18], as also observed in our study. Only 3 among the 4 tests studied here were part of the WHO panel, namely Binax Now Malaria (PfHRP2-test_1_, pan-aldolase test) with performance approximately 87% at 200 parasites/µl and 98% at 2000 p/μl); Core Malaria Pan/Pv/Pf (PfHRP2-test_2_, pLDH-test_2_), respectively 89% and 98%; and Optimal iT (PfLDH-test, pLDH-test_1_), 50% at 200 p/µl and 98% at 2000 p/μl.

In areas where transmission rates are low, parasite density is likely to be lower in patients with symptomatic malaria [9,30]. Thus, test performance at a parasite density of 200/µl is an important criterion. Imported malaria is defined as the presence of *Plasmodium* *sp.* in blood, whatever the parasite density. This requires a highly sensitive diagnostic test, as false negativity may lead to a life-threatening delay in treatment. In non-endemic areas, RDTs are mainly useful for confirming infection suggested by low microscopic parasite density, and for determining the species. Nonetheless, despite an NPV of 98% and a sensitivity of 96% obtained here with the PfHRP2-based tests, a negative RDT result does not rule out imported *P. falciparum* malaria [31]. Of note, all but one of the false-negative PfHRP2 test results in our study involved samples with parasite densities below 2000/µl.

The specificity and NPV of the pLDH-based tests were both 100%. Specificity higher than 90% has been reported in endemic areas [14]. In contrast, the sensitivity of the pLDH-based test was only 83% (95% CI, 79%–87%) in our study, while previous reports have shown sensitivities ranging from 85.1% to 99% among travelers [17,32] and from 85.6% to 98% in endemic countries [12,13], values lower than those of the PfHPR2-based tests for *P. falciparum* malaria, especially when parasite density was low (<2000/µl) [18].

Thus, RDT performance relies on the choice of monoclonal antibodies [33]. Better sensitivity would allow malaria to be ruled out by a negative RDT result, thus avoiding inappropriate presumptive treatment in areas where this therapeutic strategy is applied [17,34-36].

Although severe malaria, including imported malaria, is almost always due to *P. falciparum* (and occasionally *P. vivax* or *P* *. knowlesi* , depending on the country visited) [37], species identification can guide the choice of treatment and avoid costly investigations. In our study, 11% of cases were due to non-*falciparum* species (*P. ovale*, *P. vivax* and *P. malariae*), and no cases of *P* *. knowlesi* infection were diagnosed.

The RDTs tested here were unable to identify non-*falciparum* species, especially *P. ovale* and *P. malariae*, as previously reported [38-40]. However, the sensitivity of the PvLDH-based tests was 91% (95% CI, 74%–100%) for *P. vivax*, especially with the PfHRP2 test_3_ – pLDH test_3_ (Palutop 4+) assay. Few PvLDH-based RDTs are available, and they have been evaluated in only a handful of studies. Meena et al. reported 76.6% sensitivity and 98.1% specificity for the FalciVax test (Orchid) [41]. Another study reported higher sensitivity (93.4%) for the SD Malaria Antigen P.v. test [39]. WHO reported a wide range of sensitivities for *P. vivax* detection with specific PvLDH RDTs, ranging from 5.9% to 100% (94.3% for the Core Malaria Pan/Pv/Pf) at higher parasite densities (2000/µl) [18].

False-positive results, representing 3% in our study, may have several causes. Nearly half (12/26) of the samples with positive RDT results and negative blood smears corresponded to PCR-confirmed *P. falciparum* malaria; these patients received no specific monitoring but were treated with antimalarial drugs in case of high suspicion of malaria. These samples may have been from patients with a suspected relapse of malaria, in which case their false positivity would indicate the persistence of PfHRP2 antigen in the bloodstream after treatment [42]; alternatively, they would confirm that RDTs can diagnose malaria attacks earlier than microscopy. We were unable to test these hypotheses. Cross-reactivity due to self-antibodies such as rheumatoid factor, especially in RDTs in which the conjugate is an IgM antibody [43], is also possible, as the manufacturers of the tests studied here do not specify the conjugate isotypes, and we were unable to screen the samples for rheumatoid factor or HAMA (human antimouse antibodies). A possible impact of gametocytes on RDT specificity [44,45] could not be excluded either, as too few samples contained gametocytes and no asexual forms to draw firm conclusion.

The choice of blood smears as the reference diagnostic test may represent a limitation of our study, as PCR has been proposed as the gold standard [46]. However, clearance of plasmodium DNA from the bloodstream following antimalarial treatment should be further studied to support and validate this option [47].

False-negative results for *P. falciparum* were observed mainly with the PfLDH-based test. Some of these samples were from patients treated with antimalarial drugs before their inclusion in the study, as shown by plasma drug assays; however, these treatments were not declared by the patients concerned, and their timing could not be determined. Dead parasites were observed on microscopy, but PfLDH production could have been halted by therapy [5,26], possibly contributing to the good specificity of the pLDH test [48].

The low sensitivity of RDT tests may also related to low parasite density, which could also explain the false-negative results obtained with the PfHRP2 detection tests, although some parasites isolated in South America and Africa have been shown not to produce PfHRP2 [49,50]. In such cases PfLDH detection would be of interest [51].

In conclusion, the three PfHRP2-based RDTs tested here showed high sensitivity and acceptable specificity for the diagnosis of imported and uncomplicated *P. falciparum* malaria, and thus appear to be a reliable, rapid and simple first-line diagnostic option for this potentially life-threatening disease, particularly in emergency settings. However, RDTs alone cannot rule out malaria, meaning that negative results must be confirmed by microscopy or PCR, and patients must be kept under medical supervision until the result is obtained [52,53].

## Supporting Information

STARD checklist S1 **STARD checklist for reporting of studies of diagnostic accuracy**
(DOC)

ANRS recommendation research study S1 **Recommendations about obligations for researchers to the patient (in French).**
(PDF)

Report of ethic committee S1 **Acceptance of the project by the Ethic Committee (in French).**
(PDF)

Study protocol S1 **Detailed protocol of the research (in French).**
(PDF)

Protocol summary S1 **Summary of the research protocol.**
(DOC)
