# Supplementary material for: Performance of Rapid Diagnostic Tests for Imported Malaria in Clinical Practice: Results of a National Multicenter Study
Source: PLoS One. 2013 Sep 30;8(9):e75486. doi: 10.1371/journal.pone.0075486 (PMC3787089; doi:10.1371/journal.pone.0075486)
Supplement: ANRS recommendation research study S1 — Recommendations about obligations for researchers to the patient (in French). (PDF) [file pone.0075486.s002.pdf]

Agence nationale de recherches sur le sida et les hépatites virales

TYPOLOGIE DE LA RECHERCHE SUR LA PERSONNE

La réglementation française applicable à la recherche sur la personne n’est pas la même selon la typologie de la recherche. On distingue 3 catégories de recherches : Recherche Biomédicale, Recherche en soin courant ou Recherches non interventionnelles avec ou sans prélèvements biologiques (réunis en collection).
La dichotomie se base sur l’aspect interventionnelle ou non interventionnelle de la recherche :
- Dans les recherches interventionnelles, l’intervention est définie comme étant liée à la recherche et non au soin (cas de recherches biomédicales et soins courants) ;
- Dans les recherches non interventionnelles, il n’y a aucune interférence avec le soin (cas des recherches uniquement sur prélèvements biologiques).

Vous trouverez ci-après un synopsis indiquant la catégorie applicable à votre recherche et
le numéro de la fiche explicative associée :

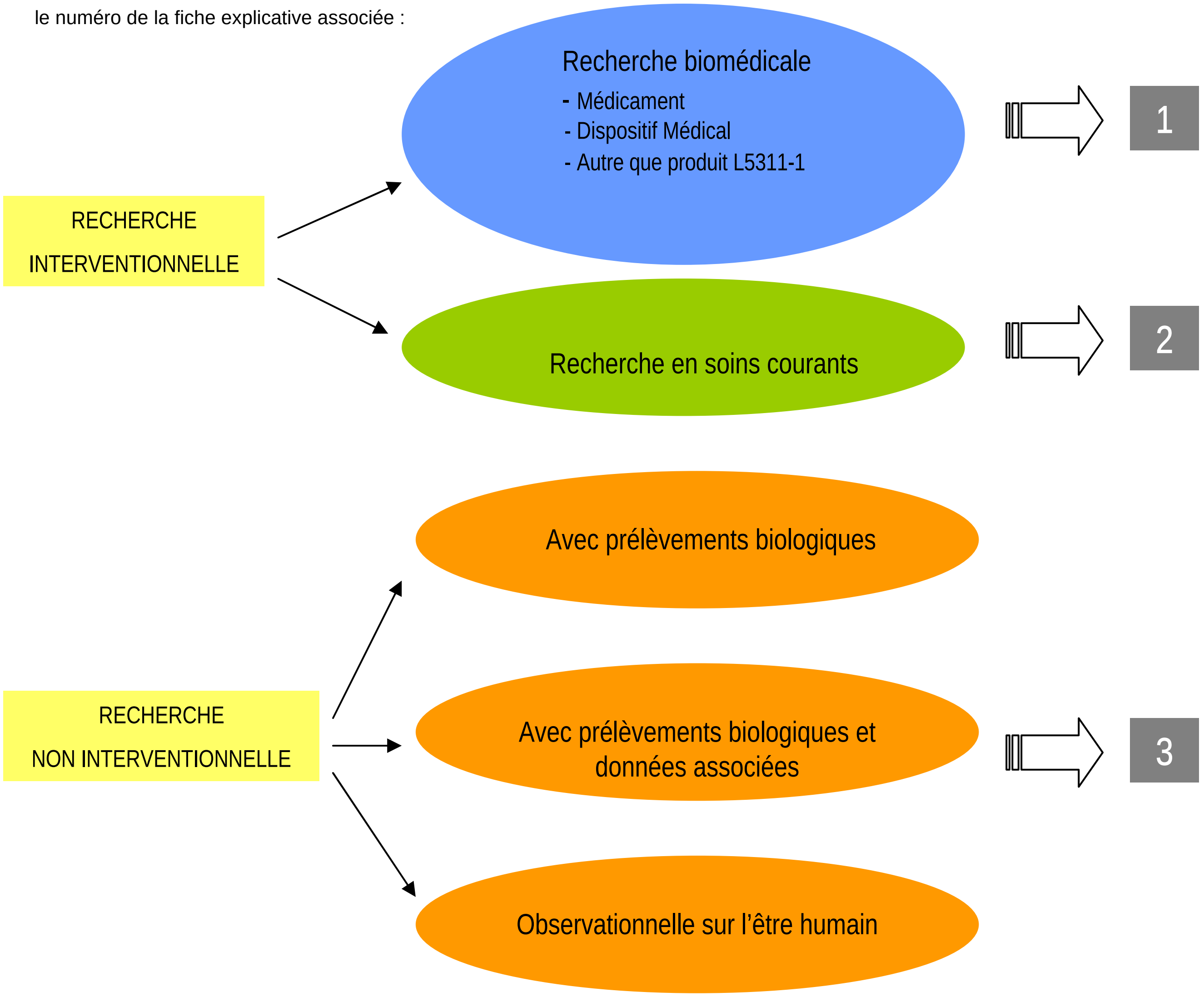

anRS
Agence nationale de recherches sur le sida et les hépatites virales

Recherche Clinique Interventionnelle
Recherche Biomédicale - promotion ANRS[1]

1

Il s'agit d'une Recherche organisée et pratiquée sur la personne en vue du développement des connaissances biologiques ou médicales. Elle porte notamment sur les médicaments, les dispositifs médicaux ou les non produits de santé (art. L.5311-1 du CSP). **Schématiquement**, rentrent dans ce cadre toutes les recherches qui impliquent une action sur la personne, malade ou non, et qui en modifie la prise en charge (administration de médicaments, implantation d'un dispositif, investigation physiologique, …) sauf si cette intervention ressort de la pratique courante.

Les intervenants sont : le promoteur ANRS - les CMG mandatés par le promoteur - les investigateurs (médecins inscrits au l'Ordre des Médecins) dont l'investigateur coordonnateur - les personnes participant à la recherche (volontaires sains ou patients) qui doivent être affiliées au régime de sécurité sociale ou bénéficier d'un tel régime (CMU, …).

Les documents importants sont : le protocole de recherche [2, 3, 4], la note d'information et le consentement [1] des personnes participant à la recherche, le résumé des caractéristiques du produit et/ou la brochure pour l'investigateur [5], éventuellement les modifications substantielles[6, 7, 8], et le rapport clinique[9,10,11] en fin de recherche.

Le suivi de l'essai est assuré par un conseil scientifique, un comité indépendant et du monitorage [12]

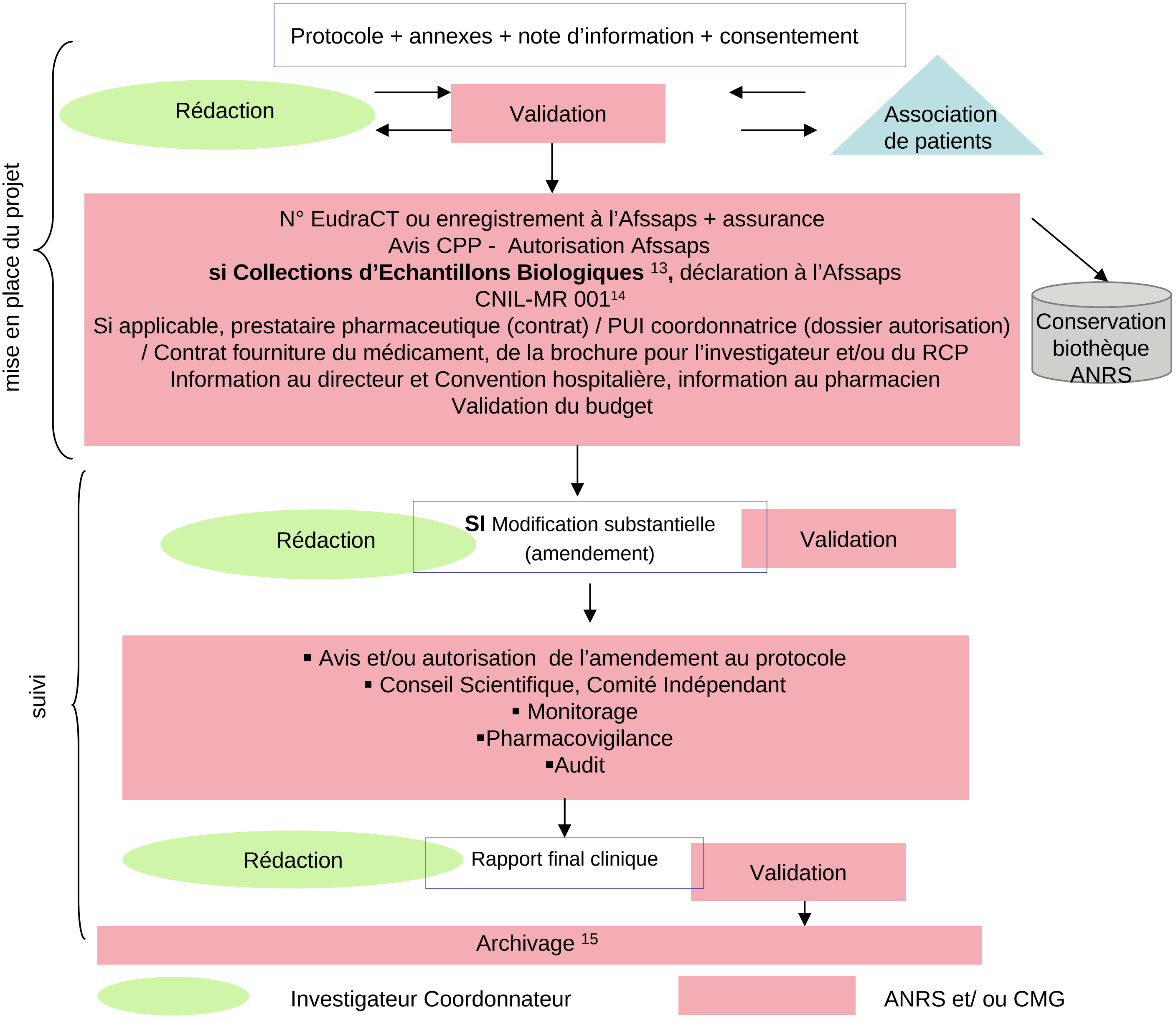

Recherche Clinique Interventionnelle Soins Courants – Sous l'égide de l'ANRS[16]

Il s'agit des recherches comportant une intervention (par ex, une randomisation, ..) **:**

- visant à évaluer les soins courants,
- **autres que celles portant sur les médicaments**,
- **uniquement** lorsque tous les actes sont pratiqués et les produits utilisés de manière habituelle.
(cf. : Annexe I – soins courants)

NB : la simple prise de sang n'est pas considérée comme une intervention
Les intervenants sont : la personne physique ou morale qui prend l'initiative de ces recherches, en assure la gestion et vérifie que son financement est prévu, et les patients. L'ANRS peut, le cas échéant, prendre la responsabilité réglementaire de la recherche.

Les documents importants sont : le projet de recherche, la note d'information aux patients.
Le suivi de l'essai est assuré par un comité de surveillance indépendant, un conseil scientifique auquel est associé l'ANRS, et un contrôle et assurance de la qualité

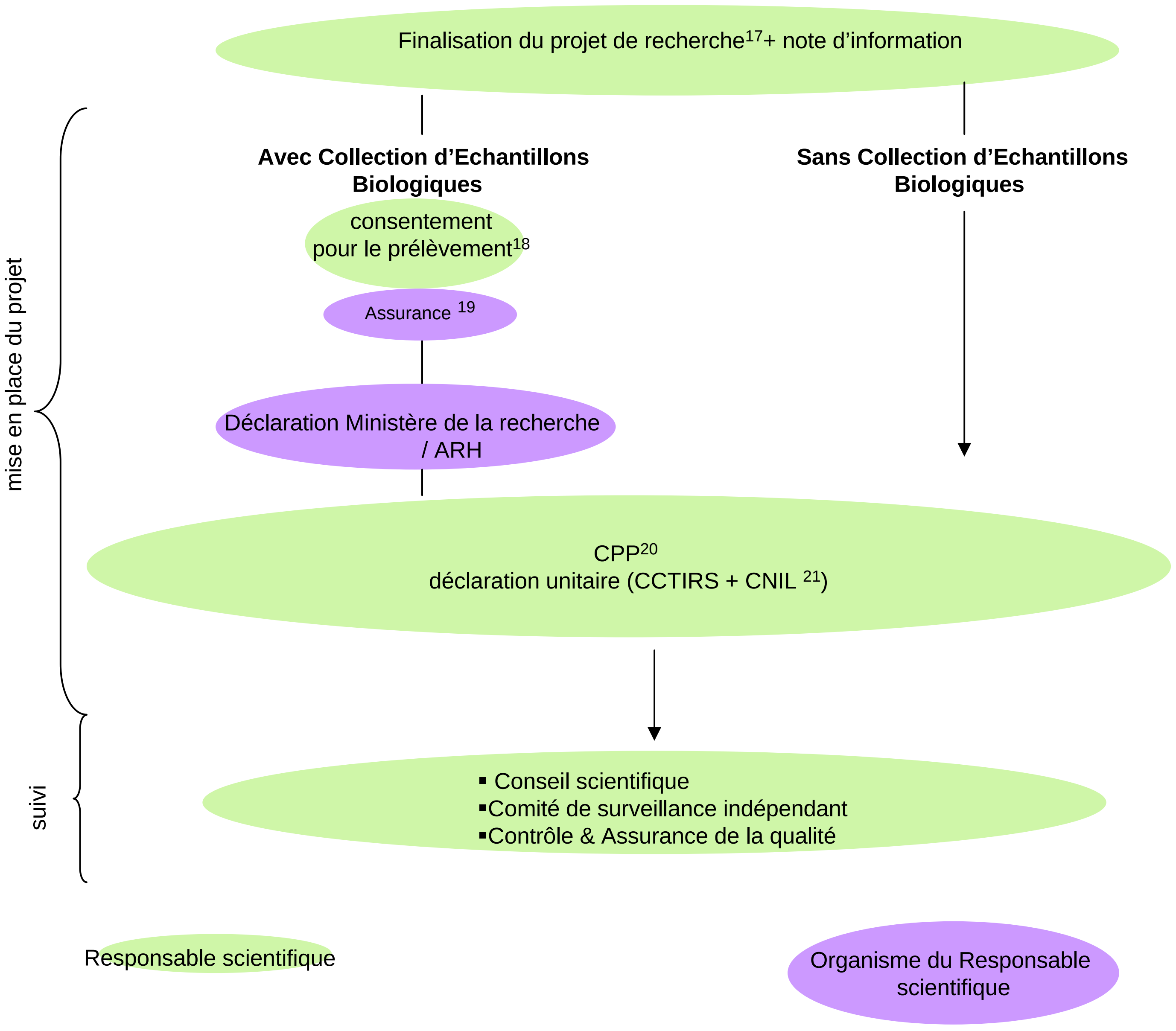

ANNEXE I

Recherche Soins courants

La notion de recherche visant à évaluer les soins courants n'est pas strictement définie par les textes. Toutefois, l'arrêté comprend en annexe un document que tout demandeur devra utiliser afin de permettre au CPP de s'assurer que le projet soumis porte effectivement sur l'évaluation des soins courants. Ce document permet, du fait de la liste des éléments à fournir, de mieux comprendre ce qui pourra constituer une telle recherche.

En particulier :

- les stratégies médicales objets de la recherche au regard de la population concernée devront être utilisées de manière habituelle ;

- il devra exister un consensus professionnel relatif aux stratégies médicales faisant l'objet de la recherche (ce n'est pas une stratégie innovante ni à contrario obsolète) ;

- lorsque la recherche porte sur une combinaison de stratégies médicales, aucune des stratégies soumises à la comparaison devra, en l'état des connaissances, être considérée comme étant supérieure à l'autre en termes de sécurité et d'efficacité ;

- les produits utilisés le cas échéant au cours de la recherche, qui ne peuvent pas être des médicaments doivent être utilisés dans des conditions d'utilisation conformes à leur destination et leur utilisation en pratique courante ;

- les actes pratiqués et les méthodes utilisées doivent être conformes à la pratique courante ;

- les modalités particulières de surveillance ne doivent comporter que des risques et des contraintes négligeables et doivent être décrites dans un protocole.

anRS
Agence nationale de recherches sur le sida et les hépatites virales

3

RECHERCHE NON INTERVENTIONNELLE
avec ou sans collection biologique

Les recherches dans lesquelles un scientifique ne fait qu’observer une population, un phénomène sans intervenir en quoi que ce soit sur le cours naturel des choses sont soumises à la réglementation des **recherches non interventionnelles**.
Elles correspondent à des recherches dans lesquelles tous les actes sont pratiqués et les produits utilisés de manière habituelle, sans aucune procédure supplémentaire ou inhabituelle de diagnostic ou de surveillance (articles L.1121-1 alinéa 1 et R1121-2 du CSP).

L’utilisation d’éléments biologiques à des fins différentes de celles qui ont justifié le prélèvement et dans un contexte de recherche non interventionnelle, **est,** depuis la loi n° 2004-800 du 6 août 2004 et la publication de son décret en août 2007, **soumis à de nouvelles dispositions réglementaires**.
Désormais, **une simple prise de sang destinée à la recherche, même pratiquée en dehors du suivi du patient, n’est pas considérée comme une procédure supplémentaire**.
Ces recherches sont donc considérées comme des recherches non interventionnelles avec collection biologique avec ou sans données associées.
Elles ne relèvent pas du dispositif applicable aux recherches biomédicales (cf fiche 1).
Il n’y a pas de promotion par l’ANRS pour ce type de recherche.

A partir de ce constat, trois cadres réglementaires se distinguent :

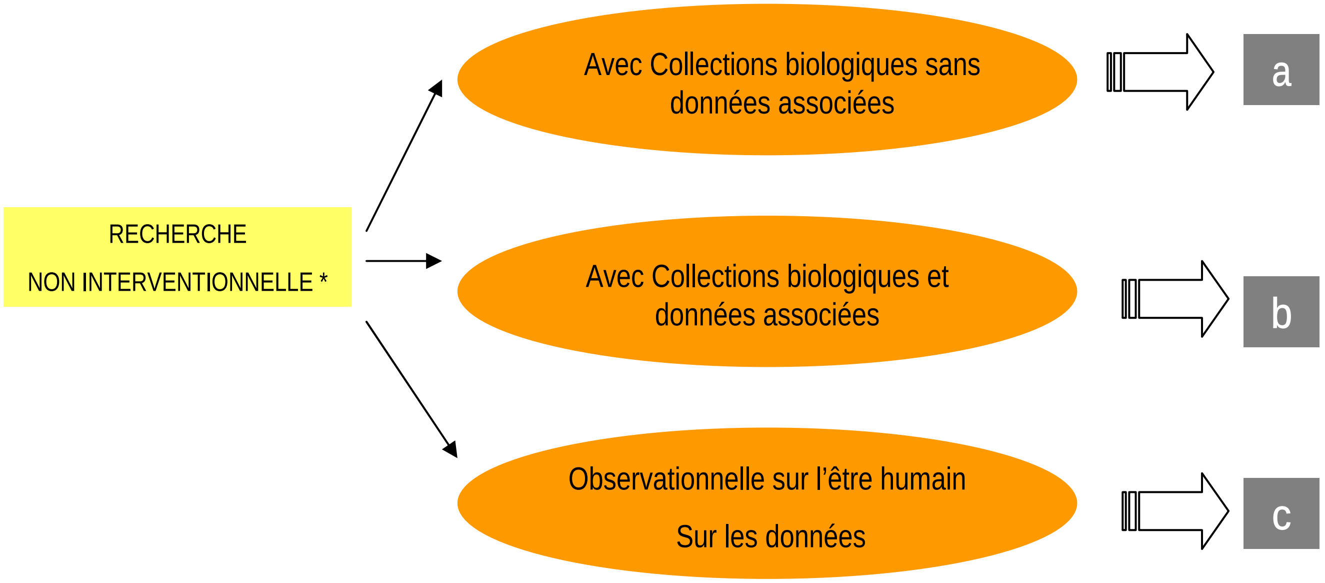

* Non Interventionnelle = aucune interférence avec le soin. Par opposition aux recherches interventionnelles où l’intervention est définie comme étant liée à la recherche et non au soin.
Les intervenants sont : l’organisateur de la recherche (responsable qui initie les démarches réglementaires), les personnes qui se prêtent à la recherche, l’organisme dont dépend le chercheur (par ex URC pour un chercheur APHP) et le financeur ANRS.

anRS
Agence nationale de recherches sur le sida et les hépatites virales

Recherche biologique ou médicale non-interventionnelle avec collection biologique sans données associées ou totalement anonymes

a

Une collection d'éléments biologiques humains désignent la réunion, à des fins scientifiques, de prélèvements biologiques effectués sur un groupe de personnes identifiées et sélectionnées en fonction des caractéristiques cliniques ou biologiques d'un ou plusieurs membres du groupe, ainsi que les dérivés de ces prélèvements.
Une collection de ressources biologiques humaines est soumise, par la loi à déclaration (cf. complément d'information en page 9).
La notion de collection est prise au sens large : plusieurs prélèvements dans le temps sont considérés comme une collection.
Ce type de recherche s'applique aussi aux prélèvements obtenus auprès de volontaire.

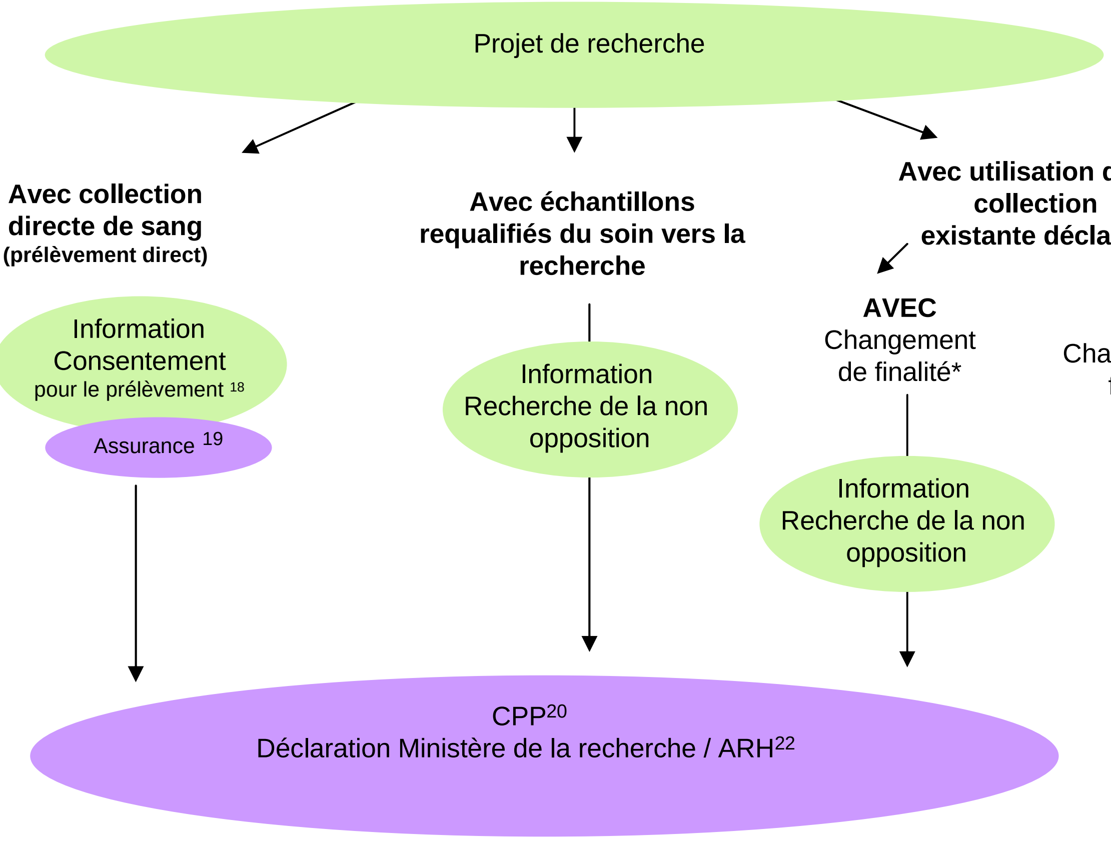

** avec changement de finalité : utilisation des échantillons non-conforme à la déclaration au ministère de la recherche : ex programmes de recherche différents ou changement de lieu de conservation de la collection*
*** sans changement de finalité : conforme à la déclaration au ministère de la recherche et à l'indication initiale spécifiée lors du consentement ou du recueil de la non opposition*

Responsable scientifique

Organisme du Responsable scientifique

Recherche biologique ou médicale non-interventionnelle avec collection biologique

Et données associées anonymisées

b

Lorsque, des fichiers informatisés sont associés à des recherches avec collection biologique et qu'ils contiennent des données personnelles sur les personnes dont sont issues les prélèvements, les dispositions de la Loi Informatique et Libertés s'appliquent. Ces dispositions sont indépendantes de celles de la déclaration des collections biologiques.
La notion de donnée personnelle est définie comme étant « toute information relative à une personne physique identifiée ou qui peut être identifiée, directement ou indirectement, par un numéro d'identification ou à un ou plusieurs éléments qui lui sont propres » (cf complément d'information en page 10).

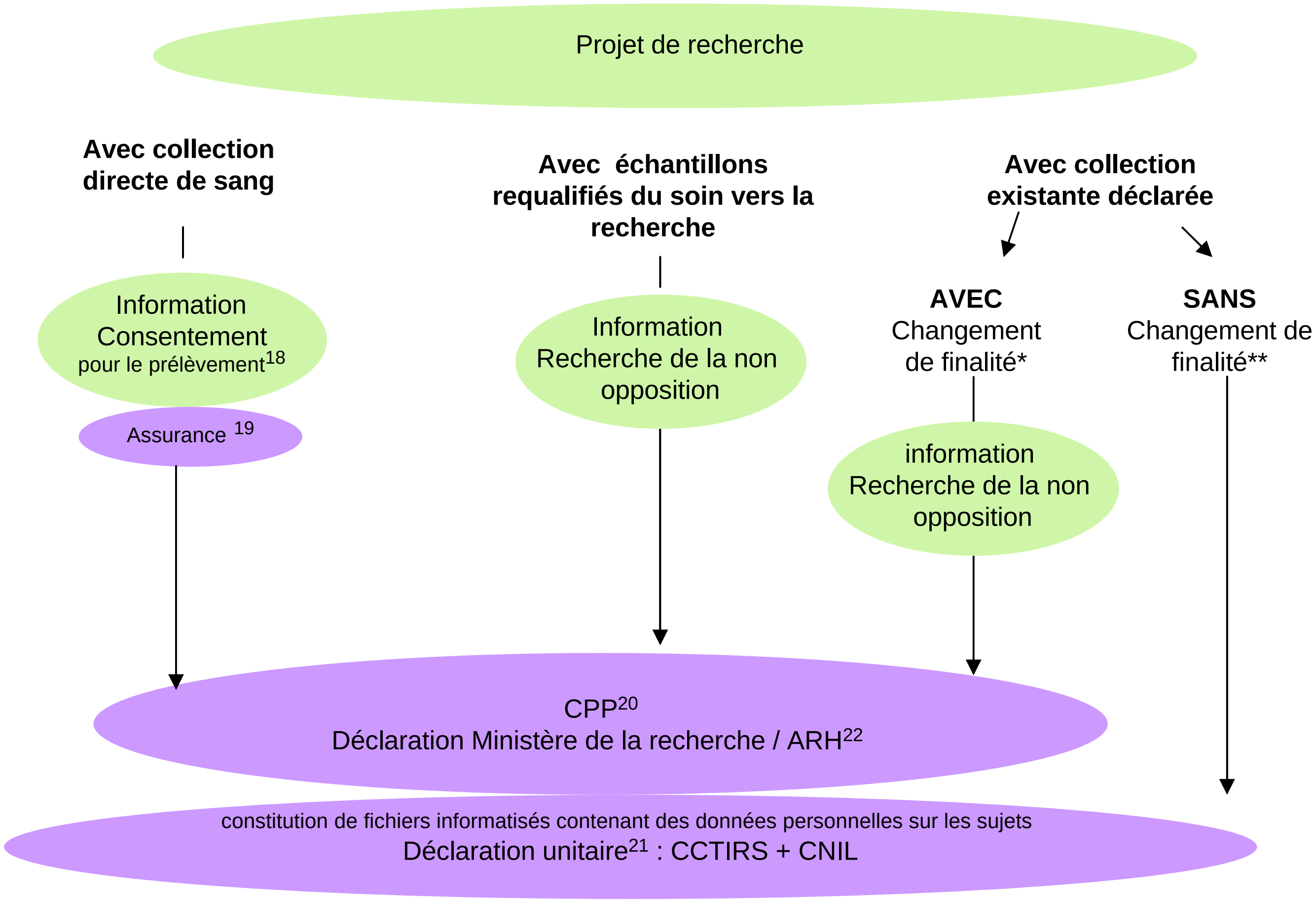

** avec changement de finalité : utilisation des échantillons non-conforme à la déclaration au ministère de la recherche : ex programmes de recherche différents ou changement de lieu de conservation de la collection*
*** sans changement de finalité : conforme à la déclaration au ministère de la recherche et à l'indication initiale spécifiée lors du consentement ou du recueil de la non opposition*

Responsable scientifique

Organisme du Responsable scientifique

Recherche
non-interventionnelle
Sans prélèvement biologique

C

Les recherches non interventionnelles traitant des données à caractère personnel ayant pour fin la recherche dans le domaine de la santé sont soumises à la Loi Informatique et Libertés, en particulier au chapitre IX.
La notion de donnée personnelle est définie comme étant « toute information relative à une personne physique identifiée ou qui peut être identifiée, directement ou indirectement, par un numéro d'identification ou à un ou plusieurs éléments qui lui sont propres (cf. complément d'information en page 10).
La plupart des revues internationales exigent de plus en plus l'avis d'un comité éthique de la recherche (en référence à la déclaration d'Helsinki) pour publier.

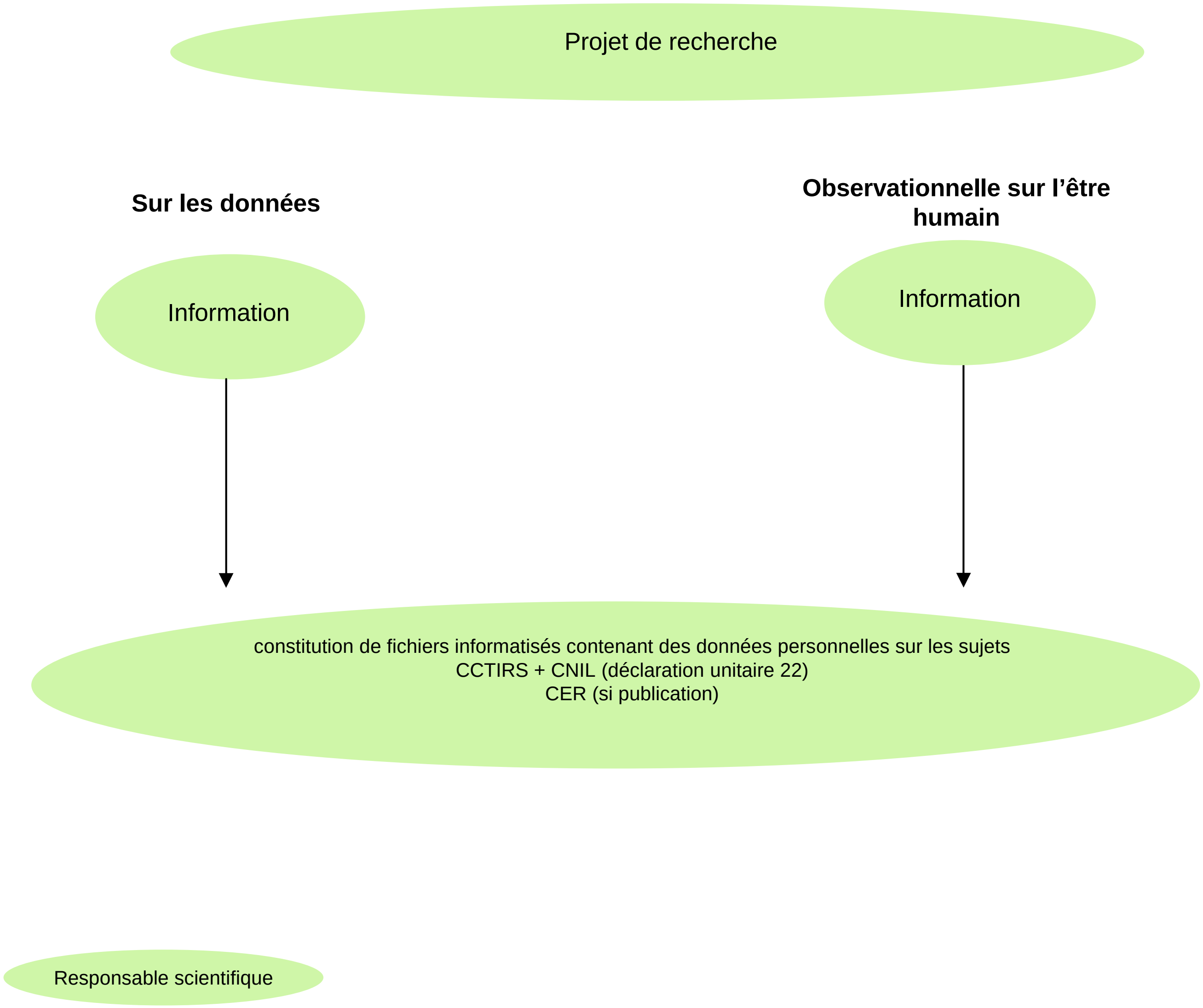

Complément d'information
Sur les échantillons biologiques

Les dispositions légales et réglementaires sont récentes ; des modifications législatives devraient probablement apporter des précisions …

On appelle collection biologique l'ensemble des échantillons issus du corps humain provenant de plusieurs personnes participant à une même étude.

Depuis la loi de bioéthique d'août 2004, elles sont soumises à des dispositions réglementaires :

- Dans le cas d'une constitution directe de sang : Les prélèvements effectués ne doivent comporter que des risques négligeables. Il incombe pour l'établissement du responsable de la recherche une obligation d'assurance conforme à l'art. L 1121-10 du CSP. Obligation d'information préalable du sujet ; nécessité d'une absence d'opposition ; possibilité d'une utilisation ultérieure distincte des prélèvements , sans recueil nécessaire d'une nouvelle absence d'opposition, si la recherche se situe dans le même champ d'investigation

- Les démarches d'autorisation ou de déclaration de la collection doivent être effectuées par la personne morale, c'est-à-dire par l'établissement ou l'organisme qui assure la conservation de la collection.

- L'avis au Comité de Protection des Personnes sollicité est celui de la région dont dépend le responsable de la recherche. Cette demande d'avis est conditionnée au paiement d'une taxe auprès de l'Afssaps, qui nécessite au préalable un enregistrement de la recherche auprès de l'Assaps.
- Le CPP est sollicité en cas de constitution d'une collection d'échantillons biologiques et, en cas d'utilisation d'éléments et de produits du corps humains à des fins scientifiques relevant d'un changement de finalité par rapport au consentement initialement.

Lorsqu'un examen est effectué en vue d'un examen des caractéristiques génétiques de la personne, la loi requiert un consentement écrit de la personne , qui doit être dûment informée, préalablement, de la nature et de la finalité de l'utilisation des échantillons.

Pour les projets associant chercheurs fondamentaux et cliniciens sur des prélèvements humains, l'ANRS conseille que l'organisateur de la recherche soit le clinicien.
Dans le cas d'un projet comportant plusieurs référents, un référent coordonnateur doit être désigné.
Ainsi, l'organisme en charge des démarches sera celui du coordonnateur.

Adresses utiles
AP-HP :
http://www.drrc.ap-hop-paris.fr/ressources_biologiques/accueil_rb.php
http://web91.ap-hop-paris.fr/ressources_biologiques/document_types_rb.php

Guide pratique sur « Recueillir, conserver et utiliser des échantillons biologiques humains à l'hôpital » édité par l'APHP précise les principes et les régles qui s'imposent aujourd'hui pour les activités de collections biologique :
http://www.drrc.aphp.fr/ressources_biologiques/doc_rb/Vademecum_Juridique_V20.pdf

Ministère de l'enseignement supérieur et de la recherche :
http://appliweb.dgri.education.fr/appli_web/codecoh/IdentCodec.jsp
Afssaps :
http://afssaps.sante.fr/htm/5/essclin/indesscl.htm
Paiement de la taxe :
http://afssaps.sante.fr/htm/3/rcb/rcb.htm
CPP (liste des 40 comités) :
http://www.recherchebiomedicale.sante.gouv.fr/pro/comites/coordonnees.htm

Complément d'information
Les traitements informatisés des données

Lorsque des fichiers informatisés sont constitués dans les projets de recherche, les dispositions de la Loi Informatique et Liberté s'appliquent :

- Les personnes auprès desquelles sont recueillies les données à caractère personnel doivent être individuellement informées ;
- Dans le cas des traitements de données à caractères personnel ayant pour fin la recherche dans le domaine de la santé, chaque responsable de traitement de données doit faire une déclaration unitaire pour chaque recherche. Il n'est pas possible de débuter la recherche et le recueil des données avant l'obtention de l'autorisation de la CNIL. Par exemple, lorsque le responsable du fichier exerce au sein de l'AP-HP, l'organisme déclarant est l'hôpital.

La procédure de la déclaration unitaire se déroule en deux temps :

- Demande d'avis auprès du Comité consultatif sur le traitement de l'information en matière de recherche dans le domaine de la santé (CCTIRS). Ce comité doit rendre son avis dans un délai de 1 mois.
- Demande d'autorisation auprès de la CNIL : la commission dispose d'un délai de 2 mois renouvelable une fois pour notifier son autorisation.

Adresses utiles :

CCTIRS :
http://www.enseignementsup-recherche.gouv.fr/cid20537/cctirs.html

CNIL :
Loi du 6 janvier 1978 modifiée relative à l'informatique, aux fichiers et aux libertés :
http://www.cnil.fr/index.php?id=301#CHAPITRE9
Mode d'emploi :
http://www.cnil.fr/index.php?id=33

ANNEXE II

Référence

1 : Code de la santé publique : Partie législative telle que modifiée notamment par la loi relative à la politique de santé publique n°2004-806 du 9 août 2004 (titre 2: recherche biomédicale) & Partie réglementaire telle que modifiée par le décret relatif aux recherches biomédicales n° 806-477 du 26 avril 2006 et textes subséquents

2 : Arrêté du 24 mai 2006 relatif au contenu et modalités de présentation d'un protocole de recherche biomédicale portant sur **un médicament** à usage humain

3 : Arrêté du 16 août 2006 relatif au contenu et modalités de présentation d'un protocole de recherche biomédicale portant **sur un dispositif médical** ou sur un dispositif médical de diagnostic *in vitro*

4 : Arrêté du 16 août 2006 relatif au contenu et modalités de présentation d'un protocole de recherche biomédicale **ne portant pas sur un produit mentionné à l'Art. L-5311-1 du CSP** (Non produit de santé)

5 : Arrêté du 19 mai 2006 relatif au contenu et modalités de présentation d'une brochure pour l'investigateur d'une recherche biomédicale portant sur **un médicament** à usage humain

6 : Arrêté du 4 septembre 2006 modifiant l'arrêté du 19 mai 2006 fixant les modalités de présentation et le contenu de la demande de modification substantielle d'une recherche biomédicale portant sur **un médicament** à usage humain auprès de l'Agence française de sécurité sanitaire des produits de santé et du comité de protection des personnes

7 : Arrêté du 24 août 2006 fixant les modalités de présentation et le contenu de la demande de modification substantielle d'une recherche biomédicale portant **sur un dispositif médical** ou sur un dispositif médical de diagnostic *in vitro* auprès de l'Agence française de sécurité sanitaire des produits de santé et du comité de protection des personnes

8 : Arrêté du 11 septembre 2006 fixant les modalités de présentation et le contenu de la demande de modification substantielle d'une recherche biomédicale **ne portant pas sur un produit mentionné à l'Art. L-5311-1 du CSP** (Non produit de santé) auprès du ministre chargé de la santé et du comité de protection des personnes

9 : Arrêté du 19 mai 2006 relatif au contenu et aux modalités de présentation des informations relatives à la fin de la recherche, au rapport final d'une recherche biomédicale portant sur **un médicament** à usage humain ; normes ICH E3 – CPMP/ICH/137/95 (step4, 30/11/95) :Structure and content of clinical study reports

10 : Arrêté du 25 août 2006 fixant le contenu et les modalités de présentation des informations relatives à la fin de la recherche, au rapport final et au résumé du rapport final d'une recherche biomédicale **sur un dispositif médical** ou sur un dispositif médical de diagnostic *in vitro*

11 : Arrêté du 23 octobre 2006 relatif au contenu et aux modalités de présentation des informations relatives à la fin de recherche, au rapport final et au résumé du rapport final d'une recherche biomédicale ne portant pas sur un produit mentionné à l'Art. L-5311-1 du CSP

12 : Décision du 24 nov. 2006 fixant les règles des BPC pour les recherches biomédicales portant sur les médicaments à usage humain

13 : Article L.1243-3 (2°) du CSP

14 : Loi « Informatique et Libertés » du 6 janvier 1978 modifiée notamment par la loi n°2004-801 du 6 août 2004 et Décision du 5 janvier 2006 portant homologation d'une méthodologie de référence pour les traitements de données personnelles opérés dans le cadre des recherches biomédicales (méthodologie de référence MR-001)

15 : Arrêté du 8 novembre 2006 fixant la durée de conservation par le promoteur et l'investigateur des documents et données relatifs à une recherche biomédicale portant sur un médicament à usage humain

16 : Art L. 1121-1 (2°) du code de la santé publique ; Art R1121-3 du décret n° 2006-477 du 26 avril 2006 ; Arrêté du 9 mars 2007 fixant la liste des produits mentionnés à l'article L. 1121-1 (2°) du code de la santé publique

17 : Arrêté du 9 mars 2007 fixant la composition du dossier de demande d'avis au CPP pour les recherches visant à évaluer les soins courants mentionnées au 2° alinéa de l'article L.1121-1 du CSP

18 : Art. L 1221-3 du CSP

19 : Art. L 1221-8-1 du CSP

20 : Arrêté du 25 août 2005 fixant le barème et les modalités de recouvrement de la taxe et de la taxe additionnelle prévues à l'article L. 1123-8 du CSP

21 : Loi « Informatique et Libertés » du 6 janvier 1978 modifiée notamment par la loi n°2004-801 du 6 août 2004, chapitre IX

22 : Arrêté du 16 août 2007 fixant le modèle de dossier accompagnant les déclarations et les demandes d'autorisation de conservation et de préparation à des fins scientifiques d'éléments du corps humain

ANNEXE III

Glossaire

Afssaps : Agence française de sécurité sanitaire des produits de santé
ARH : Agence Régionale d'Hospitalisation
BPC : Bonnes pratiques cliniques
CCTIRS : Comité Consultatif sur le Traitement de l'Information en matière de Recherche dans le domaine de la Santé
CEB : Collection d'Échantillons Biologiques
CER : Comité d'Éthique pour la Recherche
CNIL : Commission Nationale de l'Informatique et des Libertés
CMG : Centre de Méthodologie et de Gestion
CPP : Comité de Protection des Personnes
CSP : Code de la Santé Publique
DGS : Direction Générale de la Santé
PUI : Pharmacie à usage intérieur

Liens utiles :

Afssaps :
http://afssaps.sante.fr/htm/5/essclin/indesscl.htm

CCTIRS :
http://www.enseignementsup-recherche.gouv.fr/cid20537/cctirs.html

CPP (liste des 40 comités) :
http://www.recherchebiomedicale.sante.gouv.fr/pro/comites/coordonnees.htm

CNIL :
http://www.cnil.fr/index.php?id=1923&print=1

Direction Générale de la Santé :
http://www.recherchebiomedicale.sante.gouv.fr

Légifrance (textes réglementaires – Code de la Santé Publique) :
http://www.legifrance.gouv.fr/WAspad/ListeCodes
