# Supplementary material for: Performance of Rapid Diagnostic Tests for Imported Malaria in Clinical Practice: Results of a National Multicenter Study
Source: PLoS One. 2013 Sep 30;8(9):e75486. doi: 10.1371/journal.pone.0075486 (PMC3787089; doi:10.1371/journal.pone.0075486)
Supplement: Report of ethic committee S1 — Acceptance of the project by the Ethic Committee (in French). (PDF) [file pone.0075486.s003.pdf]

CPP Ile de France XI

Comité de Protection des Personnes

Saint-Germain-en-Laye, le 21 novembre 2006

Madame le Professeur Sophie MATHERON
Groupe hospitalier BICHAT –CLAUDE BERNARD
46, rue Huchard
75877 PARIS CEDEX 18

Titre de l'essai :	**Evaluation multicentrique des tests de diagnostic rapide pour le paludisme. AOR 06066**
Réf. CPP :	**06080**

Documents examinés	Numéro et date de version
Courrier de demande d'avis	30 octobre 2006

Madame,
Le CPP Ile de France XI a examiné dans sa séance du **16 NOVEMBRE 2006**, le projet de recherche référencé ci-dessus, pour lequel vous avez demandé un avis.

Cette étude ne relève pas de la loi 2004-806 du 9 août 2004 car elle ne modifie pas les conditions de prise en charge habituelle et ne présente pas de risque pour le patient. Il s'agit d'une étude relevant de l'art. l. 1221-8-1 du Code le la Santé Publique, menée à partir de prélèvements de sang prélevée dans une finalité médicale. Le sang ne doit pas être modifié dans ses caractéristiques et l'utilisation est possible si le prélèvement ne comporte que des risques négligeables. L'utilisation à des fins scientifiques ne requière pas un consentement spécifique.
Si un traitement des données médicales est envisagé, préciser que le fichier informatique utilisé a reçu l'autorisation de la CNIL, que le traitement est anonyme et que les sujets peuvent exercer leur droit d'accès et de rectification des données. Préciser que les patients ont la possibilité de contrôler les données qui y figurent ainsi que leur radiation au terme du délai légal.
Aucune rémunération ne doit être envisagée.
Nous avons relevé que l'information n'expliquait pas assez l'intérêt scientifique de cette étude. Préciser que les patients ne peuvent pas participer simultanément à une autre étude.

Ont participé à la délibération :

**I – PREMIER COLLEGE**		
**Didier ARMENGAUD**	***Pédiatrie***	*Titulaire*
**François BEAUFILS**	***Médecine interne***	*Titulaire*
**Pierre de TRUCHIS**	***Maladies infectieuses***	*Titulaire*
**Annie DURAND**	***Pharmacien hospitalier***	*Titulaire*
**Agnès GUIBERT- HOUDIARD**	***Biostat/Pharmaco***	*Titulaire*
**Annie HOUZET**	***Infirmière***	*Titulaire*
**Valérie LUCAS-JOUY**	***Médecine générale***	*Titulaire*
**Pascal PATRON**	***Médecine générale***	*Titulaire*
**Catherine BOURRET**	***Pharmacien Hospitalier***	*Suppléant*
**Sabine de la PORTE**	***Chercheur***	*Suppléant*
**Laurence MERIAN-BROSSE**	***Pharmacien hospitalier***	*Suppléant*
**II - DEUXIEME COLLEGE**		
**Jacques BERNARD**	***Alliance Maladies rares***	*Titulaire*
**Catherine DALLOZ**	**Magistrat**	*Titulaire*
**Thierry de la ROCHETTE DE ROCHEGONDE**	***Psychologue***	*Titulaire*
**Anne-Sophie GINON**	***Juriste – maître de conférences en droit***	*Titulaire*
**Christine STOUFFLET**	***Philosophe***	*Titulaire*
**Michèle CATZ**	***Psychologue***	*Suppléant*
**Max DANA**	***Ligue contre le cancer***	*Suppléant*
**Jean-François LAIGNEAU**	***Juriste***	*Suppléant*
**Olivier LANTRES**	***Avocat***	*Suppléant*

Aucun membre délibérant du comité n'est affecté par un conflit d'intérêt.

Le Président de la Séance,
Didier ARMENGAUD

20, rue Armagis
78105 Saint Germain en Laye Cedex
Tél : 01.39.27.42.58 Fax : 01.39.27.49.01
E.mail : cppidf11@chi-poissy-st-germain.fr
Bureau :
Didier ARMENGAUD, President
Thierry de ROCHEGONDE, Vice-président
Annie DURAND, Trésorière
Anne-Sophie GINON, Secretaire.
