# Supplementary material for: Performance of Rapid Diagnostic Tests for Imported Malaria in Clinical Practice: Results of a National Multicenter Study
Source: PLoS One. 2013 Sep 30;8(9):e75486. doi: 10.1371/journal.pone.0075486 (PMC3787089; doi:10.1371/journal.pone.0075486)
Supplement: Study protocol S1 — Detailed protocol of the research (in French). (PDF) [file pone.0075486.s004.pdf]

ASSISTANCE PUBLIQUE HÔPITAUX DE PARIS

Essai AOR06066

Evaluation multicentrique des tests de diagnostic rapide (TDR) pour le diagnostic du paludisme d'importation

PROTOCOLE TDR Palu

Gestionnaire : Assistance Publique – Hôpitaux de Paris (AP-HP)
Direction de la Politique Médicale
Département de la Recherche Clinique et du Développement (DRCD)
carré historique de l'hôpital Saint-Louis
1, avenue Claude Vellefaux – 75010 paris
www.drrc.ap-hop-paris.fr

Investigateur Coordonnateur	Responsable scientifique
Pr Sophie Matheron<br>Service des Maladies Infectieuses et Tropicales<br>Groupe hospitalier Bichat-Claude Bernard<br>46 rue Henri Huchard,<br>75018 Paris<br>Tél : 01 40 25 78 08<br>e-mail : sophie.matheron@bch.aphp.fr	Dr Sandrine Houzé<br>Laboratoire de Parasitologie,<br>Centre National de Référence sur le Paludisme (CNRP)<br>Groupe hospitalier Bichat-Claude Bernard<br>46 rue Henri Huchard, 75018 Paris<br>Tél : 01 40 25 78 81 Fax : 01 40 25 67 63<br>e-mail : sandrine.houze@bch.aphp.fr
**Chef de projet DRCD**<br>Christophe Aucan, PhD<br>DRCD - Hôpital Saint-Louis<br>1, Avenue Claude Vellefaux<br>75475 PARIS CEDEX 10<br>tel: 01 44 84 17 09<br>fax: 01 44 84 17 99<br>christophe.aucan@sls.aphp.fr	**Responsable URC Paris Nord**<br>Dr Isabelle Boutron, Dr Florence Tubach<br>Unité de Recherche Clinique Paris Nord,<br>Département d'Epidémiologie, Biostatistique et Recherche Clinique,<br>Groupe hospitalier Bichat-Claude Bernard<br>46 rue Henri Huchard, 75018 Paris<br>Tél : 01 40 25 73 87 Fax : 01 40 25 67 73<br>e-mail : isabelle.boutron@bch.aphp.fr

Equipes associées : Dr Nadine Godineau
Unité de Parasitologie-Mycologie
Hôpitaux de Saint-Denis
2 rue du Dr Delafontaine
93205 Saint-Denis
Tél : 01 42 35 60 75
e-mail : nadine.godineau@gmail.com

Dr Isabelle Poilane, Pr Anne Collignon
Laboratoire de Microbiologie
Hôpital Jean-Verdier
Avenue du 14 juillet
93000 Bondy
Tél : 01 48 02 65 73
e-mail : isabelle.poilane@jvr.aphp.fr

Dr Odile Fenneteau, Dr Nicole Schlegel
Service d'Hématologie Biologique,
Centre Hospitalier Universitaire Robert Debré
49 bd Sérurier, 75020 Paris
Tél : 01 40 03 23 64 Fax : 01 40 03 47 95
e-mail : odile.fenneteau@rdb.aphp.fr

Dr Véronique Lasserre, Dr Daniel Lusina
Laboratoire de Parasitologie
Hôpital Robert Ballanger
Boulevard Robert Ballanger
93 600 Aulnay-sous-bois
Tél : 01 49 36 71 19 Fax : 01 49 36 74 10
e-mail : veronique.lasserre@ch-aulnay.fr

Dr Anne-Sophie Leguern
Laboratoire du Centre Médical
Institut Pasteur
211 rue de Vaugirard
75015 Paris
Téléphone : 01 40 61 39 73 Fax : 01 40 61 39 49
e-mail : asleguer@pasteur.fr

Unité de Recherche Clinique :
Dr Isabelle Boutron, Dr Florence Tubach, Yolande Costa,
Delphine Prieur
Unité de Recherche Clinique Paris Nord,
Département d'Epidémiologie, Biostatistique et Recherche Clinique,
Groupe hospitalier Bichat-Claude Bernard
46 rue Henri Huchard, 75018 Paris
Tél : 01 40 25 73 87 Fax : 01 40 25 67 73
e-mail : isabelle.boutron@bch.aphp.fr

TABLE DES MATIERES

1. Résumé du protocole 5
TDR palu AOR 06 066 5

1. Introduction, justification de l'étude, résultats attendus et perspectives : 7

3. Données de la littérature et pré-requis 8

4. Objectifs de la recherche 11
4.1. Hypothèses testées 11
4.2. Objectif primaire 11
4.3. Objectifs secondaires 12

5. Plan expérimental 12
5.1. Choix du plan expérimental et justification 12
5.2. Critères de sélection des personnes 13
5.2.1. Critères d'inclusion 13
5.2.2. Critères d'exclusion 13
5.3. Calcul du nombre de sujets nécessaire 13
5.4. Mode de recrutement 13
5.5. Durée de participation de chaque personne ayant accepté de participer à la recherche 14

6. Schéma et conduite de la recherche. 14
6.1. Suivi des patients 14
6.2. Réalisation et interprétation des tests à l'étude 14
6.2.1 Mode de réalisation et conditions opératoires 14
6.2.2. Précautions d'emploi 15
6.2.3. Interprétation 15
6.3. Réalisation et interprétation du frottis et de la goutte épaisse 15
6.4. Transport des échantillons 16
6.5. Etude de la reproductibilité 16
6.6. Etude ancillaire 17
6.7. Durée totale de l'étude 17
6.8. Règles d'arrêt à remplir 17

7. Matériel évalué 17
7-1 Le NOW® ICT Malaria 18
7.2 l'OptiMAL-IT® 18
7-3 Palutop +4® 18
7-4 Core Malaria Pan/Pv/Pf 19
7-5 Résumé des tests de diagnostic évalués 20
7-6 Interprétation du résultat 20

8. Test de référence 21

9. Formation: 22

10 . Gestion des données et analyse statistique 22
10.1 Comité de pilotage 22
10.2 Description des méthodes statistiques incluant la planification d'éventuelles analyses intermédiaires 22
10.4 Responsable de l'analyse des données et logiciels de travail 22

10.5 Gestion des effets indésirables graves et monitorage des effets secondaires 23

11. Aspects légaux et éthiques 23

Notice d'information au patient et consentement 23
Déclaration CNIL 23
Transcription des données dans le cahier d'observation 24
Déclaration des événements indésirables graves aux Autorités de Santé........ 24
Extension de la recherche 24
Publications et propriétés des données........ 24

12 Bibliographie 24

ANNEXE 1 : fiche technique du test de diagnostic rapide Now® Malaria........ 27

ANNEXE 2 : fiche technique du test de diagnostic rapide OptiMAL-IT 29

ANNEXE 3 : fiche technique du test de diagnostic rapide PALUTOP+4®........ 32

ANNEXE 4 : fiche technique du test de diagnostic rapide Core Malaria Pan/Pv/Pf 37

1. Résumé du protocole

Evaluation multicentrique des TDR pour le diagnostic du paludisme d'importation
TDR palu AOR 06 066

Investigateur Coordonnateur Pr Sophie Matheron

Responsable scientifique Dr Sandrine Houzé

1. 1. Données de la littérature et prérequis scientifiques :

En France, le paludisme, est une maladie d'importation qui touche les voyageurs et les populations émigrées retournant dans leur pays avec une contamination en Afrique sub-saharienne dans 95% des cas. On estime que 5000 à 8000 cas de paludisme sont observés chaque année en France avec une vingtaine de décès annuel. *Plasmodium falciparum*, agent en cause dans 85% des accès est potentiellement létal. Les signes cliniques de l'accès palustre ne sont pas spécifiques et le diagnostic peut être facilement erroné vers une virose saisonnière. Le diagnostic repose sur l'examen microscopique d'un frottis sanguin et d'une goutte épaisse colorés lus par un microscopiste expérimenté. Alors que cette maladie peut se déclarer à tout moment, l'accès à un centre de diagnostic compétent conditionne une prise en charge efficace. Les tests de diagnostic rapide (TDR) du paludisme qui détectent des antigènes solubles palustres sont proposés pour le diagnostic du paludisme dans des structures sans accès à l'examen microscopique. Cependant, les résultats des études d'évaluation de la performance de ces tests sont contradictoires. Différents antigènes sont proposés pour le diagnostic tels que l'HRP2, histidin rich protein 2 ou la *Pf*LDH, *Plasmodium falciparum* lactate déhydrogénase, spécifiques de *Plasmodium falciparum*, la *p*LDH, *plasmodium* lactate déhydrogénase et l'aldolase, antigènes communs aux 4 espèces plasmodiales pathogènes pour l'homme.

1.2 Méthodologie :

Il s'agit d'une étude d'évaluation des performances diagnostiques des TDR sur les prélèvements des patients de tous âges, de retour de zone d'endémie palustre et se présentant avec une suspicion d'accès palustre aux services de consultations d'urgences hospitalières des sites participant à l'étude.

Cette étude sera réalisée conformément aux critères de qualité définis pour les études d'évolution diagnostique dans le cadre de l'initiative STARD (Standard for Reporting of Diagnostic Accuracy) et de l'outil QUADAS (Quality Assessment of Studies of Diagnostic Accuracy included in systematic reviews) [27, 28]. L'ensemble des malades inclus bénéficiera des 2 types de tests : les tests de diagnostic rapide pour le diagnostic du paludisme et l'examen de référence ou « gold standard », frottis et goutte

épaisse examinés aux laboratoires par un personnel expérimenté. Ces tests seront évalués indépendamment l'un de l'autre (en aveugle des résultats de l'autre test).

1. 3. Objectif :

L'objectif principal est la détermination de la sensibilité, de la spécificité, des valeurs prédictives positives, des valeurs prédictives négatives et du rapport de vraisemblance de la détection de chaque antigène.

1.4 . Matériel :

Quatre tests de diagnostic rapide détectant différents antigènes, Now® ICT Malaria (Inverness Médical), Palutop +4® (All Diag), Core Malaria Pan-Pv-Pf (Core Diagnostics), OptiMAL-IT (Diamed) seront testés en double insu et en parallèle dans 6 laboratoires de biologie (Bichat-Claude Bernard, Delafontaine, Institut Pasteur, Jean-Verdier, Robert-Ballanger, Robert-Debré) sur les échantillons sanguins des patients suspects de paludisme par rapport au frottis et à la goutte épaisse pris comme référence.

1. 5. Durée de la recherche et durée de participation du sujet :

L'étude sera réalisée dans 6 centres de la région parisienne, elle inclura 1225 patients environ pour étudier 245 patients atteints de paludisme. Cette recherche commencera en janvier 2007 et devrait durer 1 an. La participation de chaque sujet sera le temps de la consultation hospitalière.

1.6. Critères d'inclusion :

Les patients inclus seront les personnes consultant pour suspicion d'un accès palustre avec prescription d'un prélèvement pour effectuer un frottis et une goutte épaisse afin de diagnostiquer le paludisme. Aucun prélèvement supplémentaire ne sera effectué pour l'étude de ce protocole est donc une recherche non interventionnelle. Le patient recevra une information écrite sur la recherche.

1.7 : Résultats attendus :

Les performances annoncées par les fabricants sont comprises entre 93.4% et 100% pour les sensibilités et 96% à 100% pour les spécificités. Cependant, une étude préliminaire réalisée au laboratoire de parasitologie du CHU Bichat, a montré des sensibilités comprises entre 67% et 90% selon le test. Cette nouvelle étude multicentrique permettra d'établir les performances de ces tests dans la population concernée et éventuellement de proposer une combinaison optimale des antigènes détectés aux fabricants de dispositifs médicaux.

1. Introduction, justification de l'étude, résultats attendus et perspectives :

Alors que dans le monde on compte 300 à 500 millions d'accès palustres par an (OMS)[1], en France, le paludisme est une maladie d'importation que l'on estime à environ 5000 à 8000 cas annuels [2].
La symptomatologie clinique à type de syndrome grippal est peu spécifique et un diagnostic erroné d'affection virale est assez souvent porté entraînant un retard à la prise en charge spécifique. L'interrogatoire qui rapporte un séjour récent en zone d'endémie palustre est capital. Les accès palustres diagnostiqués en France ont été contractés en Afrique sub-saharienne pour 95% d'entre-eux et sont dus à *Plasmodium falciparum* dans 85% des cas avec un risque létal, les autres espèces (*Plasmodium ovale*, *Plasmodium vivax* et *Plasmodium malariae*) sont responsables des 15% restant, seule ou en association avec *Plasmodium falciparum* [2, 3]. Le diagnostic d'accès palustre est porté par la mise en évidence du parasite à partir d'un prélèvement sanguin par observation microscopique d'un frottis et/ou d'une goutte épaisse colorés. L'urgence vitale du diagnostic nécessite une mise à disposition 24h/24h de compétence technique et biologique pour le diagnostic. Cet examen ne fait pas partie des actes réservés, cependant le faible recrutement de certains centres ne permet pas aux biologistes de conserver un niveau d'excellence microscopique. Le frottis est réalisé partout alors que la goutte épaisse qui permet de détecter les faibles charges parasitaires n'est réalisée que dans certains laboratoires[4]. De nouveaux outils diagnostiques permettant la mise en évidence d'antigènes plasmodiaux par des tests immunochromatographiques sont apparus depuis 15 ans. Ces tests sont proposés aux biologistes comme complément au diagnostic microscopique. Cependant, les tests proposés sur le marché européen sont de qualités diverses en terme de sensibilité et de spécificité malgré leur marquage CE. Les performances de ces tests ont été essentiellement étudiées dans des zones d'endémie et n'ont pas été évalués les uns par rapport aux autres. Par ailleurs, il existe des discordances entre les résultats des différentes études publiées, financées en partie les industriels : Marx et collaborateurs[5] ont publié une revue de la littérature de ces évaluations et recommandent en conclusion de mener d'autres études contrôlées dans des centres non spécialisés. De plus, lors d'une étude préliminaire réalisée au laboratoire de parasitologie de Bichat-C Bernard[1], nous avons observé une sensibilité comprise entre 67% et 90% selon l'antigène détecté et le réactif utilisé, performances médiocres par rapport à celles rapportées par les industriels (90% à 100%)[6, 7]. De plus, les composants utilisés pour détecter les antigènes lors de la fabrication des tests de diagnostic rapide (TDR) sont fonction du fabricant et non connus et le choix du dispositif peut modifier les performances du test[7].
Le choix d'un TDR est une question récurrente. Les réactifs actuellement sur le marché ne répondent peut-être pas à la problématique du paludisme d'importation en France en terme d'espèces responsables ou de charge parasitaire.
Par conséquent, nous proposons de réaliser une étude multicentrique visant à

[1] Houzé S., Martineau F., Hubert V., Houzé P., Le Bras J. Diagnostic du paludisme : le danger des tests de diagnostic rapide. Société Française de Parasitologie, Paris, 15-16/12/05.

1) évaluer les performances de 4 tests diagnostics
2) évaluer les performances diagnostiques pour chaque antigène détecté par ces tests afin de déterminer quels seraient les réactifs permettant d'améliorer la performance de ces tests.

Les résultats de cette étude pourraient déboucher sur un partenariat industriel en vue de concevoir un réactif adapté à la situation française.

3. Données de la littérature et pré-requis

Un accès palustre se définit par l'association d'une fièvre et de la présence de plasmodiums circulants dans le sang du patient [8]. Le diagnostic repose sur la mise en évidence par la lecture d'un frottis coloré (ou d'une goutte épaisse) de formes asexuées de plasmodiums. Le seuil de détection de ces techniques microscopiques est d'environ 10 parasites/µl de sang. Ces techniques permettent le diagnostic de l'espèce plasmodiale responsable de l'accès qui conditionne la prise en charge thérapeutique mais elles exigent des compétences microscopiques et une remise à niveau régulière.

Différents antigènes solubles peuvent être recherchés dans le sang capillaire ou veineux d'un patient suspect de paludisme. Leur détection a été proposée depuis 15 ans comme aide au diagnostic biologique. Le premier antigène dont la détection a été développée est l'histidin rich protein 2 (HRP2), protéine soluble spécifique de *Plasmodium falciparum* qui est présente au niveau membranaire et cytoplasmique des globules rouges infectés [9]. Les premiers tests développés sur le principe d'une bandelette réactive (Parasight®, Becton Dickinson) présentaient des faux positifs, comme en présence de facteur rhumatoïde [10]. Ils ont été remplacés par des tests de deuxième génération tel que le Parachek® Pf présentés soit sous forme de bandelettes, soit sous forme de savonnettes ou cassettes de manipulation plus aisée [11]. Ces tests sont principalement destinés au diagnostic du paludisme en zone d'endémie quand les structures sanitaires de base n'ont pas accès à l'examen microscopique [12]. La *plasmodium* lactate déhydrogénase (*p*LDH) est une enzyme présente chez tous les plasmodiums [13]. Les anticorps monoclonaux produits pour sa détection reconnaissent soit une isoforme commune aux quatre espèces (*pan*LDH), soit une isoforme spécifique d'une espèce (*Pv*LDH pour le *P. vivax* par exemple) [14]. La *p*LDH est produite par les parasites viables, par conséquent sa recherche peut être utilisée pour suivre l'efficacité d'un traitement antimalarique [15]. Un autre antigène commun aux quatre espèces de plasmodiums, qui serait une aldolase, est également détecté par certains réactifs mais sa réactivité serait en défaut avec *Plasmodium ovale* [16].

De nombreux fabricants produisent des tests de diagnostic rapide (TDR) pour le paludisme : si les anticorps monoclonaux ont une même origine, en revanche la fabrication des tests (concentration, conjugué, qualité de la nitrocellulose) est propre à chacun. Sur le site de l'OMS (www.wpro.who.int/rdt), un certain nombre de dispositifs sont répertoriés de production et de composition diverses. Dans tous les cas, c'est le principe d'une immunochromatographie qui est mis en œuvre avec des anticorps monoclonaux spécifiques de l'antigène détecté immobilisés sur la bandelette. Un anticorps dirigé contre une protéine animale servira de témoin de réaction (schéma 1) .

Sur une même bandelette, on peut classiquement rechercher jusqu'à 3 antigènes différents. Le sang total est lysé par addition d'un tampon de lyse. Au niveau de la fenêtre réactionnelle, les anticorps monoclonaux conjugués à l'or colloïdal vont former un complexe antigène-anticorps avec les antigènes si ils sont présents dans l'échantillon testé. Les complexes migrent le long de la bandelette et seront stoppés par l'anticorps immobilisé. Une bande colorée traduira la fixation du complexe antigène-anticorps (schéma 1).

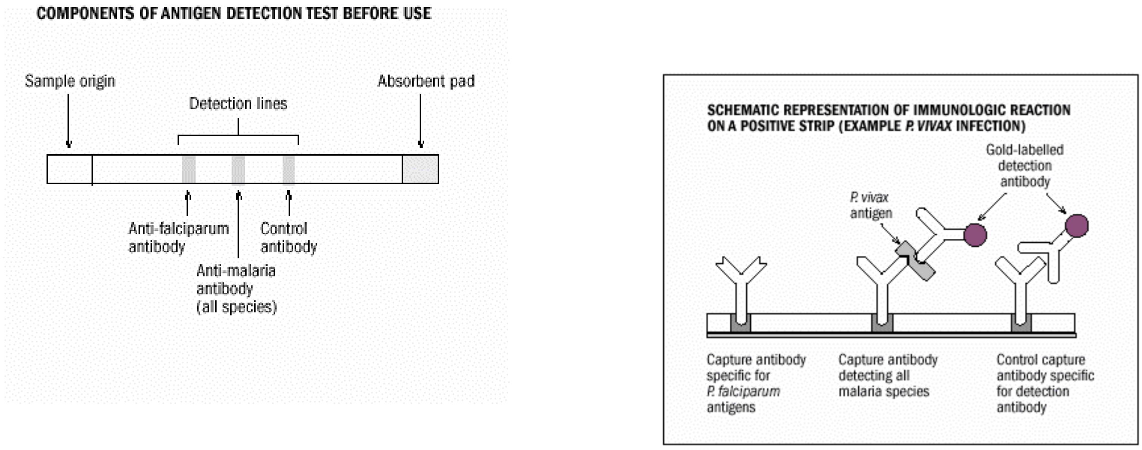

Schéma 1 : principe des tests de diagnostic rapide (www.who.int/rdt.)

La détection de ces antigènes a été étudiée dans le diagnostic du paludisme en zone d'endémie et les performances publiées sont fonction du réactif testé, de l'antigène recherché et de la situation épidémique locale. Des études réalisées en zone d'endémie palustre rapportent des sensibilités pour l'OptiMAL® (détection de la *p*LDH et de la *Pf*LDH) (Diamed) dans le diagnostic de l'accès palustre à *Plasmodium falciparum* entre 92% et 94.5% par rapport au frottis/goutte épaisse pris comme référence [17, 18]. Cependant, ces sensibilités sont fonction de la parasitémie et approchent des 20% en-dessous de 200 parasites/µl soit une parasitémie de 0.004% au frottis (environ la limite de détection d'un frottis positif).

La sensibilité de la recherche de l'HRP2 est proche de 100% pour le diagnostic de l'accès palustre à *Plasmodium falciparum* mais sa spécificité est moindre, comprise entre 67% et 98% selon les études [19, 20].

Les études réalisées dans pays hors zone d'endémie palustre, rapportent des sensibilités comprises entre 96 et 100% pour l'HRP2 et de 84% pour l'aldolase avec une spécificité de 99% environ [21, 22]. L'utilisation de l'OptiMAL dans des situations similaires a montré des sensibilités comprises entre 79 et 98% pour des spécificités évaluées entre 98 et 100% [23]. Ces études comparent un TDR par rapport à l'examen microscopique ou la recherche d'ADN plasmodial par biologie moléculaire mais ne comparent pas les tests entre-eux. De plus, les résultats des TDR sont considérés globalement positifs ou négatifs mais les performances ne sont pas évaluées selon l'antigène détecté. Enfin, tous les tests disponibles sur le marché n'ont pas fait l'objet d'études publiées alors que certaines études étaient

discutables sur le plan méthodologique et n'évaluaient pas la même population [5]. L'intérêt de la détection d'un antigène plutôt qu'un autre n'a pas été évalué ni le réactif utilisé pour détecter ces antigènes. La recherche simultanée de l'HRP2 et de la *p*LDH est préconisée par certains auteurs [24]. L'adjonction de la détection de la *Pf*LDH pourrait être complémentaire et permettre le diagnostic de souches non productrices d'HPR2 ou répondant partiellement avec les anticorps utilisés [25, 26].

Lors de notre étude réalisée dans le service, nous avons comparé 2 TDR recherchant pour l'un la *p*LDH et la *Pf*LDH (OptiMAL-IT®, Diamed) et pour l'autre la *p*LDH et l'HRP2 (Palutop +4®, Alldiag). Selon le réactif, la sensibilité de la détection de la *p*LDH parmi les 99 échantillons positifs à *Plasmodium falciparum* étaient de 71% avec le Palutop+4® et de 73% avec l'OptiMAL-IT®. La détection d'HRP2 (Palutop +4®) a montré une sensibilité de 90% (dont un vrai faux négatif avec un isolat non sécréteur d'antigène) et une spécificité de 96%. Nous n'avons pas pu évaluer d'autres tests en parallèle. Si les faux-positifs sont souvent expliqués par des accès palustres décapités par une auto-médication du patient, en revanche les faux négatifs peuvent avoir des conséquences dramatiques. Les évaluations financées par les fabricants sur échantillons sélectionnés ne rendent pas toujours compte des performances réelles des réactifs lors de leur utilisation de routine. Les industriels ne sont plus demandeurs d'évaluations dans ces conditions, car leur produit ont le marquage « CE » qui autorise leur mise sur le marché et des évaluations ont été produites pour ces dossiers de marquage. C'est pourquoi, des évaluations complémentaires réalisées en situation réelle de diagnostic sont nécessaires mais doivent être financées indépendamment des industriels pour garantir une liberté de réalisation [5].

En conclusion, la détection des antigènes plasmodiaux dans le diagnostic du paludisme a montré des spécificités et des sensibilités variables selon les études : la méthode optimale et en particulier le choix du réactif ne sont pas définis et nécessitent des études complémentaires réalisées sur la population spécifique des voyageurs de retour de zone d'endémie.

Faisabilité de l'étude

Cette étude est réalisable dans le cadre de l'appel d'offre pour les raisons suivantes :

1) L'étude ne nécessite aucun prélèvement autres que ceux réalisés systématiquement aux patients suspects d'accès palustre

2) La collecte des données est simple car toutes les données sont disponibles et évaluées dans les services de microbiologie et de parasitologie

3) Le nombre de patients requis est adapté aux capacités de recrutement de chacun des centres

4) Les équipes impliqués ont déjà collaboré sur des projets de recherche, sont correspondants du CNRP et sont complémentaires dans leur recrutement

Adéquation à l'appel d'offres

Ce projet correspond parfaitement à l'intitulé de l'appel d'offre : « Mise au point et évaluation d'un test diagnostique »

4. Objectifs de la recherche

4.1. Hypothèses testées

Les TDR ont des performances diagnostiques variables pour le diagnostic du paludisme d'importation en France en fonction de l'antigène recherché et de la population testée. Les performances comparée de ces tests n'ont jamais été évalués selon une méthodologie adaptée sur les patients consultant en urgence à qui sont destinés ces tests.

Notre hypothèse de travail repose sur le fait que

1) Il faut évaluer de manière indépendante et selon une méthodologie adaptée la performance de ces tests

2) En fonction des composants de ces tests, les performances diagnostiques pour chaque antigène détecté peuvent varier. Cette étude devrait permettre d'évaluer les performances diagnostiques de chaque réactif pour chaque antigène testé et ainsi de déterminer les réactifs à utiliser pour avoir une performance optimale.

4.2. Objectif primaire

Evaluer les performances diagnostiques chez les patients consultants pour suspicion d'accès palustre de 4 tests de détection rapide

- l'OptiMAL-IT produit par la société Diamed, détection de *p*LDH et *Pf*LDH,
- le Now® ICT Malaria distribué par la société Inverness France, détection de l'HRP2 et de l'aldolase,
- le Palutop+4® distribué par le société Alldiag, détection de la *p*LDH, de la *Pv*LDH et de l'HRP2,
- le Core Malaria Pan-Pv-Pf distribué par la société Ivagen, détection de la *p*LDH, de la *Pv*LDH et de l'HRP2.

Ces tests détectent 4 antigènes plasmodiaux, l'HRP2, la *Pf*LDH, la *p*LDH, et l'aldolase (et la *Pv*LDH pour 2 d'entre-eux) comme complément au diagnostic du paludisme chez des malades suspects cliniquement de paludisme. Ils ont été sélectionnés car ils sont distribués en France, ont répondu à l'appel d'offre des marchés de l'AP-HP et sont complémentaires.

4.3. Objectifs secondaires

1. Déterminer les performances diagnostiques respectives des différents TDR inclus dans l'étude en fonction des antigènes détectés et selon l'espèce plasmodiale diagnostiquée :
    - o Les antigènes *Pf* HRP2 (Histidin Rich Protein 2) et *Pf*LDH (*Plasmodium falciparum* lactico déhydrogenase), protéines spécifiques de *Plasmodium falciparum*,
    - o L'antigène *p*LDH (*Plasmodium* lactate déhydrogénase) et l'aldolase, antigènes plasmodiaux communs aux différentes espèces de plasmodiums

    seront recherchés parallèlement avec les TDR évalués sur tous les échantillons inclus.
2. Rechercher l'origine des faux-positifs et des faux-négatifs observés avec les TDR par des examens complémentaires : sérologie palustre, techniques de biologie moléculaire (PCR d'espèce palustre), dosages d'antipaludiques.

5. Plan expérimental

5.1. Choix du plan expérimental et justification

Il s'agit d'une étude d'évaluation des performances diagnostiques des TDR sur les prélèvements des patients de tous âges, de retour de zone d'endémie palustre et se présentant avec une suspicion d'accès palustre aux services de consultations d'urgences hospitalières des sites participant à l'étude.
Cette étude sera réalisée conformément aux critères de qualité définis pour les études d'évolution diagnostique dans le cadre de l'initiative STARD (Standard for Reporting of Diagnostic Accuracy) et de l'outil QUADAS (Quality Assessment of Studies of Diagnostic Accuracy included in systematic reviews) [27, 28]. L'ensemble des malades inclus bénéficiera des 2 types de tests : les tests de diagnostic rapide pour le diagnostic du paludisme et l'examen de référence ou « gold standard », frottis et goutte épaisse examinés aux laboratoires par un personnel expérimenté. Ces tests seront évalués indépendamment l'un de l'autre (en aveugle des résultats de l'autre test).
Pour obtenir une évaluation aveugle des 2 tests :
1) Les frottis seront réalisés dans le centre où consulte le patient, et les gouttes épaisses seront toute relues dans le service de parasitologie, CNRP de l'hôpital Bichat quelque soit le centre investigateur afin de standardiser les résultats.

2) L'évaluation des tests à l'étude sera réalisée dans les laboratoires des centres investigateurs. Les tests à l'étude seront interprétés par des biologistes en insu des résultats du frottis et de la goutte épaisse. Les frottis et les gouttes épaisses seront observés par des microbiologistes n'ayant pas connaissance des résultats des tests à l'étude.

5.2. Critères de sélection des personnes

5.2.1. Critères d'inclusion

Tous les patients se présentant dans les centres investigateurs :

- Pour un prélèvement pour réalisation d'un frottis goutte épaisse avec une suspicion clinique d'accès palustre
- Non opposition du patient après information

Ces critères d'éligibilités ainsi que le choix des centres recruteurs permettront d'étudier les performances de ces tests sur une population proche de celle qui devrait en bénéficier en pratique courante.

5.2.2. Critères d'exclusion

Il n'y a aucun critère d'exclusion

5.3. Calcul du nombre de sujets nécessaire

L'essai est construit afin de déterminer une estimation de la sensibilité des tests à l'étude avec une précision de 0.03 (demi-largeur de l'intervalle de confiance). La sensibilité attendue, au vu des résultats de laboratoire, est de 95%. Pour obtenir un intervalle de confiance à 95% avec une précision de 0.03, il faut inclure 245 patients positifs à *Plasmodium species*. Le standard sera la goutte épaisse effectuée par le laboratoire référent du CNRP.

Les prévalences des diagnostics positifs de paludisme parmi l'ensemble des demandes est d'environ 20% pour l'ensemble des centres participants à l'étude. L'inclusion de 245 patients adultes positifs à *Plasmodium sp.* nécessite d'inclure environ 1225 patients sur l'ensemble des sites participant à l'étude.

5.4. Mode de recrutement

Le nombre de patients nécessaires à l'étude devrait être atteint par les recrutements des 6 services associés : Bichat-Claude Bernard, Delafontaine, Institut Pasteur, Jean-Verdier, Robert-Ballanger, Robert-Debré. A l'hôpital Bichat-C Bernard, 197 diagnostics ont été posés en 2005, 88 à l'hôpital Delafontaine, 40 à l'Institut Pasteur, 50 à l'hôpital Jean-Verdier, 66 à l'hôpital Robert-Ballanger, 55 à l'hôpital Robert-Debré.

Les demandes restent globalement stables d'une année sur l'autre avec des pics saisonniers entre juillet et septembre correspondant aux retours des vacances d'été. Le protocole pourra être réalisé en une année.

5.5. Durée de participation de chaque personne ayant accepté de participer à la recherche

La durée de participation d'un patient à la recherche TDR Palu est le temps que dure la prise de sang.

6. Schéma et conduite de la recherche.

6.1. Suivi des patients

Les patients suspects d'accès palustres seront interrogés. Un recueil des renseignements épidémiologiques sera effectué : lieu de résidence, séjour en zone d'endémie (pays, durée, date de retour), prise d'une prophylaxie anti-palustre et observance, antécédent de thérapeutique anti-palustre. Ces renseignements sont collectés en pratique clinique courante par le clinicien et communiqués au biologiste responsable du frottis et de la goutte épaisse, ainsi qu'au biologiste responsable de la lecture des tests rapides. Les malades auront un prélèvement veineux au pli du coude pour frottis et goutte épaisse dans le cadre de leur prise en charge médicale.
Les tests de diagnostic rapide seront réalisés à partir du prélèvement veineux reçu dans les laboratoires pour le frottis et la goutte épaisse. L'étude ne nécessite donc pas de prélèvement supplémentaire par rapport à ceux nécessaires dans le cadre des soins. Il n'y a donc aucune modification dans la prise en charge des patients.

6.2. Réalisation et interprétation des tests à l'étude

6.2.1 Mode de réalisation et conditions opératoires

Les tests évalués dans cette étude ont été retenus parmi l'ensemble des réactifs disponibles sur le marché, par le marquage CE, la distribution en France et la nature des antigènes détectés. Au vu des informations actuelles, les tests retenus dans ce protocole seront :

- l'OptiMAL-IT distribué par la société Diamed France, détection de *p*LDH et *Pf*LDH,
- le Now® ICT Malaria distribué par la société Inverness France, détection de l'HRP2 et de l'aldolase,
- le Palutop+4® distribué par le société Alldiag, détection de la *p*LDH, de la *Pv*LDH et de l'HRP2,
- le Core Malaria Pan-Pv-Pf distribué par le société Ivagen, détection de la *p*LDH, de la *Pv*LDH et de l'HRP2.

Pour la réalisation des tests de diagnostic rapide, une fraction de sang total sera aliquotée à partir du tube de prélèvement veineux prélevé pour le frottis/goutte épaisse.

Les procédures des fabricants seront respectées. Les TDR seront réalisés par les différents sites participant à l'étude dans le laboratoire ayant reçu l'échantillon par un personnel qualifié différent de celui ayant fait le diagnostic d'accès palustre pour assurer l'évaluation en insu de ces tests. Les TDR peuvent être réalisés à distance du frottis/goutte épaisse (conservation 24 à 72h des échantillons à +4°C).

6.2.2. Précautions d'emploi

- Les composants de la trousse doivent être à température ambiante.
- Les TDR se présentent sous la forme d'un dispositif en emballage unitaire avec un dessicant. Le dessicant doit être conforme aux recommandations du fabricant à l'ouverture du sachet, sinon un autre dispositif doit être utilisé. Une fois le sachet ouvert, le test doit être utilisé immédiatement.
- Le sang ne doit pas avoir coagulé avant d'être transféré dans le test.
- Le résultat doit être lu dans les conditions recommandées par le fabricant.

6.2.3. Interprétation

Pour chaque bande potentielle, le résultat sera interprété en positif, négatif ou douteux et le TDR sera interprété selon les recommandations des fabricants par les investigateurs. Un résultat douteux sera considéré comme positif dans l'analyse statistique. Les TDR seront réalisés et interprétés en aveugle du résultat du frottis et de la goutte épaisse. Lorsqu'ils seront réalisés dans le cadre de ce protocole, leur résultat n'interviendra pas dans la prise en charge du patient.

Un cahier de recueil des données sera conservé dans les laboratoires sur lequel le numéro d'enregistrement de l'échantillon au laboratoire, l'identité du patient, la date de réalisation du test, la présence ou l'absence de chaque bande, le résultat interprété de chaque TDR et un commentaire sur les éventuelles difficultés de réalisation ou de lecture seront consignés.

Les résultats des TDR seront notés sur le test et dans le cahier d'observation. Pour les contrôles de qualité dans le cadre du monitoring, les TDR seront conservés toute la durée de l'étude pour vérification éventuelle de l'identité et de la saisie des résultats.

6.3. Réalisation et interprétation du frottis et de la goutte épaisse

Le test de référence sera le frottis associé à la goutte épaisse lue au CNRP réalisée sur les échantillons sanguins transmis par les centres investigateurs.

Les frottis seront réalisés selon les procédures en usage dans les laboratoires.

Les frottis sanguins des patients prélevés dans les hôpitaux de Bichat, Delafontaine, Jean-Verdier, Robert-Ballanger, Robert-Debré et au centre médical de l'Institut Pasteur seront techniqués et lus au laboratoire de biologie, de microbiologie, à l'unité de parasitologie, au laboratoire d'hématologie biologique ou au laboratoire de parasitologie de chaque centre.

Les échantillons prélevés et reçus pendant l'activité de garde seront techniqués par les équipes de garde selon les dispositions en vigueur dans chaque site et transmis aux laboratoires de jour. Pour tous, les lames faites en garde seront relues par les biologistes de jour. Les TDR seront réalisés sur les échantillons conservés à +4°C.

Il n'y aura pas de différence dans le traitement de l'échantillon, ni dans l'efficacité du diagnostic quelque soit l'heure ou le site d'analyse des prélèvements inclus.

A réception des échantillons, à partir de dix microlitres de sang total, il sera étalé un frottis mince de type hématologique et une goutte épaisse de 1cm de diamètre. Après séchage et fixation, le frottis sera coloré. Après séchage, la goutte épaisse sera déshémoglobinisée et colorée par le colorant de Giemsa à 7% en eau neutre. Les résultats des frottis seront rendus au clinicien par le biologiste après lecture de 40000 hématies et les gouttes épaisses après observation de 1000 globules blancs. Le seuil de détection du frottis est de 100 parasites/µl de sang et celui de la goutte épaisse de 10 parasites/µl de sang total dans ces conditions de lecture. L'observation de formes parasitaires asexuées constitue un résultat positif qui sera rendu en parasitémie (pourcentage de globules rouges parasités pour le frottis ou en nombre de parasites observés pour 1000 globules blancs sur la goutte épaisse) accompagnée du diagnostic d'espèce. La présence de gamétocytes de *Plasmodium falciparum* est notifiée mais ne constitue pas un résultat positif si ils ne sont pas associés à des formes asexuées.

Les résultats de la recherche de plasmodiums seront rendus dans les meilleurs délais aux cliniciens pour prise en charge du patient, par le laboratoire de parasitologie ou par le laboratoire multidisciplinaire de garde pour les patients de l'hôpital Bichat Cl Bernard et par les laboratoires respectifs pour les patients des autres hôpitaux. Les résultats des TDR réalisés dans le cadre de ce protocole ne seront pas considérés pour la décision diagnostique et ne seront pas transmis aux cliniciens.

6.4. Transport des échantillons

Le tube EDTA prélevé lors de l'inclusion du patient accompagné du CRF d'inclusion sera transporté au CNRP de Bichat pour réalisation du test de référence (frottis et goutte épaisse) et éventuellement de l'étude ancillaire. Ce transport aura lieu à +4°C dans les 24 à 48 h après le prélèvement. Pour les centres AP-HP, (Jean verdier, Robert Debré), il sera pris en charge par les coursiers AP-HP, pour les autres, par un transporteur spécialisé. Le déclenchement du transport se fera par le laboratoire de biologie du centre investigateur. Le CNRP de Bichat ne peut recevoir les prélèvements que les jours ouvrables (du lundi au vendredi et le samedi matin).

6.5. Etude de la reproductibilité

Afin d'évaluer la reproductibilité des différents tests :

- le test de référence : frottis, goutte épaisse
- les test à l'étude : l'OptiMAL-IT, le Now® ICT Malaria, le Palutop+4®, le Core Malaria Pan-Pv-Pf

30 prélèvements seront évalués de manière indépendante et en aveugle des résultats des autres tests par un biologiste non impliqué dans les autres phases de l'étude.

6.6. Etude ancillaire

En cas de discordances entre les TDR et le frottis et/ou la goutte épaisse, des examens complémentaires en vue de les expliquer seront réalisés au laboratoire de parasitologie de l'hôpital Bichat-C.Bernard : dosages d'antipaludiques (accès palustre décapité), sérologies palustres (diagnostic rétrospectif), PCR d'espèce (pour confirmation de l'espèce plasmodiale en cause), facteur rhumatoïde, HAMA (Human Anti Mouse Antibodies) sur les échantillons du laboratoire ou transmis par coursier par les différents centres investigateurs.

6.7. Durée totale de l'étude

Entre janvier 2007 et décembre 2007, soit 12 mois, pour l'inclusion des patients et la réalisation des TDR, l'analyse des données et la réalisation des tests complémentaires nécessaires.

6.8. Règles d'arrêt à remplir

Un recrutement insuffisant pourra motiver l'arrêt de l'étude : si à 50% de la durée prévisionnelle des inclusions malgré l'aide de l'URC auprès des investigateurs et les actions entreprises pour remédier à l'échec des inclusions, moins de 30% des patients ont été inclus, la DRCD est susceptible de décider de l'arrêt des inclusions, voire selon le contexte de l'arrêt prématuré de la recherche.

7. Matériel évalué

Quatre TDR seront évalués conjointement :

- l'OptiMAL-IT
- le NOW® ICT Malaria
- le Palutop +4®
- le Core Malaria Pan-Pv-Pf

7-1 Le NOW® ICT Malaria

NOW® ICT Malaria est un test rapide de détection des quatre espèces de *Plasmodium* (*P. falciparum*, *P. vivax*, *P. malariae*, *P. ovale*) dans le sang (cf notice descriptive en annexe 1). C'est un test réalisable sur sang total pour la détection qualitative de l'antigène *Pf* HRP-2 (histidin rich protein-2) spécifique de *P. falciparum* et d'un antigène commun aux 4 espèces de *Plasmodiums* (aldolase). Ce test ne nécessite aucune instrumentation et se présente sous la forme d'une carte-test.

NOW® ICT Malaria utilise le principe de l'immunochromatographie. Après dépôt du sang total et addition du tampon, l'échantillon migre le long de la membrane et les particules d'or colloïdal conjuguées avec les anticorps dirigés contre les antigènes plasmodiaux recherchés, vont se complexer avec l'antigène correspondant provenant de l'échantillon lysé. Le complexe migre le long de la membrane où il va être capturé par les anticorps monoclonaux spécifiques d'un antigène coatés sur la membrane sur 2 bandes distinctes conduisant ainsi à la formation d'une ou plusieurs bandes colorées. La fermeture de la carte permet au tampon déposé sur le revers d'imbiber la bande de nitrocellulose pour révéler la réaction colorée. L'absence de bande colorée au niveau d'un antigène indique un résultat négatif pour la recherche de celui-ci. Une bande contrôle qui doit être présente à l'issue du test permet de valider le résultat de celui-ci.

7.2 l'OptiMAL-IT®

L'OptiMAL-IT® est un test rapide de détection des quatre espèces de *Plasmodium* (*P. falciparum*, *P. vivax*, *P. malariae*, *P. ovale*) dans le sang (cf notice descriptive en annexe 2). C'est un test réalisable sur sang total pour la détection qualitative des antigènes *Pf*LDH spécifique de *P. falciparum* et *p*LDH commun aux 4 espèces de *Plasmodiums* (*pan* LDH). Ce test ne nécessite aucune instrumentation et se présente sous forme d'un dispositif avec bandelette et puits réactionnels.

L'OptiMAL-IT® utilise le principe de l'immunochromatographie. Après dépôt du sang total et addition d'une goutte de tampon dans un puit, l'échantillon migre le long de bandelette déposée dans le puit et les particules d'or colloïdal conjuguées avec les anticorps dirigés contre les antigènes plasmodiaux recherchés, vont se complexer avec l'antigène correspondant provenant de l'échantillon lysé. Le complexe migre le long de la membrane où il va être capturé par les anticorps monoclonaux spécifiques d'un antigène coatés sur la membrane sur 2 bandes distinctes conduisant ainsi à la formation d'une ou plusieurs bandes colorées. Après migration, la bandelette est transférée dans un 2ème puits contenant 4 gouttes de tampon. Après 10 minutes de lavage, la réaction peut être lue. L'absence de bande colorée au niveau d'un antigène indique un résultat négatif pour la recherche de celui-ci. Une bande contrôle qui doit être présente à l'issue du test permet de valider le résultat de celui-ci.

7-3 Palutop +4®

Palutop 4+® est un test rapide de détection des quatre espèces de *Plasmodium* (*P. falciparum*, *P. vivax*, *P. malariae*, *P. ovale*) dans le sang (cf notice descriptive en annexe 3). C'est un test réalisable sur sang total pour la détection qualitative des antigènes *Pf* HRP-2 (histidin rich protein-2) spécifique de *P. falciparum*, *Pv*LDH spécifique de *P. vivax* et *p*LDH commun aux 4 espèces de *Plasmodiums* (*pan* LDH). Ce test ne nécessite aucune instrumentation et se présente sous forme de cassette.
Palutop 4+® utilise le principe de l'immunochromatographie. Après dépôt du sang total et addition du tampon, l'échantillon migre le long de la membrane et les particules d'or colloïdal conjuguées avec les anticorps dirigés contre les antigènes plasmodiaux recherchés, vont se complexer avec l'antigène correspondant provenant de l'échantillon lysé. Le complexe migre le long de la membrane où il va être capturé par les anticorps monoclonaux spécifiques d'un antigène coatés sur la membrane sur 3 bandes distinctes conduisant ainsi à la formation d'une ou plusieurs bandes colorées. L'absence de bande colorée au niveau d'un antigène indique un résultat négatif pour la recherche de celui-ci. Une bande contrôle qui doit être présente à l'issue du test permet de valider le résultat de celui-ci.

7-4 Core Malaria Pan/Pv/Pf

Core Malaria Pan/Pv/Pf est un test rapide de détection des quatre espèces de *Plasmodium* (*P. falciparum*, *P. vivax*, *P .malariae*, *P. ovale*) dans le sang (cf notice descriptive en annexe 4). C'est un test réalisable sur sang total pour la détection qualitative des antigènes *Pf* HRP-2 (histidin rich protein-2) spécifique de *P. falciparum*, *Pv*LDH spécifique de *P. vivax* et *p*LDH commun aux 4 espèces de *Plasmodiums* (*pan* LDH). Ce test ne nécessite aucune instrumentation et se présente sous forme de cassette.
Le test utilise le principe de l'immunochromatographie. Après dépôt du sang total et addition du tampon, l'échantillon migre le long de la membrane et les particules d'or colloïdal conjuguées avec les anticorps dirigés contre les antigènes plasmodiaux recherchés, vont se complexer avec l'antigène correspondant provenant de l'échantillon lysé. Le complexe migre le long de la membrane où il va être capturé par les anticorps monoclonaux spécifiques d'un antigène coatés sur la membrane sur 3 bandes distinctes conduisant ainsi à la formation d'une ou plusieurs bandes colorées. L'absence de bande colorée au niveau d'un antigène indique un résultat négatif pour la recherche de celui-ci. Une bande contrôle qui doit être présente à l'issue du test permet de valider le résultat de celui-ci.

7-5 Résumé des tests de diagnostic évalués

Espèce	Antigènes recherchés	Tests de diagnostic rapide			
		OptiMAL-IT	Now® ICT Malaria	Palutop 4+®	Core Malaria Pan/Pv/Pf
P. falciparum	*Pf*HRP2		+	+	+
	*Pf*LDH	+			
4 espèces de *Plasmodium*	*p*LDH	+		+	+
	Aldolase		+		
P. vivax	PvLDH			+	+

7-6 Interprétation du résultat

Un résultat ne sera attribué au test de diagnostic rapide que si la bande C (contrôle) est apparente à l'issue du test. Un test de diagnostic rapide sera considéré comme positif si au moins une bande réactionnelle est présente (selon les recommandations du fabricant ci dessous). Un test de diagnostic rapide est considéré comme in interprétable si une bande réactionnelle au moins est présente mais la bande contrôle C est absente. Ces tests seront à refaire. En cas de persistance du résultat in interprétable, le résultat du test sera considéré comme manquant dans l'analyse.

Interprétation :

NEGATIF : seule la bande contrôle du test apparaît : bande C pour tous les tests

POSITIF pour *Plasmodium falciparum* ou en faveur d'une infection mixte *Plasmodium falciparum* et une autre espèce au moins de *Plasmodium* : les 2 bandes réactionnelles correspondant à la recherche de l'antigène de *Plasmodium falciparum* et à l'antigène commun aux 4 espèces sont présentes.
- bande T1 (antigène HRP2) et T2 (aldolase) pour le Now® ICT Malaria
- bande P (pLDH) et bande Pf (PfLDH) pour l'OptiMAL-IT
- bande P (pLDH) et bande Pf (HRP2) pour le Palutop+4® et le Core Malaria Pan/Pv/Pf

POSITIF à *P. falciparum* : présence de la bande réactionnelle pour l'antigène de *Plasmodium falciparum* mais absence de la bande commune aux 4 espèces. Cette situation peut être observée notamment sous traitement car les différents antigènes recherchés n'ont pas la même cinétique d'apparition ou de disparition, ou en présence d'un isolat dont les antigènes synthétisés ne sont pas reconnus par les TDR évalués.
- bande T1 (antigène HRP2) pour le Now® ICT Malaria
- bande Pf (PfLDH) pour l'OptiMAL-IT

- bande Pf (HRP2) pour le Palutop+4® et le Core Malaria Pan/Pv/Pf

POSITIF pour une autre espèce que *Plasmodium falciparum* : les bandes réactionnelles correspondant à la recherche de l'antigène commun aux 4 espèces sont présentes :

- bande T2 (aldolase) pour le Now® ICT Malaria
- bande P (*p*LDH) pour l'OptiMAL-IT
- bande P (*p*LDH) pour le Palutop+4® et le Core Malaria Pan/Pv/Pf. Pour ces 2 tests, en absence de la bande Pv, on peut exclure *Plasmodium vivax*.

Pour les tests Palutop+4® et Core Malaria Pan/Pv/Pf :

- **POSITIF pour *Plasmodium vivax* seul ou associé à une autre espèce *(P. ovale, P. malariae)*** : les bandes réactionnelles correspondant à la recherche de l'antigène spécifique à *Plasmodium vivax* et à l'antigène commun aux 4 espèces sont présentes : bande P (*p*LDH) et bande Pv (*Pv*LDH)

- **POSITIF pour *Plasmodium vivax* associé à *Plasmodium falciparum*** : les 3 bandes réactionnelles correspondant à la recherche de l'antigène spécifique à *Plasmodium vivax*, à l'antigène de *Plasmodium falciparum* et à l'antigène commun aux 4 espèces sont présentes : bande P (*p*LDH) et bande Pv (*Pv*LDH), bande Pf (HRP2).

8. Test de référence

Les test de référence est le résultat du frottis et de la goutte épaisse. Un cas de paludisme est défini par la présence sur le frottis et sur la goutte épaisse de stades asexués de *Plasmodium*. Le résultat doit préciser l'espèce(s) et la parasitémie. Les porteurs isolés de gamétocytes de *Plasmodium falciparum* ne sont pas considérés comme étant en accès palustre.

Le test de référence sera considéré comme positif si :

- le frottis et la goutte épaisse sont positifs (présence de formes asexuées de *Plasmodium*)
- le frottis est négatif et la goutte épaisse est positive (présence de formes asexuées de *Plasmodium*)

  Le frottis ne peut pas être positif et la goutte épaisse négative. La présence de gamétocytes isolés de *Plasmodium falciparum* n'est pas considérée comme un résultat positif.

Le test de référence sera considéré comme négatif si :

- le frottis et la goutte épaisse sont négatifs (absence de formes asexuées de *Plasmodium*)

9. Formation:

Les biologistes et les techniciens des centres investigateurs seront formés à la réalisation et à la lecture des tests de diagnostic rapide par Sandrine Houzé.

10 . Gestion des données et analyse statistique

10.1 Comité de pilotage

Un comité de pilotage sera constitué avec un représentant de chaque centre, des méthodologistes en charge du projet et des membres de la Délégation à la Recherche Clinique nommés pour cet essai. Ce comité est à l'origine du projet et réalisera l'écriture et la validation des cahiers d'observation. Il déterminera initialement la méthodologie et décidera en cours de recherche des conduites à tenir en cas d'imprévus, surveillera le déroulement de la recherche. Deux réunions par an seront prévues. Ces réunions permettront de vérifier la régularité des inclusions et le bon déroulement de l'étude. Ce comité sera également responsable de l'écriture des articles auxquels l'étude donnera lieu.

10.2 Description des méthodes statistiques incluant la planification d'éventuelles analyses intermédiaires

L'analyse principale évaluera la performance diagnostique des tests à l'étude pour le diagnostic des accès palustres en France. Seront donc estimés les sensibilités, spécificités, valeurs prédictives positives et valeurs prédictives négatives pour chaque antigène détecté et pour chaque réactif évalué. Les intervalles à 95% des estimations seront fournis.
En outre, l'usage qui en est fait lors du rendu du résultat aux cliniciens correspondra à du *rule out*, c'est-à-dire à rejeter rapidement un diagnostic d'accès palustre à *Plasmodium species*. Dans cette optique, le rapport de vraisemblance négatif sera calculé. Celui-ci permet d'évaluer la probabilité que le test soit d'avantage négatif chez un individu non malade que chez un individu malade.

10.3 Etude de la reproductibilité :

La reproductibilité des tests à l'étude et du test de référence sera évaluée par le calcul du pourcentage de concordance assorti de son intervalle de confiance à 95% entre les 2 évaluateurs et du coefficient kappa.

10.4 Responsable de l'analyse des données et logiciels de travail

Toutes les analyses seront effectuées au sein du département d'Epidémiologie, Biostatistique, Recherche clinique (Pr P. Ravaud) de l'hôpital Bichat Cl Bernard sous la responsabilité de Isabelle Boutron. Le logiciel utilisé sera SAS version 9.1.

Une déclaration de l'étude sera faite auprès de la Commission Informatique et Libertés (CNIL).

10.5 Gestion des effets indésirables graves et monitorage des effets secondaires

Cette étude ne modifie en rien la prise en charge des patients dans les services d'urgences participants. A priori, il n'y aura pas d'événement indésirable à déclarer.

11. Aspects légaux et éthiques

Notice d'information au patient et consentement

Cette étude n'entre pas dans le champs d'application de la Loi Huriet du 20/12/88 et ne suit pas les dispositions de la loi de Santé Publique du 9/08/2004 en application du 2ème alinéa de l'article L1121. En effet :

- l'étude ne modifie pas les conditions de prise en charge habituelle des patients.
- l'étude ne présente pas de risque supplémentaire par rapport aux conditions de soins et de suivis habituels des patients.

Afin de respecter la réglementation en vigueur et selon la loi du 06 Août 2004 du code de la santé publique, les sujets ne pourront participer à cette étude que s'ils ont reçu une information de la part de l'un des investigateurs sur :

- le but de cette étude,
- la durée de leur participation à cette étude,
- les procédures qui seront suivies,
- la confidentialité des données,
- le caractère volontaire de leur participation.

L'ensemble de ces informations est résumé sur une notice d'information remise à chaque patient et dont une copie est jointe en annexe 5.
L'étude sera soumis à un CPP pour accord sur la classification en recherche non interventionnelle.
L'étude sera enregistrée sur le site clinical trials.

Déclaration CNIL

La loi prévoit que la déclaration doit avoir été faite avant le début effectif de la recherche.
L'Unité de Recherche Clinique Paris Nord, responsable du fichier, assure la déclaration unitaire de la recherche auprès du Comité consultatif sur le traitement de l'information en matière de recherche dans le domaine de la santé puis de la CNIL.

Transcription des données dans le cahier d'observation

Toutes les informations requises par le protocole doivent être fournies dans le cahier d'observation et une explication donnée par l'investigateur pour chaque donnée manquante.
Les données devront être transférées dans les cahiers d'observation au fur et à mesure qu'elles sont obtenues qu'il s'agisse de données cliniques ou para-cliniques. Les données devront être copiées de façon nette et lisible à l'encre noire dans ces cahiers (ceci afin de faciliter la duplication et la saisie informatique). Les données erronées dépistées sur les cahiers d'observation seront clairement barrées et les nouvelles données seront copiées sur le cahier avec les initiales et la date par le membre de l'équipe de l'investigateur qui aura fait la correction. L'anonymat des sujets sera assuré par la mention au maximum des initiales du sujet sur tous les documents nécessaires à la recherche, ou par effacement par les moyens appropriés (blanc correcteur…) des données nominatives sur les copies des documents source, destinés à la documentation de la recherche.
Les données informatisées sur un fichier seront déclarées à la CNIL selon la procédure adaptée au cas.

Assurance qualité-contrôle de qualité

Aucun monitoring sur site ne sera effectué pour cette recherche

Déclaration des événements indésirables graves aux Autorités de Santé

Toutes les suspicions d'effet indésirable grave inattendu seront déclarées à l'autorité compétente dans les délais légaux.

Extension de la recherche

Toute extension de la recherche (modification profonde du schéma thérapeutique ou des populations incluses, prolongation des traitements et ou des actes thérapeutiques non prévus initialement dans le protocole) devra être considérée comme une nouvelle recherche.

Publications et propriétés des données

Seront premiers signataires des publications, les personnes ayant réellement participé à l'élaboration du protocole et son déroulement ainsi qu'à la rédaction des résultats.
L'AP-HP doit être mentionné comme soutient financier de cette étude sur la publication. La notion AP-HP doit être mentionnée dans l'adresse des auteurs.

12 Bibliographie

1. WHO. Malaria, 1982-1997. *Wkly Epidemiol Rec.* 1999, 74 : 265-72.

2. Legros F, Danis M, Noireau E. Caractéristiques épidémiologiques du paludisme d'importation en France métropolitaine (CNRMI, données 1999-2000). *Bull CNRMI*. 2002 ; 16-17.
3. Ralaimazava P. *et al*. Chimiosensibilité du paludisme importé en France en 2001 et 2002. *BEH* 2004 ; 6 :21-24.
4. Prise en charge et prévention du paludisme d'importation à *Plasmodium falciparum*. 12ème Conférence de consensus thérapeutique de la SPILF. HIA Bégin, Saint-Mandé, 14 avril 1999.
5. Marx A., Pewsner D., Egger M. *et al*. Meta-analysis of rapid tests for malaria in travelers returning fromendemic areas. *Ann. Intern. Med*., 2005, 142; 836-846.
6. De Monbrison F., Gérome P ., Chaulet J.F. *et al.* Comparaison diagnostic performance of two commercial rapid tests for malaria in a non-endemic area. *Eur J Clin Microbiol Infect Dis*. 2004 ; 23 : 784-786.
7. Moody A, Chiodini P. Non-microscopic method for malaria diagnosis using OptiMal IT, a second-generation dipstick for malaria *p*LDH antigen detection*. Br J Biomed Sci*. 2002; 59: 228-31.
8. Gentilini M. Paludisme. In Médecine Tropicale, Médecine-Sciences, Ed. Flammarion, 1993.
9. Howard R.J, Shigehiko U., Aikawa M. *et al.* Secretion of a Malarial Histidine-rich Protein (Pf. HRP II) from *Plasmodium falciparum*-infected Erythrocytes. *J. Cell Biol*. 1986; 103: 1269-1277.
10. Iqbal J., Khalid N., Hira P. Performance of rapide malaria Pf antigen test for the diagnosis of malaria and false-reactivity with autoantibodies. *Adv Exp Med Biol.* 2003 ; 531 : 135-48.
11. Singh N, Saxena A. Usefulness of a rapid on-site *Plasmodium falciparum* diagnosis (Paracheck ®Pf) in forest migrants and among the indigenous population at the site of their occupational activities in central India. *Am J Trop Med Hyg*. 2005; 72 : 26-9.
12. Huong NM, Davis TM, Hewitt S. *et al*. Comparaison of three antigen detection methods for diagnosis and therapeutic monitoring of malaria: a field study from southern vietnem. *Trop Med Int Health*. 2002; 7: 304-8.
13. Piper R., Le Bras J., Wentworth L. *et al*., Immuno-capture diagnostic assays for malaria utilizing *Plasmodium* Lactate Dehydrogenase (pLDH) *Am J Trop Med Hyg*. 1999; 60: 109-118
14. Fernando S., Karunamera N., Fernando W. Evaluation of a rapid whole blood immunochromatographic assay for the diagnosis *of Plasmodium falciparum* and *Plasmodium vivax* malaria. *Ceylon Med J*. 2004 ; 49 : 7-11.
15. Moody A., Hunt-Cooke A., Gabbett E., Chiodini P. . Performance of the OptiMAL® malaria antigen capture dipstick for malaria diagnosis and treatment monitoring. *Br J of Hematol*. 2000; 109: 1-5.
16. Farcas G., Zhong K., Lovegrove F., *et al*. Evaluation of the Binax Now ICT test versus polymerase chain reaction and microscopy for the detection of malaria in returned travelers. *Am J Trop Med Hyg*. 2003 ; 69 : 589-92.

17. Congpuong K. Comparaison of the Optimal rapid test with routine microscopic examination of Giemsa-stained thick blood film for diagnosis of malaria. *J Med assoc Thai*. 2001; 84 : 357-63.
18. Khan S., Anwar M., Hussain S. *et al*. Comparaison of Optimal malarial test with light microscopy for the diagnosis of malaria. *J Pak Med Assoc*. 2004; 54 : 404-7.
19. Singh N, Shukla M. Short report : field evaluation of posttreatment sensitivity for monitoring parasite clearance of *Plasmodium falciparum* malaria by use of the determine malaria pf test in central india. *Am J Trop Med Hyg.* 2002; 66: 314-6.
20. Iqbal J., Siddique A., Jameel M. *et al.*. Persistent histidine-rich protein 2, parasite lacate dehydrogenase, and panmalarial antigen reactivity after clearance of *Plasmodium falciparum* monoinfection. *J Clin Microbiol.* 2004 ; 42 : 4237-41
21. Richter J., Harms G., Muller-Stover I. *et al*. Performance of an immunochromatographic test for the rapid diagnosis of malaria. *Parasitol Res.* 2004 ; 92 : 518-519.
22. Bouchaud O, Houzé S., Longuet C. *et al*. Use of the Parasight-F diagnostic test for imported malaria in travel clinic. *Am J Trop Med Hyg*. 2000; 63: 76-79.
23. Palmer C., Bonilla J., Bruckner D. *et al*. Multicenter study to evaluate the Optimal test for rapide diagnosis of malaria in US hospitals. *J Clin Microbiol.* 2003; 41: 5178-82.
24. Grobusch M., Hanscheid T., Gobels K. *et al.* Comparasion of three antigen detection tests for diagnosis and follow-up of falciparum malaria in travellers returning to Berlin, Germany. *Parasitol Res.* 2003 ; 89 : 354-357.
25. Baker J., McCarthy J., Gatton M. *et al*. Genetic diversity of *Plasmodium falciparum* histidine-rich protein2 (PfHRP2) and its effect on the performance of PfHRP2-based rapid diagnosis tests. *JID*. 2005; 192: 870-877
26. Kemp D., Thompson J., Walliker D. *et al*. Molecular karyotype of *Plasmodium falciparum*: conserved linkage groups and expendable histidine-rich protein genes. *Proc. Natl. Acad. Sci*., 1987; 84: 7672-7676.
27. Bossuyt P., Reitsma J., Bruns D. *et al*. Towards complete and accurate reporting of studies of diagnostic accuracy: the STARD initiative. *Clin Chem*. 2003; 49: 1-6.
28. Whiting P., Rutjes A., Reitsma J. *et al*. The development of QUADAS: a tool for the quality assessment of studies of diagnostic accuracy included in systematic reviews. *BMC Med Res Method*. 2003; 3: 1-13.

CE

ANNEXE 1 : fiche technique du test de diagnostic rapide Now® Malaria

binax

Vital Answers For Better Health...NOW™

Manufactured by:
Binax, Inc.
217 Read Street
Portland, Maine 04103 USA
Telephone (207) 772-3988
Fax (207) 761-2074
Website http://www.binax.com
Email: info@binax.com

NOW® Malaria Test for Whole Blood

Catalog No. 660-000

Rev. 6 9/30/04
IN660000

CE

EC REP

EMERGO EUROPE
P.O. BOX 18510
2502 EM THE HAGUE
THE NETHERLANDS

NOW® *MALARIA*

A RAPID WHOLE BLOOD IMMUNOCHROMATOGRAPHIC TEST (ICT)
(U.S. Patent No(s): 5,877,028; 5,998,220; 6,017,767)
for the qualitative detection of *Plasmodium falciparum* (P.f.), *Plasmodium vivax* (P.v.), *Plasmodium malariae* (P.m.) and *Plasmodium ovale* (P.o.) antigens
For *in vitro* diagnostic use.

Test Principle

The NOW® Malaria test is a rapid, *in vitro* immunodiagnostic test for the detection of circulating *Plasmodium falciparum* (P.f.) antigen and an antigen that is common to all four species of malaria, *Plasmodium falciparum* (P.f.), *Plasmodium vivax* (P.v.), *Plasmodium ovale* (P.o.) and *Plasmodium malariae* (P.m.) in whole blood. The test uses two antibodies that have been immobilized across the test strip. One antibody is specific for the histidine-rich protein II antigen of *P. falciparum* (Pf. HRPII). The other antibody is specific for an antigen that is common to P.f., P.v., P.m. and P.o.

A procedural control line is also immobilized across the test strip and will always appear in area C of the test window, if the test has been performed correctly. Whole blood (15μL) is applied to a sample pad impregnated with colloidal gold-labeled antibodies, which are directed against the malarial antigens. When a positive sample is applied, malarial antigens bind to the gold-conjugated antibodies in the sample pad. Reagent is then added which allows the immune complexes formed to migrate along the test strip where they are captured by the immobilized antibodies. When capture occurs, one or two pink lines will form in the test window. When a negative sample is applied, only the control line will appear.

Acknowledgement

Binax, Inc. acknowledges F. Hoffman La Roche Corporation for the provision of antibodies used in this test.

Specimen Collection

Use an EDTA capillary tube, capable of delivering 15μl, or venous blood collected into EDTA tubes.

A) To obtain capillary blood via puncture of a finger, heel or other appropriate site, cleanse the area with a sterile swab and dry with a sterile pad. Use a lancet to puncture the skin and collect the blood directly into the capillary tube. Fill the entire capillary tube with blood and use immediately.

B) Collect venous blood, by the standard venipuncture procedure, into an EDTA tube. If the test cannot be performed immediately, the blood may be stored for up to three days at 2° to 8°C.

Precautions and Warnings

- Optimal results will be obtained by strict adherence to this protocol. Reagents must be added carefully to maintain precision and accuracy. Treat used cards as biohazardous. Do not reopen or reuse test cards.
- Biological contamination of dispensing equipment, containers or reagents can lead to false results. Observe established precautions against microbiological and serological hazards in specimen handling, disposal and throughout all procedures.
- Do not use kits beyond their expiration date. Keep storage boxes dry.
- Store kits at 15° - 30° C.
- Reagent A contains sodium azide as a preservative. Sodium azide is toxic and should, therefore, be handled carefully, avoiding ingestion or skin contact. It may react with lead or copper plumbing to form explosive metal azides. Flush with a liberal volume of water when disposing of unwanted reagent.
- Do not mix reagents from different kit lots.

Limitations of Procedure

The test is able to identify an infection caused by P.f. or one that is caused by either P.v., P.o. or P.m. The test cannot speciate a P.f. mixed infection as the antibody used on test line 2 may be positive in patients with P.f., P.v., P.o., and P.m. Performance characteristics of this test have been evaluated using P.f. and P.v. samples only. Occasionally, residual P.f. HRPII antigen may be detected for several days following elimination of the parasite by anti-malarial treatment. Diagnosis should be made using the results of this test together with the other clinical and laboratory findings.

Kit Contents

- Individually packaged test cards.
- Capillary tubes.
- Malaria Reagent A.
- Product Insert.
- Procedure card.

Materials Required but not Included

- Lancets.
- Sterile swabs.

NOW® ICT *MALARIA*

En snabb immunokromatografisk test i helblod
(U.S. Patent No(s): 5,877,028; 5,998,220; 6,017,767)
för kvalitativ detektion av *Plasmodium falciparum* (P.f.), *Plasmodium vivax* (P.v.), *Plasmodium malariae* (P.m.) och *Plasmodium ovale* (P.o.) antigener.
För diagnostisk användning *in-vitro*.

Testprincip:

NOW® ICT MALARIA är en snabb *in-vitro* immunodiagnostisk test för detektion av cirkulerande *Plasmodium falciparum* (P.f.) antigen och ett antigen som finns i alla fyra specier av malaria, *Plasmodium falciparum* (P.f.), *Plasmodium vivax* (P.v.), *Plasmodium ovale* (P.o.) och *Plasmodium malariae* (P.m.) i helblod. Testen använder sig av två antikroppar som har fixerats på var sitt band över testmembranen. Den ena antikroppen är specifik för den histidinrik protein II antigenet av P. falciparum (Pf. HRPII). Den andra antikroppen är specifik för en antigen som finns på P.f., P.v., P.m. och P.o.

En procedurkontrollinje har också fixerats på ett band över testmembranen. Detta band kommer alltid att visas i området C av testfönstret om testen har genomförts korrekt. Helblod (15μL) tillsätts ett provmembran som är impregnerade med de malariaantikropparna som är redan konjugerade med "colloidal gold". Om provet innehåller malariaantigener kommer dessa antigenr att binda sig till de konjugerade antikropparna i provmembranet. En reagens tillsätts som möjliggör dessa immunkomplexer att migrera längs testmembranet där de fångas av de fixerade antikropparna. I detta fall skulle antingen ett eller två rosa band visas i testfönstret utöver kontrollbandet. Om provet är negativt kommer endast kontrollbandet att visas.

Tillkännagivande:

Binax, Inc. Tillkännager att F. Hoffman La Roche Corporation levererar antikropparna som används i denna test.

Provtagning:

Använd EDTA kapillärrör som kan leverera 15μl, eller venblot som har tagits i ett EDTA-rör.

A) Vid kapillärrörsprovtagning vid t.ex. finger- eller hälprick skall hudområdet rensas med en steril bommelsepinne och torkas med en steril membran. Använd en lansett att punktera huden och ta blodet direkt i kapillärröret. Fyll hela kapillärröret med blot och använd direkt.

B) Ta venblod enligt standard venblodtagningsprocedur i ett EDTA rör. Om testen inte kan köras direkt kan blodet förvaras vid 2-8 °C i tre dagar.

Försiktighetsråd och varningar:

- Optimalresultat kan fås om man följer strikt proceduren. Reagenserna måste tilsättas försiktigt för att upprätthålla precision och ackuratess. Behandla redan använda tester som biologisk riskavfall.
- Biologisk kontaminering av dispenseringsapparater, behållare eller Reagenserna kan förorsaka felaktiga resultat. Använd etablerade mikrobiologiskt och serologiskt försiktighetsråd under procedurerna.
- Använd inte en testförpackning efter utgångsdatumet. Förvara lådarna torrt.
- Förvara testförpackningen mellan 15° - 30° C.
- Reagens A innehåller natriumazid som ett konserveringsmedium. Natriumazid är toxist och bör därmed behandlas försiktigt, undvik intagning och kontakt med hud. Natriumazid kan binda med koppar eller bly i rörledningssystemet och producera explosiva azider. Spola med mycket vatten när man häller ut oanvända reagenser.
- Blanda inte olika lotnummer.

Procedurens limitationer:

Testen kan endast identifiera om en infektion orsakas av P.f. eller om en infektion orsakas av antingen P.v., P.o. eller P.m. Testen kan inte specificera en blandinfektion eftersom antikroppen som finns på linje 2 kan vara positivt i patienter med P.f., P.v., P.o. och P.m. Testens resultat har endast utvärderas med prover som innehåller P.f. och P.v. Ibland kan P.f. HRPII antigen identifieras flera dagar efter en anti-malariabehandling. Diagnos bör göras med resultat av denna test tillsamman med andra kliniska och laboratorieresultat.

Testförpackningsinnehåll:

- Enförpackade reagenskort
- Kapillärrör
- Malaria reagens A.
- Bruksanvisning
- Procedurkort.

Material som behövs men inte finns med:

- Lanseter.
- Sterila membraner

NOW® *MALARIA*

Test rapido immunocromatografico su sangue intero (ICT)
per la ricerca qualitativa diretta degli antigeni di:
Plasmodium falciparum (P.f.), *Plasmodium vivax* (P.v.), *Plasmodium malariae* (P.m.) e *Plasmodium ovale* (P.o.)
(U:S: Patent No(s): 5,998,220; 6,017,767)
Per uso diagnostico *in vitro*.

Principio di funzionamento del test

Il test NOW® Malaria è un test rapido immunocromatografico, per la ricerca dell'antigene specifico del *Plasmodium falciparum* (P.f.) ed un antigene che è comune a tutte e quattro le specie conosciute di malaria, *Plasmodium falciparum* (P.f.), *Plasmodium vivax* (P.v.), *Plasmodium ovale* (P.o.) e *Plasmodium malarie* (P.m.) nel sangue intero.

Il test usa due anticorpi che sono fissati in due diverse bande nella zona di lettura della striscia. Un anticorpo è specifico per l'antigene HRPII (Proteina ricca d'istidina). Tipico per il P. falciparum. L'altro anticorpo è specifico per un antigene che è comune a P.f., P.v., P.m. and P.o. Una linea di controllo inoltre è fissata all'estremità della zona di lettura (C). Questa banda di controllo deve sempre comparire nella zona C per garantire che il test è stato eseguito correttamente. Il sangue (15μl.) viene applicato nella striscia con un diluente, che reidrata un primo anticorpo marcato con Oro colloidale diretto contro gli antigeni malaria. In presenza di antigeni malaria si forma un immunocomplesso che migra verso l'altra estremità della membrana. L'immunocomplesso viene catturato dai rispettivi anticorpi specifici fissati nella zona di lettura della membrana. In caso di positività, si formano una o due linee rosa-viola nella finestra del test. In caso di campione negativo, deve comparire solo la linea di controllo (C).

Riconoscimento

Binax, Inc ringrazia la società di F. Hoffman La Roche per la fornitura di gli anticorpi usati in questo test.

Raccolta del campione

Usare un capillare trattato con EDTA, adatto a prelevare e ad erogare 15μl. di campione, oppure usare sangue venoso raccolto in provette con EDTA.

A) Per ottenere sangue capillare, pungere con una lancetta sterile il polpastrello, il tallone o altro luogo adatto, pulire ed asciugare la zona con un tampone sterile. Se si usa la lancetta o un pungidito per perforare la pelle, raccogliere il sangue direttamente nel tubo capillare. Riempire l'intero tubo capillare di sangue ed usarlo immediatamente.

B) Raccogliere il sangue venoso, in una provetta con EDTA. Se il test non può essere eseguito immediatamente, il sangue può essere conservato in frigo per tre giorni a 2° - 8° C.

Precauzioni ed Avvertenze

- Per ottenere le massime prestazioni analitiche dal prodotto, seguire scrupolosamente le istruzioni per l'uso. I reagenti devono essere aggiunti con attenzione per esser certi della massima precisione ed accuratezza dei risultati. Le cards usate sono da trattare come rifiuto biologico. Non riaprire e non riutilizzare le cards usate.
- Evitare la contaminazione biologica dei prodotti o dei reagenti. Osservare le normali precauzioni contro il rischio microbiologico e sierologico quando si esegue l'analisi e per l'eliminazione dei campioni usati.
- Non usare il kit dopo la data di scadenza, Conservare i kit in luogo asciutto a 15-30° C.
- Il reagente A contiene Sodio Azide, come conservante.
- La Sodio Azide è tossica, si deve maneggiare con attenzione , evitare l'ingestione ed il contatto con la pelle. Può reagire con il rame ed il piombo formando una metallo azide esplosiva. Lavare con acqua abbondante i lavandini dove si getta il prodotto.
- Non mescolare reagenti di lotti differenti

Limitazioni della Procedura

Il test è in grado di identificare un infezione causata da P.f. oppure una causata da P.v., P.o. o P.m. Il test non può specificare un'infezione mista di P.f. come un anticorpo usato sulla linea del test 2 potrebbe essere positivo in pazienti con P.f., P.v., P.o e P.m. Le prestazioni caratteristiche di questo test sono state ottenute utilizzando solo campioni infetti con P.f. e P.v. Occasionalmente, un residuo circolante di antigene di P.f. HRPII può essere rilevato dopo diversi giorni dall'eliminazione del parassita in trattamento con farmaci antimalarici. La diagnosi deve essere fatta tenendo conto dei risultati di questa prova unitamente agli altri risultati di laboratorio e altri segni clinici.

Componenti del kit

- Test Cards confezionate singolarmente
- Tubi Capillari
- Malaria Reagente A
- Istruzioni per l'uso
- Metodica breve

Materiale necessario ma non fornito nel kit

- Lancette pungidito
- Tamponi sterili

NOW® *MALARIA*

TEST IMMUNOCHROMATOGRAPHIQUE RAPIDE (ICT)
sur sang total
pour la détection qualitative de l'antigène HRP-II du *Plasmodium falciparum* (P.f.) et d'un antigène commun aux espèces *P. falciparum, P. malariae* (P.m.), *P. ovale* (P.o.) et *P. vivax* (P.v.)
(Brevet US No(s): 5,877,028; 5,998,220; 6,017,767)
USAGE IN VITRO

PRINCIPE DU TEST

Le test NOW® Malaria est un test de diagnostic immunologique in vitro, sur sang total, qui permet la détection de l'HRPII (protéine spécifique du *P. falciparum*) et d'un antigène commun aux 4 espèces de Plasmodium (*P.falciparum, P.malariae, P.ovale et P.vivax*). Le test utilise 2 anticorps de capture fixés sur la membrane : un des anticorps est dirigé contre l'antigène HRPII (spécifique du P.f.), bande T1. L'autre anticorps détecte un antigène commun aux 4 espèces de Plasmodium (P.f., P.m., P.o. et P.v.), bande T2. Le test comporte également une bande-contrôle permettant de valider le résultat. Ce contrôle doit toujours apparaître dans la fenêtre de lecture (bande C). Le sang total est déposé sur la zone de dépôt-échantillon (papier-buvard violet) imprégnée d'anticorps marqués à l'or colloïdal dirigés contre les antigènes palustres. - Dans le cas d'un échantillon positif, les antigènes palustres se lient aux anticorps marqués (zone de dépôt). Le réactif ajouté permet aux complexes antigène-anticorps formés de migrer le long de la membrane où ils seront capturés par les anticorps de capture. Deux ou trois bandes apparaissent alors dans la fenêtre de lecture. - Dans le cas d'un échantillon négatif, seule la bande de contrôle apparaît dans la fenêtre de lecture.
N.B. : Origine des anticorps : F. Hoffman La Roche Corporation.

PRELEVEMENT

Utiliser du sang capillaire ou veineux (15μl) prélevé dans un tube EDTA.

Désinfecter la zone de prélèvement et sécher avec une compresse stérile.
A) Prélèvement du sang capillaire par ponction au doigt ou au talon :
Piquer la peau à l'aide d'une lancette et recueillir le sang directement dans le tube capillaire. S'assurer que le tube capillaire est rempli au maximum et l'utiliser immédiatement.
B) Prélèvement du sang par ponction veineuse dans un tube EDTA.
Si l'analyse ne peut pas s'effectuer immédiatement, l'échantillon peut être conservé 3 jours à +2°...8°C.

PRECAUTIONS D'EMPLOI

- Suivre strictement le mode opératoire. Les cartes-test utilisées doivent être considérées comme du matériel potentiellement infectieux. Ne pas réutiliser les cartes-test.
- Respecter les précautions d'emploi pour éviter les contaminations microbiologiques et sérologiques lors de la manipulation et de la destruction des prélèvements.
- Ne pas utiliser les coffrets au-delà de la date de péremption. Maintenir les coffrets au sec.
- Conserver les coffrets à 15°...30° C.
- Le réactif A contient de l'azide de sodium (conservateur). L'azide de sodium est toxique et doit être manipulé avec précaution pour éviter son ingestion ou son contact avec la peau.
Il peut réagir avec le plomb et le cuivre des canalisations pour former des composés explosifs. Il est recommandé de rincer les canalisations après usage.
- Ne pas utiliser des réactifs provenant de lots différents.

LIMITES DU TEST

Le test permet l'identification d'une infection à P.f. ou d'une infection à P.m., P.o. ou P.v. Ce test ne permet pas de préciser s'il s'agit d'une infection mixte car, du fait de la nature de l'anticorps utilisé, la ligne T2 peut être positive avec P.f., P.m., P.o. et P.v. A ce jour, les performances de ce test ont été évaluées en utilisant uniquement des échantillons P.f et P.v. positifs. Un résidu d'antigène HRPII peut éventuellement être détecté plusieurs jours après l'élimination par un traitement anti-paludéen du parasite. Dans tous les cas, il est nécessaire d'intégrer l'ensemble des données cliniques épidémiologiques et biologiques avant d'établir le diagnostic final.

CONTENU DU COFFRET

- Cartes-test conditionnées séparément.
- Tubes capillaires.
- Réactif A.
- Notice d'utilisation
- Schéma de prélèvement

MATERIEL NECESSAIRE MAIS NON FOURNI

- Lancettes.
- Compresses stériles.

Preliminary Clinical Data

Note: Binax does not make any claims based on the studies which have been reported in the insert. Further studies are currently underway to accurately determine performance.

1. Walter Reed Army Institute of Research, Maryland USA; Preliminary Evaluation of the NOW® ICT Malaria P.f./P.v. rapid diagnostic device for the detection of *Plasmodium falciparum* and *Plasmodium vivax*.

Published Article: Am J Trop Med. Hyg. 2001; 65(3 Suppl.):320-1 (Abstract)

Results: Reference Method: Microscopy

	Total Malaria	Malaria P.f.	Malaria P.v.
Sensitivity	93.4% (100/107)	100% (31/31)	89% (58/65)
Specificity		96% (206/215)	98% (177/181)

Note: Blood samples used in this study have not been fully characterized as they relate to parasitemia.

2. Hôpital d'Instruction des Armées Bégin, Paris France

Note: Study not currently published.

Results: Reference Method: PCR and Microscopy

	Total Malaria	Malaria P.f.	Malaria P.v.
Sensitivity	91.7% (22/24)	95% (21/22)	100% (1/1)
Specificity	100% (30/30)		

Note: I. P.O. sample has not been reported in sensitivity claims.

Reference Method: *P. falciparum* sensitivity versus Microscopy only

	Malaria P.f.
Sensitivity	100% (20/20)
Specificity	97%* (31/32)

*One sample appears as a false positive-but only against microscopy. Sample confirmed positive by PCR.

Note: Blood samples used in this study have not been fully characterized as they relate to parasitemia.

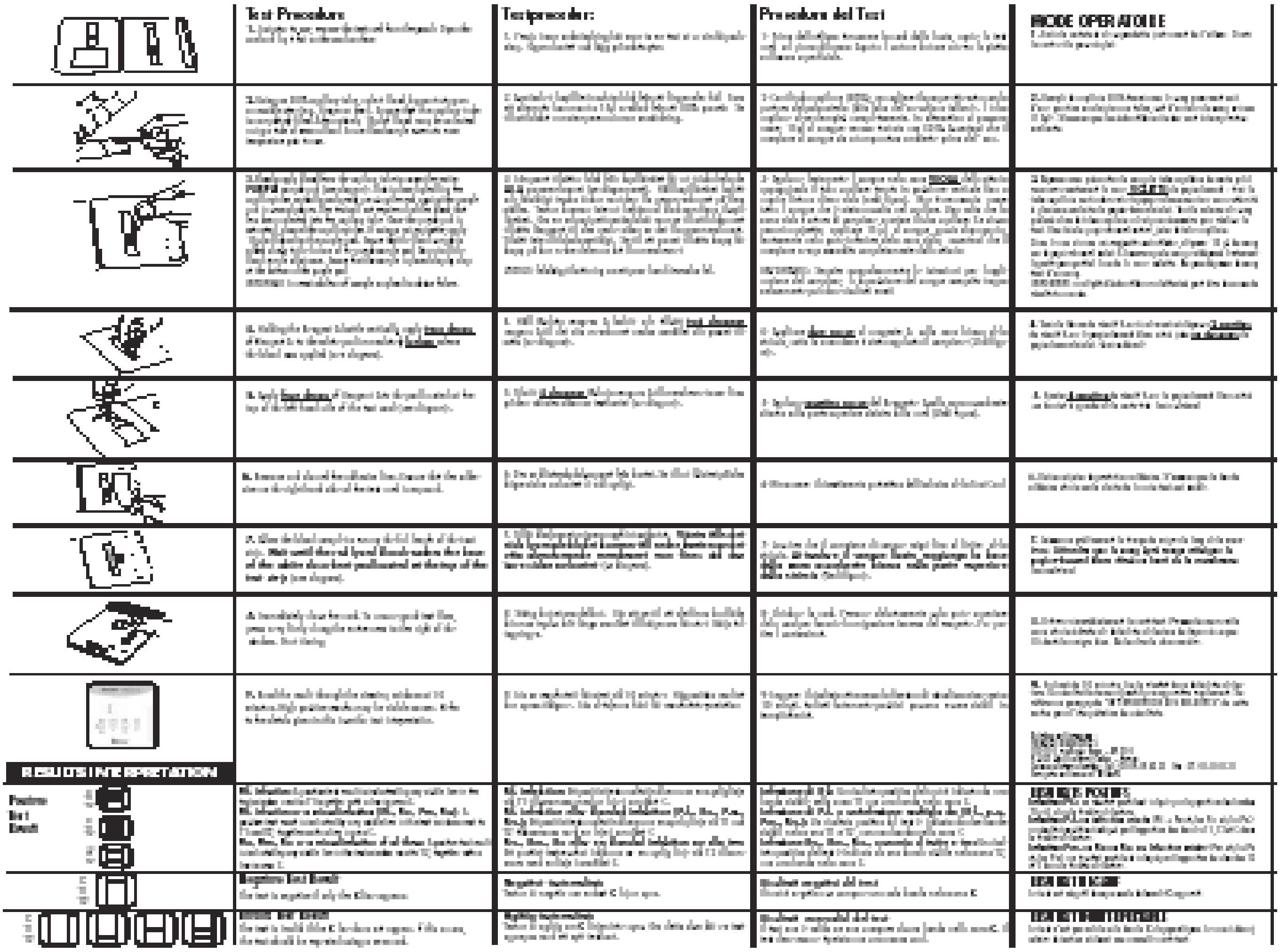
Test Procedure
Procedura del Test
MODE OPERATOIRE

ANNEXE 2 : fiche technique du test de diagnostic rapide OptiMAL-IT

B710000 11.01 DiaMed

Français

OptiMAL Rapid Malaria Test

DiaMed OptiMAL Rapid Malaria Test se prête à la détection d'une infection par Plasmodium species (Malaria ou Paludisme), indiquant:

1) La présence ou l'absence de Plasmodium sp.
2) Le diagnostic différentiel de Plasmodium sp.:
   - P. falciparum (agent responsable des cas mortels de Malaria)
   - P.vivax, P. malariae ou P. ovale

Chaque année, le nombre de cas de Malaria observés dans le monde est estimé à près de 500 millions, dont près de 3 millions ont une issue fatale, en particulier chez l'enfant et la femme enceinte.

Caractéristiques bio-médicales de DiaMed OptiMAL:

Specificité: le test détecte la présence de **lactate déhydrogénase de Plasmodium (pLDH)**, une enzyme produite par les formes sexuées et asexuées du parasite.
La présence de pLDH est révélée par l'utilisation d'anticorps monoclonaux dirigés contre les isoformes de l'enzyme. Il n'y a pas de réaction croisée avec la LDH humaine.

Sensibilité: le test permet de détecter une parasitémie de 100-200 parasites par µL de sang (résultat correspondant à une parasitémie de 0.002% à 0.004%). La sensibilité du DiaMed OptiMAL Rapid Malaria Test peut être comparée à une observation microscopique d'au moins 30 minutes d'un frottis sanguin mince avec un objectif x 100 à immersion.

Simple: ce test nécessite une seule goutte de sang (10 µL) capillaire prélevé directement au bout du doigt, ou de sang total anticoagulé.

Rapide: la réponse est obtenue en 20 minutes.

Contrôle du traitement d'un accès palustre:

le test n'est positif que lorsque les parasites sont présents dans le sang circulant. Il se négative habituellement dans les 4-5 jours suivant un traitement efficace.
Par conséquent, ce test peut être employé pour mettre en évidence des souches de Plasmodium sp. résistantes à certains traitements et peut être utilisé dans le cadre d'un suivi post-thérapeutique.
Il est souhaitable d'effectuer ce contrôle toutes les 48 heures: le signal positif avant le traitement diminuera lors du 2ème contrôle, puis sera négatif lors du 3ème contrôle en cas de succès thérapeutique.

Réactifs

IVD OptiMAL Rapid Malaria Test	Coffret de 48 tests, contenant:	Coffret de 24 tests, contenant:
DiaMed OptiMAL Bandelettes	48 pièces	24 pièces
DiaMed OptiMAL Puits-conjugué	48 pièces	24 pièces
DiaMed OptiMAL Puits-lavage	48 pièces	24 pièces
DiaMed OptiMAL Support pour puits	1 pièce	1 pièce
DiaMed OptiMAL Tampon	2 flacons de 3.5 mL	1 flacons de 3.5 mL
DiaMed OptiMAL Pipettes	48 pièces	48 pièces
Lancettes stériles	48 pièces	48 pièces
Tampons désinfectants	48 pièces	24 pièces

2 °C – 30 °C *Stabilité: voir date de péremption sur les étiquettes.*

Important: la présence d'humidité peut diminuer la stabilité des réactifs. Il est donc très important de prendre le nombre de Bandelettes et Puits-conjugué nécessaires pour le test et de refermer ensuite immédiatement les emballages.

Attention: lorsque les réactifs DiaMed OptiMAL sont utilisés comme indiqué, ils ne présentent aucun risque pour l'utilisateur. Eviter tout contact avec la peau, les yeux et les muqueuses, font partie des procédures de bonne pratique des analyses de laboratoire. Ne pas pipeter à la bouche un réactif ou du sang à tester.

Contrôle positif (disponible séparément)

DiaMed OptiMAL Positive control, correspondant à un signal obtenu avec un échantillon sanguin ayant une parasitémie faible (P.falciparum) inférieure à 1.0%.
Il est recommandé d'utiliser le contrôle positif à chaque nouveau coffret et après ouverture des emballages, si les Bandelettes et les Puits-conjugué n'ont pas été utilisés pendant plus d'une semaine.
Le contrôle s'utilise exactement comme un échantillon de sang de patient.

Echantillons

- Du sang capillaire prélevé au bout du doigt, voir Procédure ci-dessous.
- Du sang total prélevé selon les méthodes habituelles de prise de sang veineux, utiliser des tubes contenant un anticoagulant (EDTA, héparine).

Méthode

Si conservés à 2-8 °C, ramener les composants à température ambiante avant utilisation.
Toujours fermer les emballages après avoir pris le nombre nécessaire de Bandelettes et de Puits avec conjugué.

1. Sortir une Bandelette du récipient, écrire l'identification du patient sur l'étiquette (NB: toujours tenir la Bandelette par l'étiquette, ne pas toucher la zone de réaction).
2. Placer 1 Puits-conjugué et 1 Puits-lavage dans le Support.
3. Distribuer 1 goutte (environ 20 µL) de Tampon au Puits-conjugué et 4 gouttes (environ 80 µL) au Puits-lavage. Attendre 1 minute.
4. Nettoyer la surface de la peau du doigt avec le Tampon désinfectant, laisser sécher puis piquer la partie latérale du doigt avec la Lancette stérile (jeter le Tampon désinfectant et la Lancette dans une poubelle appropriée).
5. Prendre une Pipette, presser doucement le tube et immerger l'extrémité ouverte dans la goutte de sang. Ensuite relâcher doucement la pression pour aspirer la goutte de sang dans la Pipette. Lors de prise de sang veineuse, préveler le sang avec la Pipette à partir du tube de prélèvement.
6. Ajouter 1 goutte (environ 10 µL) de sang au Puits-conjugué. Mélanger doucement avec l'extrémité fermée de la même Pipette. Attendre 1 minute (jeter la Pipette dans une Poubelle appropriée).
7. Placer la Bandelette verticalement dans le Puits-conjugué et la laisser dans le puits pendant 10 minutes (le sang montera vers le filtre et la ligne de contrôle apparaîtra progressivement).
8. Transférer la Bandelette du Puits-conjugué au Puits-lavage et la laisser jusqu'à ce que le sang ne soit plus présent sur la zone de réaction et la ligne de contrôle soit clairement visible (durée: 5 à 10 minutes).
9. Sortir la Bandelette du Puits-lavage et lire les réactions (voir "Interprétation du test").
10. Conserver la Bandelette testée comme référence ultérieure et pour le contrôle d'un suivi thérapeutique.

B710000 11.01

Version 1

Interprétation des résults

A) Principe

Le Puits-conjugué DiaMed OptiMAL contient un indicateur lié à un anticorps monoclonal (hybridome de souris) contre pLDH, séché à la surface. Cet anticorps monoclonal réagit avec toutes les iso-enzymes LDH du genre Plasmodium.

Il y a 3 anticorps séchés sur la Bandelette DiaMed OptiMAL, dans l'ordre suivant:

- anti-souris (de chèvre) comme contrôle de la procédure technique
- anti-pLDH (monoclonal) spécifique aux 4 espèces de Plasmodium sp. (*falciparum, vivax, malariae, ovale*)
- anti-pLDH (monoclonal) spécifique à *Plasmodium falciparum*

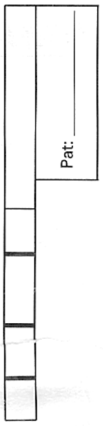

B) Validation du test

Le test peut être considéré comme valable quand le sang n'est plus présent sur la zone de réaction de la Bandelette et que la bande de contrôle est clairement visible, présentant une ligne dense. Dans ce cas, le résultat peut être interprêté.

Le test est non valable dans 2 circonstances:
1) Lorsque la bande de contrôle n'apparaît pas.
2) Lorsque la Bandelette est insuffisamment lavée (la zone de réaction reste rouge); il se peut que le volume de tampon était insuffisant ou le temps de lavage trop court.
Dans les deux cas, ne pas interpréter les résultats. Répéter le test, en suivant exactement la méthode décrite.

C) Interprétation des résultats

Réaction positive: la pLDH présente dans l'échantillon réagit avec l'anti-pLDH conjugué et migre sur la Bandelette où elle sera capturée par l'un ou les deux anticorps spécifiques contre pLDH, provoquant l'apparition d'une bande colorée.

Réaction négative: la pLDH n'est pas détectée dans l'échantillon. Aucune réaction n'a eu lieu avec les anticorps contre pLDH et seule la bande de contrôle sera visible.

D) Modèle des réactions

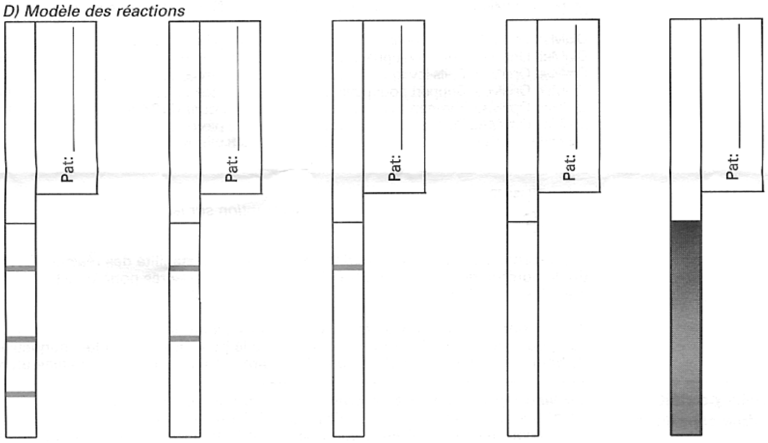

Positif pour P. falciparum | **Positif** pour P. vivax/P. malariae P. ovale | **Négatif** | **Test non valable** | **Test non valable**

C) Suivi post-thérapeutique

Répéter le test après 2 jours de traitement (48 heures)* puis à nouveau 2-3 jours plus tard* et comparer les réactions obtenues à celles du test avant le traitement:

- peu ou pas de différence des réactions: --> indique un traitement inefficace
- diminution de la réaction positive après 2 jours et résultat négatif au 4-5ème jour: --> indique un traitement efficace

* Remarque: cette durée peut être plus longue selon le traitement utilisé. Dans ce cas, répéter le contrôle 7 et 10 jours après le début du traitement. Si la réaction reste positive avec la même intensité du 5ème au 10ème jour, la possibilité d'une souche de Plasmodium sp. résistante au traitement doit être évoquée.

Limites

- Toutes modifications des procédures décrites ci-dessus ou l'utilisation d'autres réactifs peuvent influencer les résultats et rendre le test non valable.
- Ne pas mélanger et utiliser des réactifs, Bandelettes et Puits-conjugués de différents lots.
- Ne pas oublier que le résultat du test DiaMed-OptiMAL doit être interprété en tenant compte du contexte épidémiologique, clinique et thérapeutique. Quand cela semble être indiqué, le recours aux techniques parasitologiques de référence (examen au microscope d'une goutte épaisse et d'un frottis sanguin mince) devrait être considéré.

Bibliographie

1. Makler M.T., Pamer C.J. and Ager A.L.: A review of practical techniques for the diagnosis of malaria. Ann. Trop. Med & Parasitol. 1998, 92 (4), 419-433
2. Palmer C.J., Lindo J.F., Klaskala W.I., Quesada J.A., Kaminsky R., Baum M.K., Ager A.L.: Evaluation of the OptiMAL test for rapid diagnosis of Plasmodium vivax and Plasmodium falciparum malaria. J Clin. Microbiol, 1998 Jan. 36(1): 203-6.
3. Quintana M., R. Piper, H.-L. Boling, M. Makler, C. Sherman, E. Gill, E. Fernandez, S. Martin: Malaria diagnosis by dipstick assay in a honduran population with codendemic Plasmodium falciparum and Plasmodium vivax. Am.J.Trop.Med.Hyg., 1998, 59(6):868-871.
4. Moody A., Hunt-Cooke A., Gabette E. and Chiodini P.: Performance of the OptiMAL malaria antigen capture dipstick for malaria diagnosis and treatment monitoring at the Hospital for Tropical Diseases, London. British Journal of Haematol. 2000, 109, 891-894.

Produits

OptiMAL Rapid Malaria Test	Coffret de 48 tests	n°. cat. 710000
OptiMAL Rapid Malaria Test	Coffret de 24 tests	n°. cat. 710001
OptiMAL Contrôle positif	1 x 8 puits	n°. cat. 710011

Mandataire CE: DiaMed France S.A., 9 rue Française, F-75002 Paris

DiaMed SA, 1785 Cressier s/Morat, Suisse
sous licence de FLOW Inc., Portland, OR 97201, USA.

CE

CE

ANNEXE 3 : fiche technique du test de diagnostic rapide PALUTOP+4®

Test rapide de détection des quatre espèces de Plasmodium
(*P. falciparum*, *P. vivax*, *P. malariae*, *P. ovale*) dans le sang

Réf. N° : 5481
Pour usage *in vitro* uniquement

OBJECTIF

PALUTOP+4 *est un test rapide réalisable sur sang total pour la détection qualitative des antigènes Pf HRP-2 (histidine rich protein-2) spécifique de* P.falciparum*, pLDH spécifique de* P.vivax *et pLDH commun à toutes les espèces de Plasmodium (pan espèces). Le test peut être utilisé pour la détection spécifique de* P falciparum *et* P.vivax*, ou pour le diagnostic différentiel des autres espèces (*P.malariae, P ovale*) et pour le suivi des traitements antimalariques. Le test* ***PALUTOP+4*** *est destiné à une utilisation sur sang total et ne nécessite aucune instrumentation*

RESUME

Quatre espèces de *Plasmodium* sont responsables d'infections chez l'homme: *P falciparum, P.vivax, P.ovale* et *P.malariae*. Parmi ces quatre espèces *P. falciparum* et *P.vivax* ont la plus forte prévalence. Un dépistage précoce et un diagnostic différentiel d'espèce sont essentiels car l'espèce *falciparum* est responsable des formes cérébrales et est fréquemment résistante aux antimalariques. Le traitement étant dépendant de l'espèce, un diagnostic différentiel entre *P. falciparum* et *P.vivax* est primordial pour une meilleure prise en charge et une guérison rapide.
Le système de détection de **PALUTOP+4** pour *P. falciparum* repose sur la détection de l'antigène Pf HRP-2 (histidine rich protein –2) qui est une protéine soluble dans l'eau qui est larguée par les hématies parasites de sujets infectés. Le système de détection de *P.vivax* repose sur la mise en évidence d'une pLDH spécifique de *P.vivax*. La détection des autres espèces (*P.ovale* et *P.malariae)* repose sur la détection d'une pan pLDH.
La pLDH est produite par les parasites viables; par conséquent, la bande pan pLDH peut aussi être utilisée pour suivre l'efficacité d'un traitement antimalarique.
PALUTOP+4 détecte la présence de la Pf.HRP-2 de *P. falciparum*, de la pLDH spécifique de *P.vivax* et de la pan pLDH commune aux quatre espèces sur sang total. **PALUTOP+4** est un test spécifique et sensible de détection des 4 espèces de *Plasmodium*, de diagnostic différentiel de *P. falciparum* et *P.vivax* et de suivi du traitement antimalarique.

PRINCIPE

PALUTOP+4 utilise le principe de l'immunchromatographie. Après addition du tampon, l'échantillon migre le long de la membrane et les particules d'or colloïdal conjuguées avec les anticorps anti HRP-2 , anti pLDH spécifique de *P. vivax* et anti pan pLDH commun aux quatre espèces vont se complexer avec l'antigène correspondant provenant de l'échantillon lysé. Le complexe migre le long de la membrane où il va être capturé au niveau des bandes correspondantes où sont coatés les anticorps monoclonaux anti Pf. HRP-2, anti pLDH spécifique de *P. vivax* et anti pan pLDH conduisant ainsi à la formation d'une ou plusieurs bandes colorées mauves. L'absence de bande colorée au niveau d'une région test indique un résultat négatif pour l'antigène correspondant. Les particules d'or colloïdal non complexées et l'anti sérum de lapin marqué à l'or colloïdal vont migrer le long de la membrane jusqu'au niveau de la bande contrôle où est immobilisé un anticorps anti lapin entraînant ainsi l'apparition d'une coloration mauve. Cette bande contrôle permet de valider le bon fonctionnement du test.

LIMITES DU TEST

Le diagnostic clinique définitif ne peut être porté par le praticien que sur la base des autres données cliniques et biologiques. **PALUTOP+4** ne peut se substituer à l'examen microscopique et ne doit être utilisé qu'en complément.
En cas d'infection à *P.vivax* ou *P.falciparum*, ou d'infection mixte à *P.vivax* et *P.falciparum* la bande pan est également positive empêchant ainsi la différenciation avec *P.ovale* ou *P.malariae*.
En cas de suivi de traitement et si la réactivité du test reste identique 5 à 10 jours après le traitement, il faut envisager la présence de souches résistantes au traitement.
Habituellement, les bandes Pv et pan se négativent après un traitement antimalarique approprié. Néanmoins, la nature et la durée du traitement agissent au niveau de l'élimination du parasite, le test doit être répété 5 à 10 jours après le début du traitement.
Chez *P falciparum*, l'antigène HRP-2 n'est pas sécrété au stade gamétogonie. Par conséquent, la bande HRP-2 peut être absente chez les porteurs.
Le taux de HRP-2 persiste jusqu'à 15 jours après le traitement. La bande pan peut être utilisée pour vérifier l'efficacité du traitement en cas d'infection à *P falciparum*.
Dans certains cas, la présence de la bande HRP-2 et l'absence de la bande pan peuvent indiquer une phase post traitement. Néanmoins, un tel profil peut aussi être rencontré dans de rares cas de paludisme non traité. Face à un tel profil, il est conseillé de retester 2 jours plus tard.
Des absences de bandes Pan ont été notées dans certaines infections à *Plasmodium ovale.*

MATERIEL FOURNI

10 sachets aluminium contenant :
Une Savonnette : Membrane contenant des anticorps monoclonaux anti-HRP-2 conjugués à l'or colloïdal, des anticorps monoclonaux anti pLDH spécifique de *P.vivax* conjugués à l'or colloïdal et des anticorps monoclonaux anti pan pLDH conjugués à l'or colloïdal, des globulines de lapin conjuguées à l'or colloïdal ainsi que des monoclonaux anti Pf. HRP-2, anti pLDH spécifique de *P.vivax*, anti pan pLDH commun aux quatre espèces et un anticorps anti lapin fixés au niveau des différentes bandes tests et contrôles.
Une pipette capillaire
Un sachet déssicant
Un flacon contenant le tampon de lyse

MATERIEL NECESSAIRE MAIS NON FOURNI
Chronomètre avec alarme
Pipette de laboratoire (optionnel)
Lancettes stériles (optionnel)

RECUEIL ET CONSERVATION DE L'ECHANTILLON
Le sang capillaire ou veineux peut être utilisé. Nettoyer la peau avec une solution antiseptique et attendre que la zone sèche avant de prélever. Dans le cas de recueil de sang veineux, un tube hépariné, oxalaté ou à EDTA peut être utilisé car aucun de ces anticoagulants n'interagit avec le test. En cas d'impossibilité de réaliser le test immédiatement, le sang total peut être conservé au maximum 72 heures à 2-8°C.

PRECAUTIONS

Pour diagnostic *in vitro* uniquement. Ne pas utiliser après la date de péremption.
Ne pas manger, boire ou fumer lors de la manipulation des échantillons et du test.
Ne pas pipeter à la bouche.
Les échantillons de sang doivent être considérés comme potentiellement infectieux. Lors de la réalisation du test, prendre les précautions nécessaires à la manipulation de produits infectieux. Traiter ensuite les différents éléments du test et les échantillons selon la procédure réservée aux déchets potentiellement infectieux.
Se munir d'une blouse, de gants et de protection oculaire lors de la réalisation du test.

Se nettoyer les mains efficacement après réalisation du test.
Ne pas interchanger les réactifs de différents lots.

PROCEDURE COMPLEMENTAIRE DE CONTROLE DE QUALITE

Les bonnes pratiques de laboratoire préconisent l'utilisation de contrôles positifs et négatifs pour vérifier le bon fonctionnement du test. Ceux-ci sont disponibles séparément.

CONSERVATION ET STABILITE

La trousse peut être conservée à température ambiante ou réfrigérée (4-30°C). La savonnette est stable jusqu'à la date de péremption indiquée sur le sachet. Elle doit être conservée dans son sachet jusqu'à utilisation. **NE PAS CONGELER.** Ne pas utiliser au-delà de la date de péremption.

PERFORMANCES

Une étude réalisée sur un panel de 251 échantillons confirmés au préalable par un examen microscopique a donné les résultats suivants :

Echantillon	Nombre d'échantillons testés	**PALUTOP+4**		Sensibilité	Spécificité
		Positif	Négatif		
P. *falciparum*	16	16	0	100%	-
P. *vivax*	25	25	0	100%	-
Négatif	210	0	210	-	100%

PROCEDURE

Laisser revenir les composants de la trousse à température ambiante. En cas de conservation à 2-8°C, attendre 30 minutes pour retour à température ambiante. Vérifier la couleur du déssicant. Celui-ci doit être bleu. Si celui-ci est incolore ou bleu pâle, utiliser une autre savonnette.
Une fois le sachet aluminium ouvert, le test doit être réalisé immédiatement.
Prélèvement et recueil de l'échantillon :
a) Protocole sur sang capillaire
Sélectionner la zone de ponction (habituellement le côté du 3ème ou du 4ème doigt). Nettoyer la zone avec une solution antiseptique et attendre qu'elle sèche. Inciser le doigt avec une lancette stérile. Attendre la formation d'une goutte de sang. Poser la pipette capillaire sur la goutte de sang. Transférer le sang dans **le puits échantillon A** par contact de la pipette capillaire. Vérifier que le sang n'a pas coagulé avant transfert. Vérifier que la totalité du sang contenu dans la pipette capillaire a été déposé au niveau du puits A.
b) Protocole sur sang veineux
En cas d'utilisation de sang veineux, agiter le tube avant de prélever la prise d'essais. 5 à 10 µl de sang doit être déposé dans le **puits échantillon A**, soit avec une pipette de laboratoire, soit avec la pipette capillaire fournie (vérifier que la totalité du sang contenu dans la pipette capillaire a été déposé au niveau du puits A).
Déposer 6 gouttes (300 µl) de tampon de lyse dans le puits B en tenant le flacon verticalement.
Lire le résultat à 15 minutes. Ne pas interpréter le résultat au delà de 15 minutes.

INTEPRETATION DU RESULTAT

NEGATIF pour les quatre espèces :
Une seule bande mauve apparaît au niveau de la région 'C'.

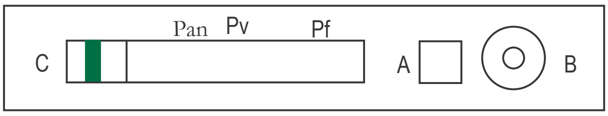

POSITIF pour au moins une des quatre espèces :

P. falciparum : **En plus de la bande contrôle, une bande mauve apparaît au niveau des régions 'Pf' et 'Pan' respectivement. Dans certains cas d'infection à *P. falciparum,* (phase post traitement notamment), la bande Pan peut être absente.**

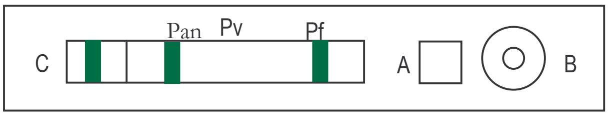

P.vivax : **En plus de la bande contrôle, une bande mauve apparaît au niveau des régions 'Pv' et 'Pan' respectivement.**

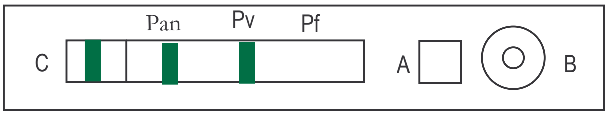

Autres espèces: **En plus de la bande contrôle, une bande mauve apparaît au niveau de la région 'Pan'**

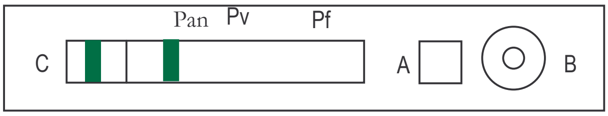

Infections mixtes : **En plus de la bande contrôle, une bande mauve apparaît au niveau des régions 'Pf', 'Pv' et 'Pan' respectivement.**

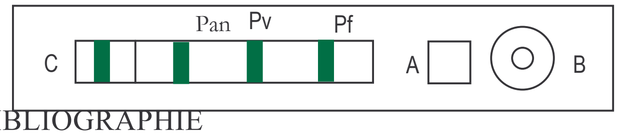

BIBLIOGRAPHIE

1. Howard, R.J., et al, 1986: Secretion of a Malarial Histidine-rich Protein (Pf. HRP II) from Plasmodium falciparum-infected Erythrocytes.J. Cell Biol., 103, 1269-1277. 2.Rock, E.P., et al, 1987: Comparative Analysis of the Plasmodium falciparum Histidine-Rich Proteins HRP-I, HRP-II, and HRP-III in Malaria Parasities of Diverse Origin. Parasitol., 95, 209-227. 3. Parra, M.E., et al, 1991: Identification of Plasmodium falciparum Histidine-Rich Protein 2 in the Plasma of Humans with Malaria.J. Clin. Microbiol., 29, 1629-1634. 4. Rodriguez-Del Valle, M., et al, 1991: Detection of Antigens and Antibodies in the Urine of Humans with Plasmodium falciparum Malaria. J. Clin. Microbiol., 29, 1236-1242. 5. Makler, M. T., et. al.(1993) Parasite lactate assay as an assay for *Plasmodium falciparum* drug sensitivity. Am. J. Trop. Med. Hyg. 48(6), 739-741. 6.Piper, R. C., et. al., (1999) Immuno-capture diagnostic assays for malaria utilizing *Plasmodium* Lactate Dehydrogenase (pLDH) Am. J. Trop. Med.Hyg. 60(1) 109-118. 7. Srinivasan. S., et. al.,(2000) Comparison of blood – film microscopy, The OptiMAL dipstick, Rhodamine- 123 fluorescence staining and PCR for monitoring antimalarial treatment. Annals of Tropical Medicine and Parasitology, 94(3) 227-232. 8. Hunte-Cooke A., et. al., (1999) Comparison of a Parasite Lactate Dehydrogenase-based Immunochromatographic Antigen Detection assay (OptiMAL®) with Microscopy for the Detection of Malaria Parasites in Human Blood Samples. Am J.Trop Med 60(2). 173-176. 9. John, S. M., et. al.,(1998) Evaluation of OptiMAL,a dipstick test for the diagnosis of malaria. Ann. Trop. Med. Parasitol., 92, 621-622. 10. Quintana M., et. al.,(1998) Malaria diagnosis by dipstick assay in a Honduran Population with coendemic *Plasmodium falciparum* and *Plasmodium vivax*. Am. J. Trop. Med. Hyg. 59(6) 868-871. 11.Palmer, C. J.,((1998) Evaluation of OptiMal test for rapid diagnosis of *Plasmodium vivax* and *Plasmodium falciparum* . J. Clin Microbiol . 36(1) 203-206. 12. Moody A., et. al (2000) Performance of the OptiMAL® malaria

antigen capture dipstick for malaria diagnosis and treatment monitoring. British Journal of Hematology, 109, 1-5 . 13 Data on file: Zephyr Biomedicals.

Directive 98/79/CE Le 19/05/2004 Version 1

ALL DIAG INSTRUMENTS ET RÉACTIFS ISO 9001 ISO 13485	**ALL DIAG**<br>**10, RUE ETTORE BUGATTI - BP6**<br>**67038 STRASBOURG CEDEX 2**<br>**Tél : 03.88.78.80.88.**<br>**Fax : 03.88.78.76.78**<br>www.alldiag.com – info@alldiag.com

ANNEXE 4 : fiche technique du test de diagnostic rapide Core Malaria Pan/Pv/Pf

CE IVD

Core Malaria Pan/Pv/Pf 25 REF *MAL-190026*

Détection rapide et qualitative des *Plasmodium*

2005-02-23/FR

1. **INTRODUCTION**

Core Malaria Pan/Pv/Pf est un test de diagnostic rapide *in vitro* pour la détection de *P.falciparum*, *P.vivax* et autres espèces de plasmodium (Pan) dans le sang. Ce test détecte la protéine HRP-2 spécifique de *P.falciparum*, pLDH spécifique de *P.vivax* et Pan pLDH spécifique des autres espèces de plasmodium. Il est utilisé pour la détection et la différenciation entre les différents parasites ainsi que pour le suivi thérapeutique.

2. **RESUME**

Quatre espèces de Plasmodium sont responsables de l'infection du paludisme chez l'homme : *P.falciparum*, *P.vivax*, *P.ovale* et *P.malariae*. Parmi ces quatre, *P.falciparum* et *P.vivax* sont les plus répandues. Une détection et identification précoce du parasite est extrêmement importante à cause des incidences cérébrales, de la résistance aux antipaludéens et de la mortalité. La différenciation entre *P.falciparum* et *P.vivax* est très importante pour adapter la meilleure thérapie possible.

3. **PRINCIPE**

Le test Core Malaria Pan/Pv/Pf est un test immuno-chromatographique basé sur le principe du sandwich. Il contient les réactifs immunologiques déshydratés suivants : conjugué anti-HRP-2 / or colloïdal, conjugué anti-*P.vivax*-pLDH / or colloïdal, conjugué anti-Pan-pLDH / or colloïdal, anticorps anti-HRP-2 immobilisé, anticorps anti-*P.vivax*-pLDH spécifique immobilisé, anticorps anti-Pan immobilisé, anticorps anti-lapin immobilisé.

Le test débute par le dépôt d'une goutte de sang dans le puits marqué (A) (l'échantillon est prélevé à l'aide d'une anse calibrée fournie). Afin de faciliter la lyse des globules rouges (hémolyse) ainsi que la migration du sang à travers la membrane, 4 gouttes de solution tampon sont déposées dans le puits marqué (B). L'échantillon migre alors par chromatographie et entraîne les conjugués.

En cas d'échantillon positif, l'HRP-2 ou pLDH est immobilisé en sandwich entre le conjugué et l'anticorps spécifique sur la ligne test (zone T) correspondante. Les excès de conjugué continuent de migrer et sont immobilisés tardivement par l'anticorps anti-lapin sur la ligne de contrôle (C). L'accumulation de complexes colorés entraîne le développement des lignes parallèles colorées en rose.

La ligne Contrôle (C) valide le bon fonctionnement du test ainsi qu'une manipulation correcte.

Les lignes Pan, Pv, Pf indiquent les résultats positifs.

En cas de résultat négatif, aucune ligne test ne se forme et seule la ligne Contrôle (C) est visible.

4. **COMPOSITION**

Un coffret Core Malaria Pan/Pv/Pf contient :

- 25 tests emballés individuellement (avec sachet déshydratant).
- 1 anse calibrée fournie à l'intérieur de chaque test.
- 1 flacon compte-gouttes de tampon
- Une notice d'utilisation.
- Note : Une micropipette distribuant 5 µl peut être utilisée à la place de l'anse calibrée.

5. **CONSERVATION**

- Conservé dans son emballage d'origine à +4°C / +30°C, le test Core Malaria Pan/Pv/Pf est stable jusqu'à la date de péremption indiquée sur chaque sachet aluminium ou sur la boîte.
- Ne pas congeler.
- Sortir le test du sachet uniquement avant son utilisation immédiate.

6. **PRECAUTIONS D'EMPLOI**

- Lire attentivement les instructions avant de pratiquer le test.
- Pour diagnostic *in vitro* uniquement.
- Ne pas utiliser après la date de péremption.
- Ne pas mélanger les composants issus de différents lots.
- Manipuler les échantillons comme potentiellement infectieux.
- Respecter les règles de sécurité inhérentes à la manipulation de matériel et produits potentiellement infectieux.

7. **PRELEVEMENT ET CONSERVATION DES ECHANTILLONS**

- Le sang total prélevé dans un tube additionné d'anti-coagulant devra être utilisé comme un échantillon test et les anti-coagulants suivants : EDTA ou Héparine ou encore Oxalate peuvent être utilisés.
- Si L'échantillon collecté n'est pas immédiatement utilisé, il devra être conservé entre +2°C / + 8°C jusqu'à 72 heures.
- Les échantillons coagulés ou suspectés d'avoir été contaminés ne devront pas être utilisés.
- Le sang frais obtenu en piquant l'extrémité du doigt peut être utilisé et prélevé à l'aide de l'anse calibrée fournie.

8. **MATERIEL REQUIS NON FOURNI**

- Chronomètre ;
- Récipient pour déchets biologiques.

9. **MODE OPERATOIRE ET INTERPRETATION DES RESULTATS**

Plusieurs tests peuvent être effectués en même temps. Il est recommandé d'utiliser les tests dès leur ouverture. Après avoir ramené les échantillons et les tests à température ambiante procéder comme suit :

1. Identifier l'échantillon à tester et noter la date du test.
2. A l'aide de l'anse calibrée fournie, prélever l'échantillon de sang et déposer le dans le puits marqué (A). Une micropipette calibrée à 5µl peut être utilisée.

Précautions :

- Les prélèvements de sang sur anti-coagulant sont mélangés par agitation douce, avant utilisation.
- Avant de transférer une goutte de sang sur l'adsorbant de la fenêtre (A), s'assurer que la boucle de l'anse calibrée est pleine de sang (prélèvement sur anti-coagulant, ou après piqûre au doigt).
- Le transfert du sang doit être effectué immédiatement après recueil de la goutte.
- Vérifier que la goutte de sang déposée est complètement récupérée par l'adsorbant (A).

3. Déposer ensuite 4 gouttes de solution tampon dans le puits marqué (B).
4. Au terme de 15 minutes, interpréter les résultats comme suit :

Résultat négatif :
Une seule ligne colorée apparaît dans la région (C) correspondant au contrôle du test.

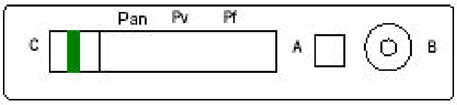

Résultats positifs :
P.falciparum : En plus de la ligne de contrôle (C) apparaît une ligne parallèle au niveau de la région "Pf" et "Pan" correspondant au résultat du test.

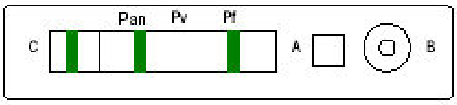

P.vivax : En plus de la ligne de contrôle (C) apparaît une ligne parallèle au niveau de la région "Pv" et "Pan" correspondant au résultat du test.

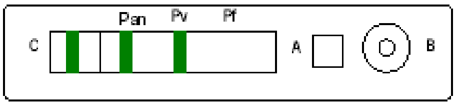

Autres espèces : En plus de la ligne de contrôle (C) apparaît une seconde ligne parallèle au niveau de la région "Pan".

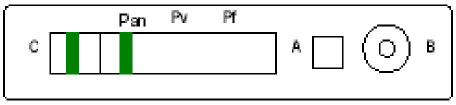

Infection mixte : En plus de la ligne de contrôle (C) apparaissent des lignes parallèles au niveau des régions "Pan", "Pv" et "Pf"

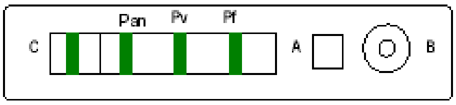

Le test doit être considéré comme invalide si aucune ligne n'apparaît. Refaire le test avec une autre cassette.

10. LIMITES DU TEST

- Comme pour tous les tests diagnostiques, les résultats doivent toujours être corrélés avec les signes cliniques.
- Les résultats du test doivent être interprétés dans le contexte épidémiologique, clinique, et thérapeutique. Quand cela semble indiqué, les techniques parasitologiques de référence doivent être considérées (examen microscopique de la goutte épaisse et frottis sanguins).
- Toute modification du mode opératoire ci-dessus et/ou l'utilisation d'autres réactifs invalident la procédure du test.
- Les cassettes et tampons de différents lots ne peuvent pas être mélangés.
- Dans le cas d'infection par *P. vivax* ou *P. falciparum* ou d'infection mixte par ces espèces, la bande "Pan" sera aussi positive. La différenciation d'une infection causée par *P. ovale* ou *P. malariae* ne peut être faite.
- Lors d'un traitement, si le résultat du test reste positif avec la même intensité après 5 à 10 jours de traitement, la possibilité d'une souche résistante de malaria doit être considérée.
- Généralement les bandes "Pv" et "Pan" deviennent négatives après un traitement anti- paludéen réussi. La durée du traitement et le médicament utilisé affectent l'élimination des parasites ; le test doit être répété 5 à 10 jours après le début du traitement.
- Dans l'infection par *P. falciparum*, la protéine HRP-2 n'est pas secrétée au stade de gamétogonie. Donc chez les « porteurs », la bande HRP-2 peut être absente.
- Dans le cas de malaria à *P. falciparum*, les taux de HRP-2 peuvent persister jusqu'à 15 jours après le traitement ; dans ce cas la disparition de la bande "Pan" qui peut être présente avant traitement disparaîtra, témoin de l'efficacité thérapeutique.
- Dans quelques cas, la bande HRP-2 est positive et la bande "Pan" malaria négative. Cela peut traduire une infection post thérapeutique ; une telle configuration peut aussi être obtenue dans quelques cas de paludisme non traité. Dans ces cas là, il est conseillé de refaire un test 2 jours après.

11. CARACTERISTIQUES DU TEST

Dans une étude interne, un panel de 251 échantillons dont les résultats ont été confirmés par microscopie a été étudié avec le test Core Malaria. Les résultats obtenus ont été les suivants :

Echantillons	Echantillons testés	Core Malaria Pan/Pv/Pf		Sensibilité	Spécificité
		Positif	Négatif		
P. falciparum +Ve	16	16	0	100%	-
P. vivax +Ve	25	25	0	100%	-
Malaria -Ve	210		210	-	100%

12. BIBLIOGRAPHIE

1. **Howard, R.J., et al.** 1986. Secretion of a Malarial Histidine-rich Protein (Pf. HRP II) from Plasmodium falciparum-infected Erythrocytes. J. Cell Biol. **103:**1269-1277.
2. **Rock, E.P., et al.** 1987. Comparative Analysis of the Plasmodium falciparum Histidine-Rich Proteins HRP-I, HRP-II, and HRP-III in Malaria Parasities of Diverse Origin. Parasitol. **95:** 209-227.
3. **Parra, M.E., et al.** 1991. Identification of Plasmodium falciparum Histidine-Rich Protein 2 in the Plasma of Humans with Malaria. J. Clin. Microbiol. **29:** 1629-1634.
4. **Rodriguez-Del Valle, M., et al.** 1991. Detection of Antigens and Antibodies in the Urine of Humans with Plasmodium falciparum Malaria. J. Clin. Microbiol. **29:**1236-1242.
5. **Makler, M. T., et. al.** 1993. Parasite lactate assay as an assay for *Plasmodium falciparum* drug sensitivity. Am. J. Trop. Med. Hyg. **48(6):** 739-741.
6. **Piper, R. C., et. al.** 1999. Immuno-capture diagnostic assays for malaria utilizing *Plasmodium* Lactate Dehydrogenase (pLDH). Am. J. Trop. Med. Hyg. **60(1):** 109-118.
7. **Srinivasan, S., et. al.** 2000. Comparison of blood – film microscopy, The OptiMAL dipstick, Rhodamine-123 fluorescence staining and PCR for monitoring antimalarial treatment. Annals of Tropical Medicine and Parasitology, **94(3):** 227-232.
8. **Hunte-Cooke, A., et. al.** 1999. Comparison of a Parasite Lactate Dehydrogenase-based Immunochromatographic Antigen Detection assay (OptiMAL®) with Microscopy for the Detection of Malaria Parasites in Human Blood Samples. Am J.Trop. Med. **60(2):** 173-176.
9. **John, S. M., et. al.** 1998. Evaluation of OptiMAL, a dipstick test for the diagnosis of malaria. Ann. Trop. Med. Parasitol. **92:** 621-622.
10. **Quintana, M., et al.** 1998. Malaria diagnosis by dipstick assay in a Honduran Population with coendemic *Plasmodium falciparum* and *Plasmodium vivax*. Am. J. Trop. Med. Hyg. **59(6):** 868-871.
11. **Palmer, C. J.** 1998. Evaluation of OptiMal test for rapid diagnosis of *Plasmodium vivax* and *Plasmodium falciparum* . J. Clin Microbiol . **36(1):** 203-206.
12. **Moody, A., et al.** 2000. Performance of the OptiMAL® malaria antigen capture dipstick for malaria diagnosis and treatment monitoring. Brit. J. Hematol. **109:** 1-5 .

13. SYMBOLES UTILISES

IVD	*In Vitro* diagnostic	LOT	Numéro de lot
REF	Référence, catalogue		Date de péremption
	Fabriqué par		Date de fabrication
25	Nombre de tests (25)	4°C – 30°C	Conserver à +4°C / + 30°C
i	Notice dans la trousse, Information à lire		
PIPETTE	Système de prélèvement (dans le sachet)		
CARD	Cassette (dans le sachet)		

CORE DIAGNOSTICS Ltd
Aspect Court 4, Temple Row
Birmingham, B2 5HG United Kingdom

2005-02-23/FR
