# Supplementary material for: Performance of Rapid Diagnostic Tests for Imported Malaria in Clinical Practice: Results of a National Multicenter Study
Source: PLoS One. 2013 Sep 30;8(9):e75486. doi: 10.1371/journal.pone.0075486 (PMC3787089; doi:10.1371/journal.pone.0075486)
Supplement: Protocol summary S1 — Summary of the research protocol. (DOC) [file pone.0075486.s005.doc]

Protocol Summary

**Multicentric evaluation of RDTs for the diagnosis of imported malaria**
**TDR malaria AOR 06 066**

Coordinating investigator: Professor Sophie Matheron

Chief scientist: Dr Sandrine Houzé

**1. Literature data and scientific background**

In France, imported malaria is a disease that affects travelers and migrant populations returning to their countries with contamination in sub-Saharan Africa in 95% of cases. It is estimated that from 5000 to 8000 cases of malaria are reported each year in France with twenty deaths annually. *Plasmodium falciparum*, the causative agent in 85% of access, is potentially lethal. Clinical signs of malaria are nonspecific and diagnosis can be easily mistaken to a seasonal viral infection. Malaria is a serious disease that must be diagnosed urgently. The reference routine test is microscopic examination of Giemsa-stained blood smears. However, many centers cannot provide reliable round-the-clock smear-based diagnosis.

The rapid diagnostic tests (RDTs) for malaria that detect soluble antigens are available for malaria diagnosis especially in medical centers without available microscopic diagnosis. However, the results of studies evaluating the performance of these tests are contradictory.

Different antigens are available for diagnosis such as PfHRP2, *Plasmodium falciparum* histidin rich protein 2 or PfLDH, *Plasmodium falciparum* lactate dehydrogenase, both specific for *Plasmodium falciparum*; pLDH, plasmodium lactate dehydrogenase and aldolase antigens enzymes shared by the 5 human pathogenic *Plasmodium* species.

**2 Methodologies**

This is a study to evaluate the diagnosis performance of RDT on samples from patients of all ages, return malaria endemic country and presenting with suspected malaria in emergency wards of sites participating in the study.
This study will be conducted in accordance with defined quality criteria for diagnostic studies of evolution within the STARD initiative (Standard for Reporting of Diagnostic Accuracy) and tool QUADAS (Quality Assessment of Diagnostic Accuracy Studies of included in systematic reviews). All enrolled patients will benefit from two types of tests: rapid diagnostic tests for malaria diagnosis and examination of reference or "gold standard" thin and thick films examined by experienced microscopist. These tests will be evaluated independently of the other (blind test results of the other).

**3. Objective**

The main objective is to determine the sensitivity, specificity, positive predictive value, negative predictive value ​​and likelihood ratio of detection of each antigen.

**4. Material**

Four rapid diagnostic tests detecting different antigens: Now ® ICT Malaria (Inverness Medical), Palutop +4 ® (All Diag), Core Malaria Pan-Pv-Pf (Core Diagnostics), OptiMAL-IT (Diamed) will be tested in double-blind and parallel in 9 French hospital centers on blood samples from suspected malaria patients compared to thin smear and thick smear made as a reference.

**5. Search time and duration of subject's participation**

The study will be conducted in 9 centers in France; it will include approximately 1225 patients to study 245 patients with malaria. This research will begin in January 2007 and will last one year. The participation of each subject will be the time of hospital consultation.

5.1 Inclusion criteria

Patients attending a participating center with symptoms leading to prescription of diagnostic laboratory tests for malaria will be prospectively considered for enrollment. No additional sample will be made for the study that this protocol is a non-interventional research. The patient will receive written information about the research before inclusion.

5.2 Expected results

Performances reported by manufacturers are between 93.4% and 100% for sensitivity and 96% to 100% for specificity. However, a preliminary study in the parasitology laboratory of the Bichat hospital in Paris, showed sensitivities between 67% and 90% depending on the test. This new multicenter study will determine the performance of these tests in the studied population and possibly offer an optimal combination of detected antigens for manufacturers of medical devices.
